# Addition of lithiated enol ethers to nitrones and subsequent Lewis acid induced cyclizations to enantiopure 3,6-dihydro-2*H*-pyrans – an approach to carbohydrate mimetics

**DOI:** 10.3762/bjoc.6.75

**Published:** 2010-07-09

**Authors:** Fabian Pfrengle, Hans-Ulrich Reissig

**Affiliations:** 1 Freie Universität Berlin, Institut für Chemie und Biochemie, Takustrasse 3, D-14195 Berlin, Germany

**Keywords:** aminopyrans, carbohydrate mimetics, Lewis acids, lithiated enol ethers, nitrones, oxidative cleavage, stereodivergent synthesis

## Abstract

A stereodivergent synthesis of enantiopure 3,6-dihydro-2*H*-pyrans is presented. The addition of lithiated enol ethers to carbohydrate-derived nitrones afforded *syn*- or *anti*-configured hydroxylamine derivatives **4a**–**d** that were cyclized under Lewis acidic conditions to yield functionalized dihydropyrans *cis*- or *trans*-**5a**–**d** containing an enol ether moiety. This functional group was employed for a variety of subsequent reactions such as dihydroxylation or bromination. Bicyclic enol ether **19** was oxidatively cleaved to provide the highly functionalized ten-membered ring lactone **20**. The synthesized enantiopure aminopyrans **24**, **26**, **28** and **30** can be regarded as carbohydrate mimetics. Trimeric versions of **24** and **28** were constructed via their attachment to a tricarboxylic acid core.

## Introduction

**Scheme 1:**
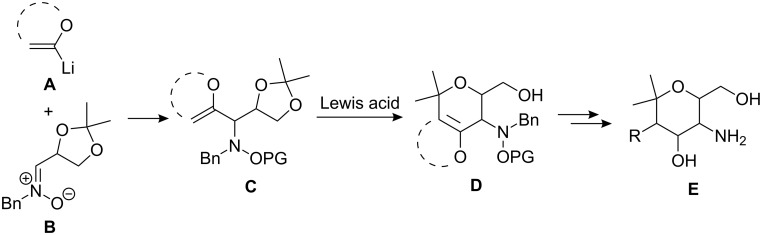
Synthesis of 3,6-dihydro-*2H*-pyrans **D**.

The pyran structural motif can be found in numerous bioactive natural products. Possible strategies towards their efficient preparation include Prins cyclizations, intramolecular substitutions, ring closure metathesis, and hetero Diels–Alder reactions [1–2]. Our group recently reported the synthesis of enantiopure aminopyrans employing as the key step a Lewis acid induced rearrangement of 1,2-oxazines to bicyclic ketones. This strategy allowed a simple access to unusual carbohydrates with C2-branched 4-amino sugar units and related carbohydrate mimetics [3–7]. Moreover, pursuing a similar strategy, we described the stereocontrolled preparation of 3,6-dihydro-*2H*-pyrans **D** as precursors for the synthesis of enantiopure aminopyrans **E** (Scheme 1). The sequence of nucleophilic addition of lithiated enol ethers **A** to nitrones **B** and Lewis acid promoted cyclization of the resulting 1,3-dioxolanyl-substituted hydroxylamine derivatives **C**, delivered the enantiopure dihydropyrans **D** in a highly stereodivergent fashion [8]. In this report we disclose full details about this new method, including expansion of the reaction scope and further evaluation of the synthetic potential of the easily accessible functionalized dihydropyrans **D**.

## Results and Discussion

The addition of organometallic species to nitrones has been extensively studied in the past. Highly stereoselective reactions of enantiopure nitrones allow rapid access to functionalized intermediates that have been elegantly used for the preparation of a wide range of natural products [9]. It is known that pre-complexation of carbohydrate-derived nitrones by Lewis acids can reverse the stereochemical outcome of these reactions [10] allowing, for example, a stereodivergent access to enantiopure 1,2-oxazines [11].

When lithiated enol ethers **1** were used as the organometallic species in the addition to nitrones **2** [12–14], either *syn*- or *anti*-configured hydroxylamine derivatives **3** were obtained, depending strongly on the reaction conditions (Table 1) [15–16]. Subsequent treatment under basic conditions with *tert*-butyldimethylsilyl triflate afforded TBS-protected compounds *syn*- and *anti*-**4** in good overall yields [17]. The stereochemical outcome of the reaction is in line with the addition of other nucleophiles to nitrone **2a** [9–11]. The configurations of compounds **4** could be unambiguously assigned by X-ray crystallographic analysis of an aminopyran derivative derived from *anti*-**4b** (compound **29**, Scheme 12) [8]. The enol ethers used, i.e., ethyl vinyl ether, dihydropyran, and dihydrofuran, all added smoothly to glyceraldehyde-derived nitrones **2a** or **2b** after lithiation with *tert*-butyllithium. Whereas in the reaction with uncomplexed nitrones, 1.5 equivalents of the respective lithiated enol ether were sufficient to obtain the desired *syn*-products with good yields and stereoselectivities, 5 equivalents of the nucleophile were necessary to achieve reasonable yields in the addition to the nitrone/chlorodiethylaluminium complexes. Despite this, diastereomeric ratios in favour of the *anti*-products were generally excellent.

**Table 1:** Stereodivergent additions of lithiated enol ethers **1** to glyceraldehyde-derived nitrones **2**.

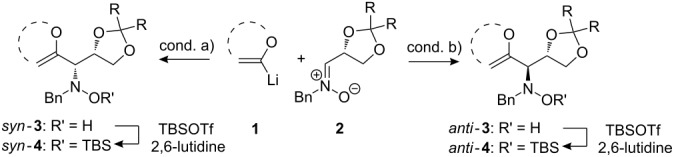				
Lithiated enol ether	Nitrone	Conditions^a^	Product	Yield and diastereomeric ratio

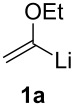	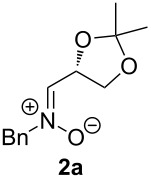	a)	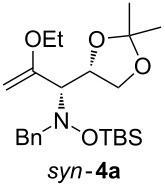	*syn*: 61% *anti*: 9% (d.r. = 88:12)
**1a**	**2a**	b)	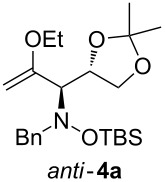	*syn*: 4% *anti*: 48% (d.r. = 93:7)
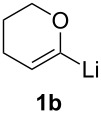	**2a**	a)	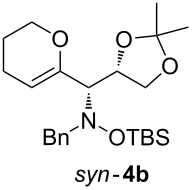	*syn*: 44% *anti*: 18% (d.r. = 72:28)
**1b**	**2a**	b)	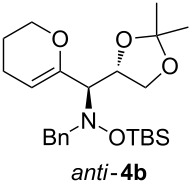	*syn*: 8% *anti*: 56% (d.r. = 88:12)
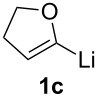	**2a**	a)	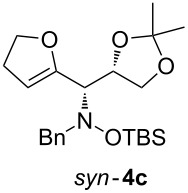	*syn*: 58% *anti*: 10% (d.r. = 86:14)
**1c**	**2a**	b)	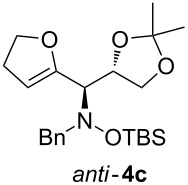	*syn*: 9% *anti*: 54% (d.r. = 86:14)
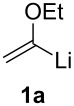	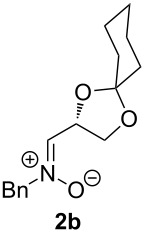	a)	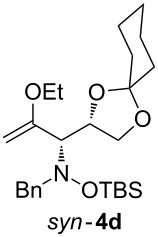	*syn*: 70% (d.r. > 95:5)

^a^Condition a): 1. THF, −78 °C, 1 h (1.5 equiv of enol ether); 2. TBSOTf, 2,6-lutidine, 30 min; Condition b): 1. **2** + Et_2_AlCl, THF, 5 min, then addition to **1**, THF, −78 °C, 15 min (5 equiv of enol ether); 2. TBSOTf, 2,6-lutidine, 30 min.

**Scheme 2:**
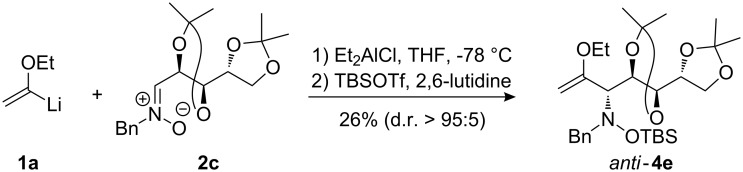
Addition of lithiated enol ether **1a** to nitrone **2c**/Et_2_AlCl.

Lithiated ethyl vinyl ether was also added to pre-complexed D-arabinose-derived nitrone **2c** (Scheme 2). After a subsequent TBS-protection step, the desired hydroxylamine derivative *anti*-**4e** was obtained in low yield, but with excellent diastereoselectivity.

Next, the protected hydroxylamine derivatives *syn*-**4** and *anti*-**4** were treated with Lewis acid to provide enantiopure 3,6-dihydro-*2H*-pyrans *cis*- and *trans*-**5** (Table 2). The cyclization proceeded in moderate to excellent yields when two equivalents of trimethylsilyl triflate were used as Lewis acid. Catalytic amounts of Lewis acid were not sufficient to obtain the dihydropyrans, even after prolonged reaction times. The reactions with stoichiometric amounts of TMSOTf gave monocyclic, bicyclic, and spirocyclic dihydropyrans depending on the 1,3-dioxolanyl-substituted enol ether employed (Table 2). Unfortunately, attempts to generate an eight-membered (or six-membered) ring by cyclization of *anti*-**4e** were unsuccessful, only decomposition was observed.

**Table 2:** Lewis acid promoted cyclization of 1,3-dioxolanyl-substituted enol ethers *syn*- and *anti*-**4** to 3,6-dihydro-*2H*-pyrans *cis*- and *trans*-**5**.

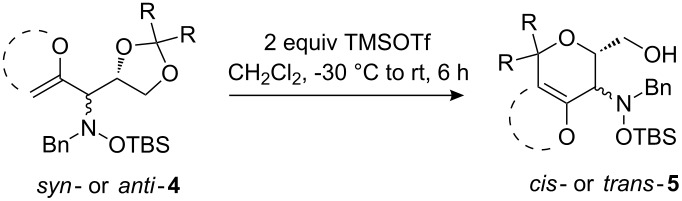		
Cyclization precursor	Product	Yield

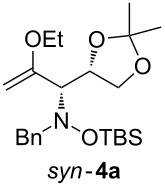	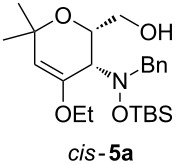	79%
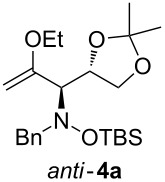	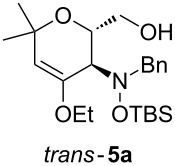	84%
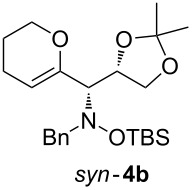	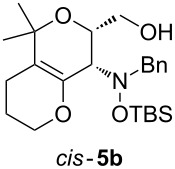	74%
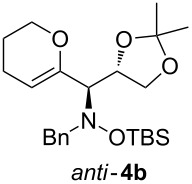	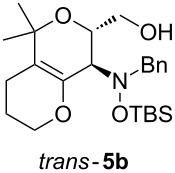	82%
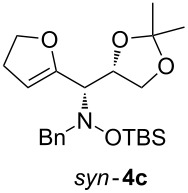	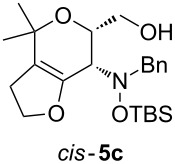	85%
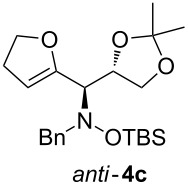	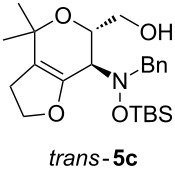	55%
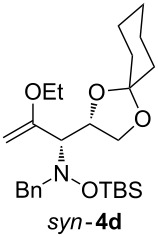	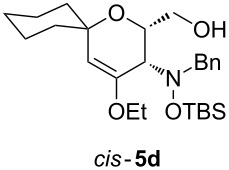	90%
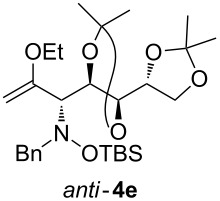	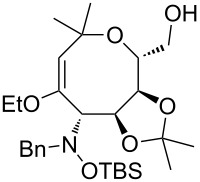	–

**Scheme 3:**
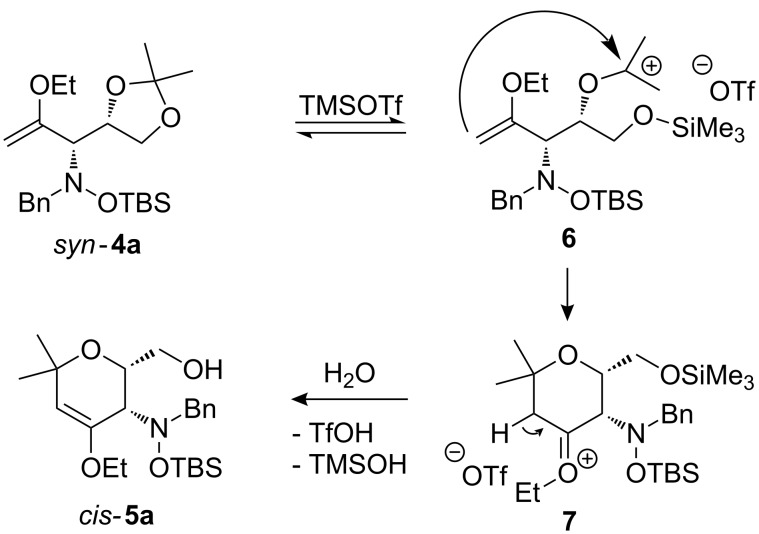
Mechanistic proposal for the transformation of *syn*-**4a** into *cis*-**5a** in the presence of TMSOTf.

As a mechanistic rationale, shown below for the transformation of *syn*-**4a** into *cis*-**5a**, we propose that the Lewis acid coordinates to the distal oxygen atom of the dioxolane moiety in **4** leading to a ring opening and formation of a stabilized carbenium ion **6** (Scheme 3). Subsequent attack of the oxocarbenium ion on the enol ether moiety leads to ring closure affording the cationic pyran intermediate **7**. Subsequent proton transfer to the (moderately basic) hydroxylamine nitrogen re-establishes the enol ether moiety. During aqueous work-up the OTMS-group is apparently removed due to the fairly acidic conditions to produce *cis*-**5a**. Overall, the described cyclization can be classified as an aldol-type reaction of an enol ether to an acetal, or as a Prins-type reaction.

**Scheme 4:**
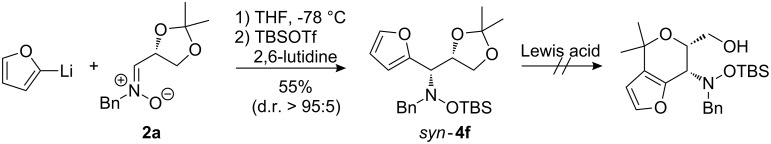
Unsuccessful attempt to cyclize hydroxylamine derivative *syn*-**4f**.

Encouraged by these results, we decided to try a related Friedel–Crafts-type cyclization. Thus, 2-lithiofuran was added to nitrone **2a** [18] and then protected with TBSOTf to give hydroxylamine derivative *syn*-**4f** in 55% (Scheme 4). Remarkably, subsequent treatment with 2 equiv of TMSOTf did not lead to the desired bicyclic product. Since no conversion was observed, the lower reactivity of *syn*-**4f**, caused by the considerably weaker nucleophilicity of the furan compared to an enol ether [19], might in the future be overcome with more forcing reaction conditions.

**Scheme 5:**
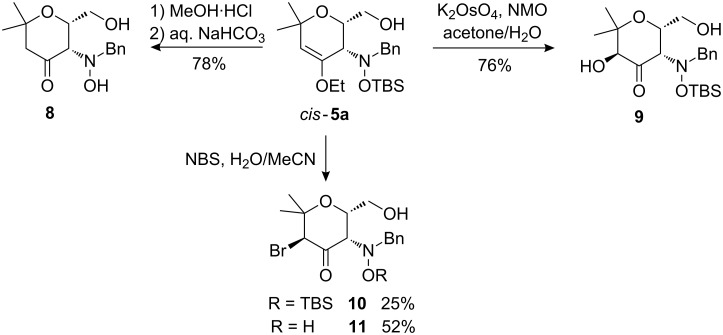
Functionalizations of the enol ether moiety of *cis*-**5a** leading to compounds **8**–**11**.

**Scheme 6:**
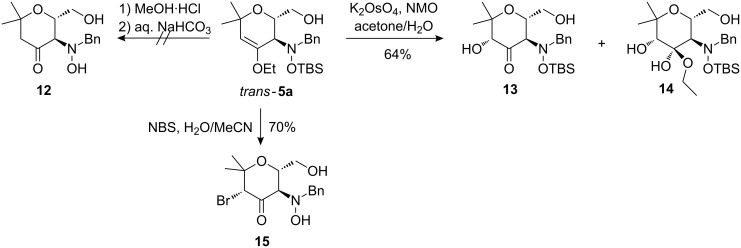
Transformations of the enol ether moiety of *trans*-**5a** leading to products **13**–**15**.

The enol ether moiety in dihydropyrans **5** represents a very versatile handle for further synthetic modifications (Scheme 5 and Scheme 6). Starting from *cis*-**5a**, acidic hydrolysis with concurrent loss of the TBS-group by treatment with saturated methanolic HCl followed by the addition of a saturated solution of NaHCO_3_ in water gave ketone **8** in good yield (Scheme 5 and Scheme 6). Dihydroxylation using the K_2_OsO_4_/*N*-methylmorpholinoxide-system afforded α-hydroxyketone **9** via in situ hydrolysis of an initially formed hemiacetal. Bromination with NBS in MeCN/H_2_O led to α-bromoketone **10** and its desilylated derivative **11** as single diastereomers. We presume that the NBS attacks the enol ether moiety, as is also the case with the dihydroxylation reagent OsO_4_, from the sterically less hindered side, leading to incorporation of the bromo substituent *trans* to the hydroxymethyl and the bulky hydroxylamine moiety. The partial removal of the TBS-group is undoubtedly caused by HBr formed in the course of the reaction.

When these reaction conditions were applied to the diastereomeric dihydropyran *trans*-**5a**, different behaviour was noted (Scheme 6). A smooth hydrolysis of the enol ether moiety leading to ketone **12** was neither feasible with methanolic HCl nor with aqueous HCl. After dihydroxylation, a partial hydrolysis of the hemiacetal initially formed in the dihydroxylation was observed leading to an inseparable mixture of compounds **13** and **14**. The TBS-protection group was completely removed during bromination of the enol ether moiety with the formation of compound **15** in good yield [20].

**Scheme 7:**
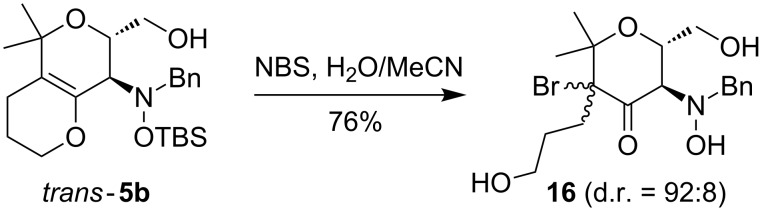
Bromination of bicyclic dihydropyran *trans*-**5b** affording **16**.

The internal enol ether moiety of the bicyclic compound *trans*-**5b** could also be converted into the corresponding α-bromoketone with the bromination reagent NBS to give the ring opened product **16** (Scheme 7). The two possible diastereomers were formed in a ratio of 92:8, as indicated by ^1^H NMR spectroscopy and have not been separated.

**Scheme 8:**
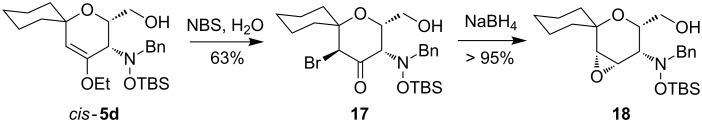
Synthesis of epoxypyran **18** by bromination of *cis*-**5d**.

Bromination of the cyclohexylidene-substituted dihydropyran *cis*-**5d** afforded α-bromo ketone **17** which, upon treatment with NaBH_4_, produced epoxypyran **18** (Scheme 8). Epoxide **18** was obtained as a single diastereomer and should be a promising substrate for subsequent nucleophilic addition reactions leading to another series of enantiopure aminopyrans.

**Scheme 9:**
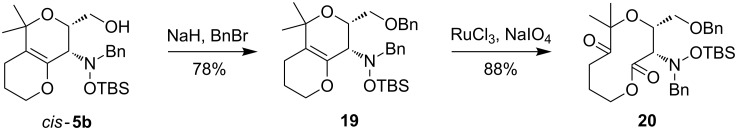
Oxidative cleavage of dihydropyran **19** to lactone **20**.

An additional option for the use of the enol ether moiety is oxidative cleavage [21]. Thus, benzyl protection of compound *cis*-**5b** followed by treatment of the resulting **19** with RuCl_3_ and NaIO_4_ gave the ten-membered ring lactone **20** by selective cleavage of the internal double bond (Scheme 9).

**Scheme 10:**
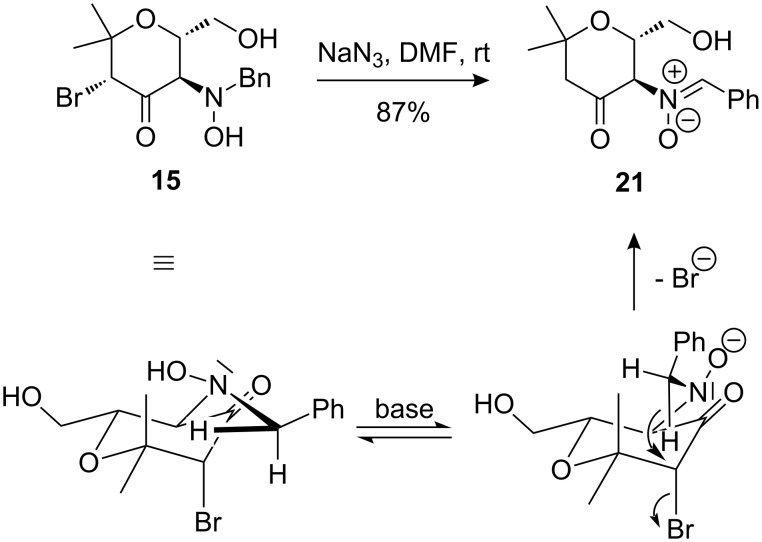
Transformation of α-bromoketone **15** into nitrone **21** by an internal redox reaction.

With the objective of preparing a diaminopyran, we attempted to substitute the bromide atom in compound **15** with the azido group. Surprisingly, instead of the desired α-azido ketone, the formation of nitrone **21** was observed, which apparently involves an unusual internal redox reaction (Scheme 10). Sodium azide presumably acts as base to initiate the reaction by deprotonation of the hydroxylamine moiety. A hydride shift from the benzylic position to the 3-position of the pyran ring produces the nitrone moiety of **21** and simultaneously displaces the axially positioned bromo substituent by an intramolecular substitution. This mechanistic hypothesis was supported by the fact that heating **15** in DMF in the absence of NaN_3_ did not lead to conversion of the substrate.

**Scheme 11:**
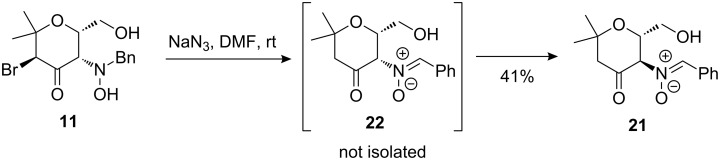
Transformation of α-bromoketone **11** into nitrone **21**.

When the diastereomeric α-bromoketone **11** was reacted with sodium azide, the identical nitrone **21** was obtained in moderate yield (Scheme 11). We presume that an initially formed nitrone **22** probably undergoes base induced epimerization to generate the thermodynamically more stable *trans*-substituted nitrone **21**. As shown below, the nitrone moiety of **21** can be regarded as a masked hydroxylamine, however, it should also be very useful for further diversification of our pyran derivatives, e.g., by 1,3-dipolar cycloaddition leading to isoxazole derivatives [22] or, by addition of nucleophiles to this electrophilic unit [9].

**Scheme 12:**
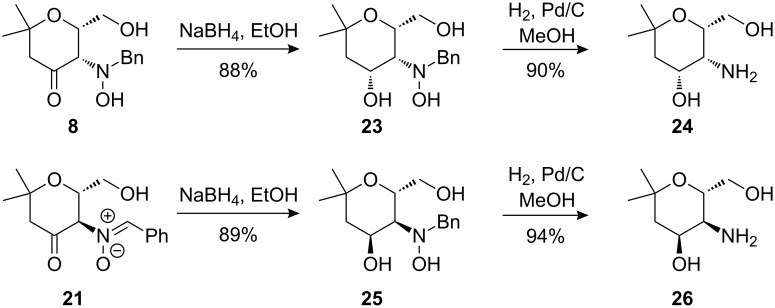
Synthesis of diastereomeric aminopyrans **24** and **26**.

Compounds **8** and **21** proved to be excellent precursors for the stereoselective preparation of enantiopure aminopyrans with high structural similarities to carbohydrates. Reduction of the carbonyl group of **8** by NaBH_4_ followed by hydrogenolysis of the *N*,*O*- and *N*-benzyl-bonds furnished aminopyran **24** (Scheme 12). Similarly, nitrone **21** was smoothly converted to the diastereomeric aminopyran **26** by analogous reductive transformations.

**Scheme 13:**
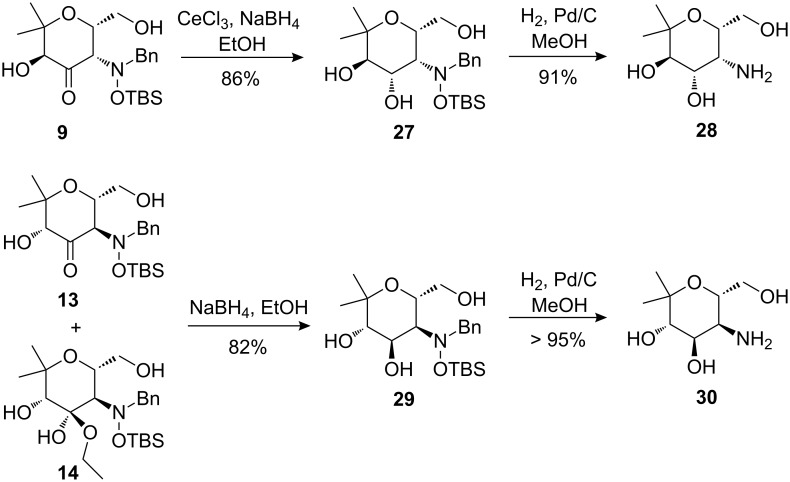
Synthesis of diastereomeric aminopyrans **28** and **30**.

α-Hydroxyketones **9** and **13** were used as precursors for the preparation of compounds with an additional hydroxyl group, such as aminopyrans **28** and **30** (Scheme 13). Reduction of **9** with NaBH_4_ in the presence of CeCl_3_ and subsequent hydrogenolysis furnished compound **28** in excellent yield. The presence of CeCl_3_ during the reduction of the carbonyl group proved to be essential to achieve excellent diastereoselectivity. The mixture of compounds **13** and **14**, which was formed during dihydroxylation of dihydropyran *cis*-**5a** (Scheme 6), was also treated with NaBH_4_ to afford the diastereomeric compound **29** [23]. A final hydrogenation step gave aminopyran **30** in high overall yield. Aminopyrans **24**, **26**, **28** and **30** can be regarded as mimetics of differently configured (2-deoxy) 4-amino sugars [24–26].

**Scheme 14:**
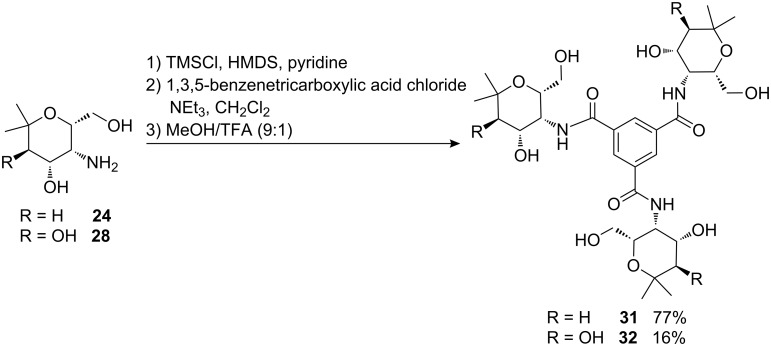
Synthesis of triamides **31** and **32**.

Aminopyrans **24** and **28** have already been linked via amide bonds to the thiol shell of gold nanoparticles (particle diameter 6 nm). After sulfation of the hydroxyl groups the multivalent conjugates obtained displayed strong binding to P- and L-selectins, thus demonstrating that compounds such as **24** and **28** are of interest for the development of new anti-inflammatory agents [27–28]. Inspired by these first findings, we decided to prepare also smaller conjugates for evaluation as selectin ligands. Thus, the free hydroxyl groups of aminopyrans **24** and **28** were temporarily protected as TMS ethers in order to achieve smooth reaction with 1,3,5-benzenetricarboxylic acid chloride (Scheme 14) [29]. After successful conversion to the triamide, the TMS-groups were removed by treatment with trifluoroacetic acid to give the desired compounds **31** and **32**. The low yield of **32** was due to difficulties encountered in the purification of the compound which was carried out by crystallization.

## Conclusion

We have presented an approach to novel 3,6-dihydro-2*H*-pyrans that can be transformed into a series of new carbohydrate mimetics and into other enantiopure heterocycles. The stereodivergent addition of lithiated enol ethers to carbohydrate-derived nitrones produced 1,3-dioxolanyl-substituted hydroxylamine derivatives as suitable substrates for Lewis-acid induced cyclizations furnishing mono-, di-, or spirocyclic dihydropyrans. Subsequent reactions such as bromination, acidic hydrolysis or dihydroxylation provided differently substituted and configured pyranone derivatives as precursors for the stereoselective synthesis of aminopyran derivatives that can be regarded as carbohydrate mimetics. Trimeric versions of these carbohydrate mimetics were constructed via their attachment to a tricarboxylic acid core to produce novel polyhydroxylated compounds that will be evaluated as potential selectin binders at a future date.

## Experimental

### Addition of lithiated enol ethers to glyceraldehyde-derived nitrones without precomplexation of the nitrone

The respective enol ether (1.5 equiv) was dissolved in THF (2 mL/mmol of enol ether) and cooled to −78 °C. *t*BuLi (1.6 M in pentane, 1.5 equiv) was added and the reaction mixture stirred for 1 h during which time it was allowed to warm to 0 °C. After further stirring for 1 h at this temperature, it was cooled once more to −78 °C. A solution of the respective nitrone (1 equiv) in THF (0.7 mL/mmol nitrone) was added dropwise over a 15 min period. The mixture was stirred at this temperature for 1 h and the reaction quenched by the addition of H_2_O. After the mixture reached room temperature it was extracted three times with Et_2_O. The combined organic phases were dried (Na_2_SO_4_) and the solvent was removed in vacuo. The crude product (1 equiv) was dissolved in CH_2_Cl_2_ (2.5 mL/mmol), and 2,6-lutidine (2 equiv) and TBSOTf (1.5 equiv) were added slowly at 0 °C. The mixture was stirred at room temperature for 30 min and then the reaction was quenched by the addition of a sat. NH_4_Cl solution. The layers were separated and the aqueous phase was extracted three times with CH_2_Cl_2_. The combined organic phases were dried (Na_2_SO_4_) and the solvent was removed in vacuo. Purification by column chromatography (silica gel, hexane/EtOAc = 20:1) yielded the products as colourless oils.

### Addition of lithiated enol ethers to glyceraldehyde-derived nitrones precomplexated by Et_2_AlCl

The respective enol ether (5 equiv) was dissolved in THF (2 mL/mmol of enol ether) and cooled to −78 °C. *t*BuLi (1.6 M in pentane, 5 equiv) was added and the reaction mixture stirred for 1 h during which time it was allowed to warm to 0 °C. After further stirring for 3 h at this temperature, it was cooled once more to −78 °C. In a separate flask, a solution of nitrone **2** (1 equiv) in THF (2.5 mL) was treated with Et_2_AlCl (1 M in hexane, 1 equiv) for 5 min. The prepared solution was added dropwise to the solution of the lithiated enol ether over a 15 min period. The mixture was stirred at this temperature for a further 15 min and the reaction quenched by addition of 2 M NaOH solution. After the mixture reached room temperature it was extracted three times with Et_2_O. The combined organic phases were dried (Na_2_SO_4_) and the solvent was removed in vacuo. The crude product (1 equiv) was dissolved in CH_2_Cl_2_ (2.5 mL/mmol), and 2,6-lutidine (2 equiv) and TBSOTf (1.5 equiv) were added slowly at 0 °C. The mixture was stirred at room temperature for 30 min and then the reaction was quenched by the addition of a sat. NH_4_Cl solution. The layers were separated and the aqueous phase was extracted three times with CH_2_Cl_2_. The combined organic phases were dried (Na_2_SO_4_) and the solvent was removed in vacuo. Purification by column chromatography (silica gel, hexane/EtOAc = 20:1) yielded the products as colourless oils.

### Lewis acid-induced cyclization of 1,3-dioxolanyl-substituted enol ethers

To a solution of the respective 1,3-dioxolanyl-substituted enol ether (1 equiv) in CH_2_Cl_2_ (6 mL/mmol) at −30 °C, was added TMSOTf (2 equiv) and the resulting solution stirred until it slowly reached room temperature (6 h). The reaction was quenched by the addition of water. After separation of the layers, the aqueous phase was extracted three times with CH_2_Cl_2_. The combined organic phases were dried (Na_2_SO_4_) and the solvent was removed in vacuo. Purification by column chromatography (silica gel, hexane/EtOAc = 6:1) yielded the products as colourless oils.

## Supporting Information

File 1 Experimental procedures, characterization data, ^1^H NMR and ^13^C NMR spectra of synthesized compounds.
