# Supplementary material for: Addition of lithiated enol ethers to nitrones and subsequent Lewis acid induced cyclizations to enantiopure 3,6-dihydro-2H-pyrans – an approach to carbohydrate mimetics
Source: Beilstein J Org Chem. 2010 Jul 9;6:75. doi: 10.3762/bjoc.6.75 (PMC2919261; doi:10.3762/bjoc.6.75)

Supporting Information

for

Addition of lithiated enol ethers to nitrones and subsequent Lewis acid induced cyclizations to enantiopure 3,6-dihydro-2*H*-pyrans – An approach to carbohydrate mimetics

Fabian Pfrengle and Hans-Ulrich Reissig*

Freie Universität Berlin, Institut für Chemie und Biochemie, Takustrasse 3, D-14195 Berlin, Germany

Email: Hans-Ulrich Reissig - hreissig@chemie.fu-berlin.de

* Corresponding author

Experimental procedures, characterization data, ^{1}H NMR and ^{13}C NMR spectra of synthesized compounds

General methods:

Reactions were, in general, performed under argon in flame-dried flasks, and the components were added by syringe. Methanol was purchased in p. a. quality and stored under argon over molecular sieves (4 Å). Tetrahydrofuran and dichloromethane were obtained from the solvent purification system MB-SPS-800 (M. Braun). Products were purified by flash chromatography on silica gel (230–400 mesh, Merck). Unless otherwise stated, yields refer to analytically pure samples. ^{1}H NMR [$CHCl_3$ (δ = 7.26 ppm), TMS (δ = 0.00 ppm), CD_3OD (δ = 3.31 ppm), DMF-d_7 (δ = 8.02 ppm) or D_2O (δ = 4.79 ppm) as internal standards] and ^{13}C NMR spectra [$CDCl_3$ (δ = 77.0 ppm), DMF-d_7 (δ = 162.6 ppm) or CD_3OD (δ = 49.0 ppm) as internal standards] were recorded on Bruker AC 250, ECP 400, AC 500, AVIII 700, or Joel Eclipse 500 instruments in $CDCl_3$, CD_3OD, DMF-d_7 or D_2O solution. Integrals are in accord with assignments; coupling constants are given in Hz. IR spectra were measured with an FT-IR spectrometer Nicolet 5 SXC or with a Nexus FT-IR equipped with a Nicolet Smart DuraSamplIR ATR. MS and HRMS analyses were performed on Finnigan Varian Ionspec QFT-7 (ESI-FT-ICR) and Agilent ESI-TOF 6210 (4 µL/min, 1 bar, 4000 V) instruments. Elemental analyses were obtained with "Elemental-Analyzers" (Perkin–Elmer or Carlo Erba). Melting points were measured with a Reichert apparatus (Thermovar) and are uncorrected. Optical rotations ($[\alpha]_D$) were determined with Perkin–Elmer 241 polarimeter at the temperatures given. Commercially available chemicals were used without further purification unless otherwise stated.

Experimental procedures and characterization data:

Due to hindered rotation of the bulky -*N*(OTBS)Bn moiety some signals in the ^{1}H NMR or ^{13}C NMR spectra of the compounds containing this group are broadened or could not be detected.

***N*-Benzyl-*O*-(*tert*-butyldimethylsilyl)-*N*-((*S*)-1-((*S*)-2,2-dimethyl-1,3-dioxolan-4-yl)-2-ethoxyallyl)hydroxylamine (*syn*-4a)**

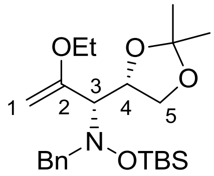

Ethylvinylether (445 µL, 4.62 mmol) was dissolved in THF (8 mL) and cooled to −78 °C. *t*BuLi (1.6 M in pentane, 2.89 mL, 4.62 mmol) was added and the reaction mixture stirred for 1 h during which time it was allowed to warm to 0 °C. After further stirring for 1 h at this temperature, it was cooled once more to −78 °C. A solution of nitrone **2a** (725 mg, 3.08 mmol) in THF (2 mL) was added dropwise over a period of 15 min. The mixture was stirred at this temperature for 1 h and the reaction quenched by the addition of H_2O. After the mixture reached room temperature, it was extracted three times with Et_2O. The combined organic phases were dried (Na_2SO_4) and the solvent was removed in vacuo. The crude product (844 mg) was dissolved in CH_2Cl_2 (7 mL), and 2,6-lutidine (641 µL, 5.50 mmol) and TBSOTf (946 µL, 4.13 mmol) were added slowly at 0 °C. The mixture was stirred at room temperature for 30 min and then the reaction was quenched by the addition of a sat. NH_4Cl solution. The layers were separated and the aqueous phase was extracted three times with CH_2Cl_2. The combined organic phases were dried (Na_2SO_4) and the solvent was removed in vacuo. Purification by column chromatography (silica gel, hexane/EtOAc = 20:1) yielded *syn*-**4a** (800 mg, 61%) and *anti*-**4a** (115 mg, 9%) as colorless oils. *syn*-**4a**: $[\alpha]_D^{22}$ = −27.2 (c = 0.20, $CHCl_3$). ^1H NMR (500 MHz, $CDCl_3$): δ = 0.10 (s, 6 H, $SiMe_2$), 0.88 (s, 9 H, *t*Bu), 1.28 (t, *J* = 7.0 Hz, 3 H, Et), 1.33, 1.36 (2 s, 3 H each, Me), 3.29 (s_{br}, 1 H, 3-H), 3.57 (t, *J* = 7.9 Hz, 1 H, 5-H), 3.66, 3.72 (2 td, *J* = 7.0, 9.4 Hz, 1 H each, Et), 3.85 (s_{br}, 1 H, NCH_2), 3.99 (dd, *J* = 6.8, 7.9 Hz, 1 H, 5-H), 4.03 (d, *J* = 1.6 Hz, 1 H, 1-H), 4.12 (d, *J* = 13.2 Hz, 1 H, NCH_2), 4.16 (d, *J* = 1.6 Hz, 1 H, 1-H), 4.40 (td, *J* = 6.8, 7.9 Hz, 1 H, 4-H), 7.20-7.42 (m, 5 H, Ph) ppm. ^{13}C NMR (176 MHz, $CDCl_3$): δ = −5.2, −4.8 (2 q, $SiMe_2$), 14.6 (q, Et), 17.9, 26.2 (s, q, *t*Bu), 25.6, 26.7 (2 q, Me), 60.5 (t, NCH_2), 62.2 (t, Et), 67.6 (t, C-5), 71.5 (d, C-3), 74.1 (d, C-4), 87.4 (t, C-1), 109.2 (s, C-2'), 126.9, 127.9, 129.8, 138.0 (3 d, s, Ph), 157.3 (s, C-2) ppm. IR (film): 3100–2850 cm^{-1} (=C-H, C-H). ESI-TOF: *m/z* calc. for $[M + Na]^+$ 444.2541, found 444.2546. Anal. calc. for $C_{23}H_{39}NO_4Si$ (421.7): C 65.52, H 9.32, N 3.32, found: C 65.39, H 9.37, N 3.36.

_N_-Benzyl-_O_-(_tert_-butyldimethylsilyl)-_N_-((_R_)-1-((_S_)-2,2-dimethyl-1,3-dioxolan-4-yl)-2-ethoxyallyl)hydroxylamine (_anti_-4a)

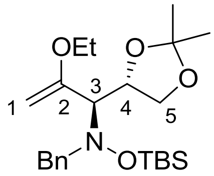

Ethylvinylether (409 µL, 4.25 mmol) was dissolved in THF (8 mL) and cooled to −78 °C. *t*BuLi (1.6 M in pentane, 2.66 mL, 4.25 mmol) was added and the reaction mixture stirred for 1 h during which time it was allowed to warm to 0 °C. After further stirring for 3 h at this temperature, it was once more cooled to -78 °C. A solution of nitrone **2a** (200 mg, 0.850 mmol) in THF (2 mL) was treated with Et_2AlCl (1 M in hexane, 850 µL, 0.850 mmol) for 5 min. The prepared solution was added dropwise over a period of 15 min. The mixture was then stirred at this temperature for a further 15 min and the reaction quenched by the addition of 2 M NaOH solution. After the mixture reached room temperature it was extracted three times with Et_2O. The combined organic phases were dried (Na_2SO_4) and the solvent was removed in vacuo. The crude product (250 mg) was dissolved in CH_2Cl_2 (3 mL), and 2,6-lutidine (180 µL, 1.22 mmol) and TBSOTf (268 µL, 1.63 mmol) were added slowly at 0 °C. The mixture was stirred at room temperature for 30 min and then the reaction was quenched by the addition of sat. NH_4Cl solution. The phases were separated and the aqueous phase was extracted three times with CH_2Cl_2. The combined organic phases were dried (Na_2SO_4) and the solvent was removed in vacuo. Purification by column chromatography (silica gel, hexane/EtOAc = 20:1) yielded *syn*-**4a** (14 mg, 4%) and *anti*-**4a** (171 mg, 48%) as colorless oils. *anti*-**4a**: $[\alpha]_D^{22}$ = +26.7 (c = 0.22, $CHCl_3$). ^1H NMR (500 MHz, $CDCl_3$): δ = −0.40 (s_{br}, 3 H, $SiMe_2$), -0.01 (s, 3 H, $SiMe_2$), 0.85 (s, 9 H, *t*Bu), 1.34 (m_c, 3 H, Et), 1.35 (s, 6 H, Me), 3.31 (s_{br}, 1 H, 3-H), 3.80–3.90 (m, 4 H, NCH_2, OCH_2, 3-H), 4.01 (m_c, 1 H, 5-H), 4.11 (dd, *J* = 5.8, 8.4 Hz, 1 H, 5-H), 4.16 (d, *J* = 1.9 Hz, 1 H, 1-H), 4.31 (d, *J* = 1.9 Hz, 1 H, 1-H), 4.42 (td, *J* = 5.8, 9.9 Hz, 1 H, 4-H), 7.20–7.34 (m, 5 H, Ph) ppm. ^{13}C NMR (126 MHz, $CDCl_3$): δ = −4.8, −4.6 (2 q, $SiMe_2$), 14.6 (q, Et), 17.8, 26.1 (s, q, *t*Bu), 25.7, 26.9 (2 q, Me), 60.8 (t, NCH_2), 62.4 (t, Et), 67.9 (t, C-5), 74.0 (d, C-4), 88.1 (t, C-1), 108.2 (s, C-2’), 127.2, 128.0, 130.2, 138.1 (3 d, s, Ph), 157.3 (s, C-2) ppm. IR (film): 3120–2840 cm^{-1} (=C-H, C-H). ESI-TOF: *m/z* calc. for $[M + Na]^+$ 444.2541, found 444.2523. Anal. calc. for $C_{23}H_{39}NO_4Si$ (421.7): C 65.52, H 9.32, N 3.32, found: C 64.93, H 8.60, N 3.52.

_N_-Benzyl-_O_-(_tert_-butyldimethylsilyl)-_N_-[(_S_)-(3,4-dihydro-2_H_-pyran-6-yl)((_S_)-2,2-dimethyl-1,3-dioxolan-4-yl)methyl]hydroxylamine (_syn_-4b)

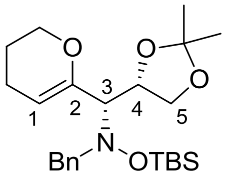

3,4-Dihydropyran (444 µL, 5.37 mmol) was dissolved in THF (5 mL) and cooled to −78 °C. *t*BuLi (1.6 M in pentane, 3.04 mL, 4.86 mmol) was added and the reaction mixture was stirred for 1 h during which time it was allowed to warm to 0 °C. After further stirring for 1 h at this temperature the mixture was once more cooled to -78 °C. A solution of nitrone **2a** (840 mg, 3.58 mmol) in THF (2 mL) was added dropwise over a period of 15 min. The mixture was then stirred at this temperature for 1 h and the reaction quenched by the addition of H_2O. After the mixture reached rt it was extracted 3 times with Et_2O. The combined organic phases were dried (Na_2SO_4) and the solvent was removed in vacuo. The crude product (980 mg) was dissolved in CH_2Cl_2 (8 mL), and 2,6-lutidine (1.07 mL, 9.18 mmol) and TBSOTf (1.40 mL, 6.11 mmol) were added slowly at 0 °C. The mixture was stirred at room temperature for 30 min and then the reaction was quenched by the addition of sat. NH_4Cl solution. The phases were separated and the aqueous phase was extracted three times with CH_2Cl_2. The combined organic phases were dried (Na_2SO_4) and the solvent was removed in vacuo. Purification by column chromatography (silica gel, hexane/EtOAc = 20:1) yielded *syn*-**4b** (682 mg, 44%) and *anti*-**4b** (276 mg, 18%) as colorless oils. *syn*-**4b**: $[\alpha]_D^{22}$ = −39.5 (c = 2.3, $CHCl_3$). ^{1}H NMR (500 MHz, $CDCl_3$): δ = −0.08 (s_{br}, 3 H, $SiMe_2$), 0.09 (s, 3 H, $SiMe_2$), 0.87 (s, 9 H, *t*Bu), 1.32, 1.35 (2 s, 3 H each, Me), 1.72–1.82 (m, 2 H, DHP), 2.02–2.10 (m, 2 H, DHP), 3.17 (s_{br}, 1 H, 3-H), 3.62 (dd, *J* = 6.9, 7.9 Hz, 1 H, 5-H), 3.84-3.95 (m, 3 H, DHP, NCH_2), 4.00 (dd, *J* = 6.9, 7.9 Hz, 1 H, 5-H), 4.09 (d, *J* = 13.1 Hz, 1 H, NCH_2), 4.36 (td, *J* = 6.9, 8.9 Hz, 1 H, 4-H), 4.69 (m_c, 1 H, 1-H), 7.16–7.41 (m, 5 H, Ph) ppm. ^{13}C NMR (126 MHz, $CDCl_3$): δ = −5.2, −4.8 (2 q, $SiMe_2$), 17.8, 26.1 (s, q, *t*Bu), 20.2, 22.3 (2 t, DHP), 25.6, 26.7 (2 q, Me), 60.6 (t, NCH_2), 65.3 (t, DHP), 67.6 (t, C-5), 74.2 (d, C-4), 102.8 (d, C-1), 109.1 (s, C-2'), 126.8, 127.9, 129.8 (3 d, Ph), 149.4 (s, C-2) ppm. IR (film): 3090–2820 cm^{-1} (=C-H, C-H). ESI-TOF: *m/z* calc. for $[M + Na]^+$ 456.2541, found 456.2538. Anal. calc. for $C_{24}H_{39}NO_4Si$ (433.7): C 66.47, H 9.06, N 3.23; found: C 66.51, H 9.06, N 3.31.

N-Benzyl-O-(*tert*-butyldimethylsilyl)-N-[(R)-(3,4-dihydro-2H-pyran-6-yl)((S)-2,2-dimethyl-1,3-dioxolan-4-yl)methyl]hydroxyl-amine (*anti*-4b)

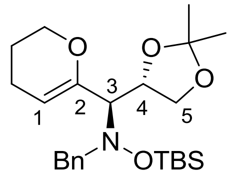

2,3-Dihydropyran (2.91 mL, 31.8 mmol) was dissolved in THF (30 mL) and cooled to −78 °C. *t*BuLi (1.6 M in pentane, 19.9 mL, 31.8 mmol) was added and the reaction mixture was stirred for 1 h during which time it was allowed to warm to 0 °C. After further stirring for 3 h at this temperature, it was once more cooled to −78 °C. A solution of nitrone **2a** (1.50 g, 6.36 mmol) in THF (10 mL) was treated with Et_2AlCl (1 M in hexane, 6.36 μL, 6.36 mmol) for 5 min. The prepared solution was added dropwise over a period of 15 min. The mixture was then stirred at this temperature for a further 15 min and the reaction was quenched by addition of 2M NaOH solution. After the mixture reached room temperature it was extracted 3 times with Et_2O. The combined organic phases were dried ($MgSO_4$) and the solvent was removed in vacuo. The crude product (2.15 g) was dissolved in CH_2Cl_2 (30 mL), and 2,6-lutidine (1.57 mL, 13.5 mmol) and TBSOTf (2.33 mL, 10.1 mmol) were added slowly at 0 °C. The mixture was stirred at room temperature for 30 min and the reaction quenched by the addition of a sat. NH_4Cl solution. The phases were separated and the aqueous phase was extracted three times with CH_2Cl_2. The combined organic phases were dried ($MgSO_4$) and the solvent was removed in vacuo. Purification by column chromatography (silica gel, hexane/EtOAc = 20:1) yielded *syn*-**4b** (215 mg, 8%) and *anti*-**4b** (1.54 g, 56%) as colorless oils. *anti*-**4b**: $[\alpha]_D^{22}$ = +39.1 (c = 0.80, $CHCl_3$). ^{1}H NMR (500 MHz, $CDCl_3$): δ = −0.37 (s_{br}, 3 H, $SiMe_2$), −0.03 (s, 3 H, $SiMe_2$), 0.84 (s, 9 H, *t*Bu), 1.33, 1.35 (2 s, 3 H each, Me), 1.81–1.94 (m, 2 H, DHP), 2.06–2.21 (m, 2 H, DHP), 3.18 (s_{br}, 1 H, 3-H), 3.81 (s_{br}, 1 H, NCH_2), 3.85 (d, J = 11.5 Hz, 1 H, NCH_2), 3.95–4.11 (m, 4 H, DHP, 5-H), 4.36 (dt, J = 5.7, 9.4 Hz, 1 H, 4-H), 4.77 (t, J = 3.6 Hz, 1 H, 1-H), 7.20–7.32 (m, 5 H, Ph) ppm. ^{13}C NMR (126 MHz, $CDCl_3$): δ = −4.8, −4.5 (2 q, $SiMe_2$), 17.8, 26.1 (s, q, *t*Bu), 20.4, 22.5 (2 t, DHP), 25.9, 27.0 (2 q, Me), 61.0 (t, NCH_2), 65.4 (t, DHP), 67.9 (t, C-5), 73.6 (d, C-4), 103.2 (d, C-1), 108.8 (s, C-2’), 127.2, 128.0, 130.2, 138.1 (3 d, s, Ph), 148.5 (s, C-2) ppm. IR (film): 3090–2800 cm^{-1} (=C-H, C-H). ESI-TOF: *m/z* calc. for $[M + Na]^+$ 456.2541, found 456.2523. Anal. calc. for $C_{24}H_{39}NO_4Si$ (433.7): C 66.47, H 9.06, N 3.23; found: C 66.12, H 8.90, N 3.32.

***N*-Benzyl-*O*-(*tert*-butyldimethylsilyl)-*N*-[(*S*)-(4,5-dihydrofuran-2-yl)((*S*)-2,2-dimethyl-1,3-dioxolan-4-yl)methyl]hydroxylamine (*syn*-4c)**

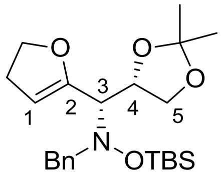

2,3-Dihydrofuran (726 µL, 9.60 mmol) was dissolved in THF (10 mL) and cooled to −78 °C. *t*BuLi (1.6 M in pentane, 6.00 mL, 9.60 mmol) was added and the reaction mixture was stirred for 1 h during which time it was allowed to warm to 0 °C. After further stirring for 1 h at this temperature, it was once more cooled to −78 °C. A solution of nitrone **2a** (1.50 g, 6.40 mmol) in THF (4 mL) was added dropwise over a period of 15 min. The mixture then was stirred at this temperature for 1 h and the reaction quenched by the addition of H_2O. After the mixture reached room temperature it was extracted three times with Et_2O. The combined organic phases were dried ($MgSO_4$) and the solvent was removed in vacuo. The crude product (1.91 g) was dissolved in CH_2Cl_2 (15 mL), and 2,6-lutidine (1.46 mL, 12.5 mmol) and TBSOTf (2.15 mL, 9.39 mmol) were added slowly at 0 °C. The mixture was stirred at room temperature for 30 min and the reaction was then quenched by the addition of sat. NH_4Cl solution. The phases were separated and the aqueous phase was extracted three times with CH_2Cl_2. The combined organic phases were dried ($MgSO_4$) and the solvent was removed in vacuo. Purification by column chromatography (silica gel, hexane/EtOAc = 20:1) yielded *syn*-**4c** (1.57 g, 58%) and *anti*-**4c** (260 mg, 10%) as colorless oils. *syn*-**4c**: $[\alpha]_D^{22}$ = −63.7 (c = 0.19, $CHCl_3$). ^1H NMR (500 MHz, $CDCl_3$): δ = −0.30 (s_{br}, 3 H, $SiMe_2$), 0.10 (s, 3 H, $SiMe_2$), 0.88 (s, 9 H, *t*Bu), 1.28, 1.33 (2 s, 3 H each, Me), 2.65 (m_c, 2 H, DHF), 3.36 (s_{br}, 1 H, 3-H), 3.61 (t, *J* = 7.5 Hz, 1 H, 5-H), 3.86, 4.06 (2 m_c, 1 H each, NCH_2), 3.98 (m_c, 1 H, 5-H), 4.22–4.36 (m, 3 H, DHF, 4-H), 4.89 (t, *J* = 2.1 Hz, 1 H, 1-H), 7.15−7.48 (m, 5 H, Ph) ppm. ^{13}C NMR (126 MHz, $CDCl_3$): δ = −4.8 (q, $SiMe_2$), 17.8, 26.1 (s, q, *t*Bu), 25.5, 26.7 (2 q, Me), 30.0 (t, DHF), 61.1 (t, NCH_2), 67.5 (t, C-5), 69.0 (t, DHF), 74.2 (d, C-4), 99.9 (d, C-1), 109.2 (s, C-2'), 127.0, 128.0, 129.7 (3 d, Ph) ppm. IR (film): 3100–2820 cm^{-1} (=C-H, C-H). ESI-TOF: *m/z* calc. for $C_{23}H_{37}NO_4Si$ $[M + Na]^+$ 442.2379, found 442.2380.

N-Benzyl-O-(tert-butyldimethylsilyl)-N-[(R)-(4,5-dihydrofuran-2-yl)((S)-2,2-dimethyl-1,3-dioxolan-4-yl)methyl]hydroxylamine (anti-4c)

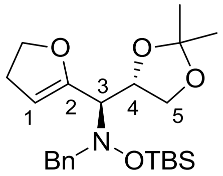

2,3-Dihydrofuran (802 µL, 10.6 mmol) was dissolved in THF (10 mL) and cooled to −78 °C. *t*BuLi (1.6 M in pentane, 6.63 mL, 10.6 mmol) was added and the reaction mixture was stirred for 1 h during which time it was allowed to warm to 0 °C. After further stirring for 3 h at this temperature, it was once more cooled to −78 °C. A solution of nitrone **2a** (500 mg, 2.12 mmol) in THF (4 mL) was treated with Et_2AlCl (1M in hexane, 2.12 µL, 2.12 mmol) for 5 min. The prepared solution was added dropwise over a period of 15 min. The mixture was then stirred at this temperature for another 15 min and the reaction quenched by the addition of 2M NaOH solution. After the mixture reached room temperature it was extracted 3 times with Et_2O. The combined organic phases were dried (Na_2SO_4) and the solvent was removed in vacuo. The crude product (680 mg) was dissolved in CH_2Cl_2 (10 mL), and 2,6-lutidine (519 µL, 4.45 mmol) and TBSOTf (268 µL, 3.35 mmol) were added slowly at 0 °C. The mixture was stirred at room temperature for 30 min and the reaction was then quenched by the addition of a sat. NH_4Cl solution. The phases were separated and the aqueous phase was extracted three times with CH_2Cl_2. The combined organic phases were dried (Na_2SO_4) and the solvent was removed in vacuo. Purification by column chromatography (silica gel, hexane/EtOAc = 20:1) yielded *syn*-**4c** (77 mg, 9%) and *anti*-**4c** (478 mg, 54%) as colorless oils. *anti*-**4c**: $[\alpha]_D^{22}$ = +46.8 (c = 0.41, $CHCl_3$). ^{1}H NMR (500 MHz, $CDCl_3$): δ = −0.24 (s_{br}, 3 H, $SiMe_2$), −0.19 (s, 3 H, $SiMe_2$), 0.85 (s, 9 H, *t*Bu), 1.31, 1.33 (2 s, 3 H each, Me), 2.73 (m_c, 2 H, DHF), 3.39 (s_{br}, 1 H, 3-H), 3.83 (m_c, 1 H, 5-H), 3.83, 3.94 (2 m_c, 1 H each, NCH_2), 4.06 (dd, *J* = 5.9, 8.5 Hz, 1 H, 5-H), 4.32–4.39 (m, 2 H, DHF, 4-H) 4.42 (q, *J* = 8.9 Hz, 1 H, DHF), 4.97 (t, *J* = 2.4 Hz, 1 H, 1-H), 7.19–7.37 (m, 5 H, Ph) ppm. ^{13}C NMR (126 MHz, $CDCl_3$): δ = −4.6 (q, $SiMe_2$), 17.8, 26.0 (s, q, *t*Bu), 25.7, 26.9 (2 q, Me), 30.2 (t, DHF), 67.9 (t, C-5), 69.3 (t, DHF), 74.3 (d, C-4), 109.1 (s, C-2'), 127.3, 128.1, 130.2, 137.8 (3 d, s, Ph) ppm. IR (film): 3100–2820 cm^{-1} (=C-H, C-H). ESI-TOF: *m/z* calc. for $C_{23}H_{37}NO_4Si$ $[M + Na]^+$ 442.2379, found 442.2377.

N-Benzyl-O-(tert-butyldimethylsilyl)-N-((S)-2-ethoxy-1-((S)-1,4-dioxaspiro[4.5]decan-2-yl)allyl)hydroxylamine (syn-4d)

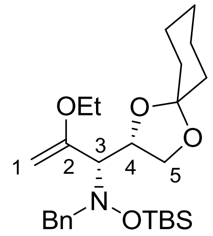

Ethylvinylether (289 µL, 3.00 mmol) was dissolved in THF (6 mL) and cooled to −78 °C. *t*BuLi (1.6 M in pentane, 1.88 mL, 3.00 mmol) was added and the reaction mixture was stirred for 1 h during which time it was allowed to warm to 0 °C. After further stirring for 1 h at this temperature, it was once more cooled to −78 °C. A solution of nitrone **2b** (550 mg, 2.00 mmol) in THF (2 mL) was added dropwise over a period of 15 min. The mixture was then stirred at this temperature for 1 h and the reaction quenched by the addition of H_2O. After the mixture reached room temperature it was extracted three times with Et_2O. The combined organic phases were dried (Na_2SO_4) and the solvent was removed in vacuo. The crude product (600 mg) was dissolved in CH_2Cl_2 (5 mL), and 2,6-lutidine (303 µL, 2.60 mmol) and TBSOTf (499 µL, 2.17 mmol) were added slowly at 0 °C. The mixture was stirred at room temperature for 30 min and was the reaction quenched by the addition of a sat. NH_4Cl solution. The phases were separated and the aqueous phase was extracted three times with CH_2Cl_2. The combined organic phases were dried (Na_2SO_4) and the solvent was removed in vacuo. Purification by column chromatography (silica gel, hexane/EtOAc = 20:1) yielded *syn*-**4d** (648 mg, 70%, dr > 95:5) as a colorless oil. $[\alpha]_D^{22}$ = −12.1 (c = 0.10, $CHCl_3$). ^{1}H NMR (500 MHz, $CDCl_3$): δ = 0.01 (s, 6 H, $SiMe_2$), 0.87 (s, 9 H, *t*Bu), 1.27 (t, *J* = 7.0 Hz, 3 H, Et), 1.30–1.37 (m, 2 H, Cy), 1.45–1.70 (m, 8 H, Cy), 3.25 (s_{br}, 1 H, NCH_2), 3.55 (m_c, 1 H, 5-H), 3.64–3.73 (m, 2 H, Et), 3.98 (m_c, 1 H, 5-H), 4.01 (m_c, 1 H, 1-H), 4.10–4.18 (m, 2 H, 1-H, NCH_2), 4.38 (td, *J* = 6.7, 9.1 Hz, 1 H, 4-H), 7.15–7.41 (m, 5 H, Ph) ppm. ^{13}C NMR (126 MHz, $CDCl_3$): δ = −5.1, −4.8 (2 q, $SiMe_2$), 14.5 (t, Et), 17.9, 26.2 (s, q, *t*Bu), 24.0, 25.7, 35.2, 36.5 (4 t, Cy), 60.4 (t, NCH_2), 62.2 (t, Et), 67.3 (t, C-5), 73.8 (d, C-4), 87.3 (t, C-1), 109.8 (s, C-2’), 126.9, 127.9, 129.6, 138.4 (3 d, s, Ph), 157.3 (s, C-2) ppm. IR (film): 3100–2820 cm^{-1} (=C-H, C-H). ESI-TOF: *m/z* calc. for $C_{36}H_{43}NO_4Si$ $[M + Na]^+$ 484.2854, found 484.2854.

N-Benzyl-O-(*tert*-butyldimethylsilyl)-N-((1S)-2-ethoxy-1-((4S,4'R)-2,2,2',2'-tetramethyl-4,4'-bi(1,3-dioxolan)-5-yl)allyl)hydroxylamine (*anti*-4e)

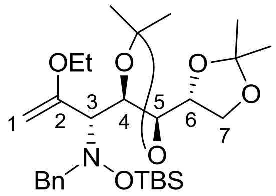

Ethyl vinyl ether (717 µL, 7.45 mmol) was dissolved in THF (12 mL) and cooled to −78 °C. *t*BuLi (1.6 M in pentane, 4.66 mL, 7.45 mmol) was added and the reaction mixture was stirred for 1 h during which time it was allowed to warm to 0 °C. After further stirring for 3 h at this temperature, it was once more cooled to −78 °C. A solution of nitrone **2c** (500 mg, 1.49 mmol) in THF (3 mL) was treated with Et_2AlCl (1 M in hexane, 1.49 mL, 1.49 mmol) for 5 min. The prepared solution was added dropwise over a period of 15 min. Then, the mixture was stirred at this temperature for another 15 min and the reaction quenched by the addition of 2 M NaOH solution. After the mixture reached room temperature it was extracted 3 times with Et_2O. The combined organic phases were dried (Na_2SO_4) and the solvent was removed in vacuo. The crude product (570 mg) was dissolved in CH_2Cl_2 (6 mL), and 2,6-lutidine (310 µL, 2.10 mmol) and TBSOTf (462 µL, 2.81 mmol) were added slowly at 0 °C. The mixture was stirred at room temperature for 30 min and the reaction quenched by the addition of sat. NH_4Cl solution. The phases were separated and the aqueous phase was extracted three times with CH_2Cl_2. The combined organic phases were dried (Na_2SO_4) and the solvent was removed in vacuo. Purification by column chromatography (silica gel, hexane/EtOAc = 20:1) yielded *anti*-**4e** (205 mg, 26%, d.r. > 95:5) as a colorless oil. $[\alpha]_D^{22}$ = −12.3 (c = 0.20, $CHCl_3$). ^{1}H NMR (500 MHz, $CDCl_3$): δ = −0.18 (s_{br}, 6 H, $SiMe_2$), 0.81 (s, 9 H, *t*Bu), 1.32 (t, J = 7.0 Hz, 3 H, Et), 1.35, 1.38, 1.39, 1.41 (4 s, 3 H each, Me), 3.73 (d, J = 6.0 Hz, 1 H, 3-H), 3.76–3.82 (m, 2 H, Et), 3.90–4.02, 4.10-4.20, 4.25–4.30, 4.48-4.53 (4 m, 9 H, 1-H, 4-H, 5-H, 6-H, 7-H, NCH_2), 7.19–7.38 (m, 5 H, Ph) ppm. Characteristic signals in ^{13}C NMR (126 MHz, $CDCl_3$): δ = −6.2, −4.8 (2 q, $SiMe_2$), 14.6 (q, Et), 17.8, 26.2 (s, q, *t*Bu), 25.5, 26.15, 26.16, 26.5 (4 q, Me), 60.4 (t, NCH_2), 62.4 (t, Et), 77.8 (t, C-7), 87.4 (t, C-1), 109.7, 109.8 (2 s, $\underline{C}Me_2$), 126.9, 127.7, 130.8, 139.0 (3 d, s, Ph), 157.0 (s, C-2) ppm. IR (ATR): 3100–2830 cm^{-1} (=C-H, C-H). ESI-TOF: *m/z* calc. for $C_{28}H_{48}NO_6Si$ [M + Na]$^+$ 544.3065, found 544.3074.

((2*S*,3*S*)-3-(Benzyl(*tert*-butyldimethylsiloxy)amino)-4-ethoxy-6,6-dimethyl-3,6-dihydro-2*H*-pyran-2-yl)methanol (*cis*-5a)

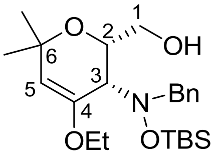

To a solution of *syn*-**4a** (135 mg, 0.321 mmol) in CH_2Cl_2 (2 mL) at −30 °C, was added TMSOTf (119 μL, 0.643 mmol), and the resulting solution stirred until it slowly reached room temperature (6 h). The reaction was then quenched by the addition of water. After separation of the layers, the aqueous phase was extracted three times with CH_2Cl_2. The combined organic phases were dried (Na_2SO_4) and the solvent was removed in vacuo. Purification by column chromatography (silica gel, hexane/EtOAc = 6:1) yielded *cis*-**5a** (106 mg, 79%) as a colorless oil. $[\alpha]_D^{22}$ = −118.6 (c = 0.25, $CHCl_3$). ^{1}H NMR (400 MHz, $CDCl_3$): δ = −0.05, 0.03 (2 s, 3 H each, $SiMe_2$), 0.79 (s, 9 H, *t*Bu), 1.24, 1.31 (2 s, 3 H each, Me), 1.37 (t, *J* = 7.0 Hz, 3 H, Et), 3.55 (dd, *J* = 2.1, 7.1 Hz, 1 H, 3-H), 3.75 (q, *J* = 7.0 Hz, 2 H, Et), 3.89–3.98 (m, 3 H, 2-H, OH), 4.11 (m_c, 2 H, 1-H), 4.73 (s, 1 H, 5-H), 7.21–7.26 (m, 5 H, Ph) ppm. ^{13}C NMR (101 MHz, $CDCl_3$): δ = −5.0 (q, $SiMe_3$), 14.9 (q, Et), 17.8, 26.1 (s, q, *t*Bu), 25.9, 30.3 (2 q, Me), 62.0 (t, C-1), 62.3 (t, Et), 64.2 (d, C-3), 73.0 (s, C-6), 73.4 (d, C-2), 106.7 (d, C-5), 127.2, 128.0, 130.6, 139.0 (3 d, s, Ph), 149.8 (s, C-4) ppm. IR (film): 3450 cm^{-1} (OH), 3090-2840 (=C-H, C-H), 1660 (C=C). ESI-TOF: *m/z* calc. for $[M + H]^+$ 422.2727, found 422.2753. Anal. calc. for $C_{23}H_{39}NO_4Si$ (421.7): C 65.52, H 9.32, N 3.32, found: C 65.12, H 9.54, N 3.37.

((2*S*,3*R*)-3-[Benzyl(*tert*-butyldimethylsiloxy)amino]-4-ethoxy-6,6-dimethyl-3,6-dihydro-2*H*-pyran-2-yl)methanol (*trans*-5a)

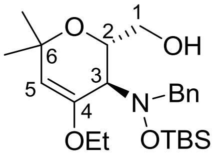

To a solution of *anti*-**4a** (1.83 g, 4.34 mmol) in CH_2Cl_2 (25 mL) at −30 °C was added TMSOTf (1.60 μL, 8.27 mmol), and the resulting solution was stirred until it slowly reached room temperature (6 h). The reaction was then quenched by the addition of water. After separation of the layers, the aqueous phase was extracted three times with CH_2Cl_2. The combined organic phases were dried ($MgSO_4$) and the solvent was removed in vacuo. Purification by column

chromatography (silica gel, hexane/EtOAc = 6:1) yielded *trans*-**5a** (1.53 g, 84%) as a colorless oil. $[\alpha]_D^{22}$ = +69.7 (c = 0.66, $CHCl_3$). ^{1}H NMR (500 MHz, $CDCl_3$): δ = −0.16, 0.01 (2 s, 3 H each, $SiMe_2$), 0.82 (s, 9 H, *t*Bu), 1.23, 1.29 (2 s, 3 H each, Me), 1.41 (t, *J* = 7.0 Hz, 3 H, Et), 2.81 (s_{br}, 1 H, OH), 3.36 (m_c, 1 H, 3-H), 3.53 (m_c, 1 H, 1-H), 3.65–3.78 (m, 2 H, Et), 3.82 (td, *J* = 5.5, 10.6 Hz, 1 H, 1-H), 3.95 (d, *J* = 12.5 Hz, 1 H, NCH_2), 4.00 (s_{br}, 1 H, 2-H), 4.34 (m_c, 1 H, NCH_2), 4.69 (d, *J* = 1.1 Hz, 1 H, 5-H), 7.18–7.35 (m, 5 H, Ph) ppm. ^{13}C NMR (126 MHz, $CDCl_3$): δ = −4.9, −4.7 (2 q, $SiMe_2$), 14.9 (q, Et), 17.8, 26.0 (s, q, *t*Bu), 26.7, 31.2 (2 q, Me), 61.0 (d, C-3), 62.2 (t, Et), 64.9 (t, C-1), 70.2 (d, C-2), 72.5 (s, C-6), 105.7 (d, C-5), 127.3, 128.1, 130.3, 137.9 (3 d, s, Ph), 151.5 (s, C-4) ppm. IR (film): 3400 cm^{-1} (OH), 3090-2840 (=C-H, C-H), 1660 (C=C). ESI-TOF: *m/z* calc. for $[M + H]^+$ 422.2727, found 422.2744. Anal. calc. for $C_{23}H_{39}NO_4Si$ (421.7): C 65.52, H 9.32, N 3.32, found: C 65.32, H 9.28, N 3.39.

((7*S*,8*S*)-8-[Benzyl(*tert*-butyldimethylsiloxy)amino]-5,5-dimethyl-2,3,4,5,7,8-hexahydropyrano[4,3-*b*]pyran-7-yl)methanol (*cis*-5b)

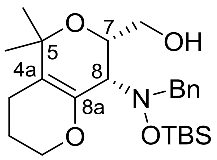

To a solution of *syn*-**4b** (590 mg, 1.36 mmol) in CH_2Cl_2 (6 mL) at −30 °C was added TMSOTf (501 μL, 2.72 mmol), and the resulting solution was stirred until it slowly reached room temperature (6 h). The reaction was then quenched by the addition of water. The resulting mixture was extracted three times with CH_2Cl_2. The combined organic phases were dried (Na_2SO_4) and the solvent was removed in vacuo. Purification by column chromatography (silica gel, hexane/EtOAc = 6:1) yielded *cis*-**5b** (434 mg, 74%) as a colorless oil. $[\alpha]_D^{22}$ = −130.2 (c = 0.58, $CHCl_3$). ^{1}H NMR (500 MHz, $CDCl_3$): δ = −0.37 (s_{br}, 6 H, $SiMe_2$), 0.79 (s, 9 H, *t*Bu), 1.26, 1.30 (2 s, 3 H each, Me), 1.81-1.92 (m, 3 H, DHP), 2.06 (m_c, 1 H, DHP), 3.49 (s_{br}, 1 H, 8-H), 3.64 (s_{br}, 1 H, 7-CH_2), 3.87–3.97 (m, 3 H, 7-H, 7-CH_2, NCH_2), 4.01–4.18 (m, 3 H, DHP, NCH_2), 7.16–7.32 (m, 5 H, Ph) ppm. ^{13}C NMR (101 MHz, $CDCl_3$): δ = −5.3 (q, $SiMe_2$), 17.7, 26.0 (s, q, *t*Bu), 20.6, 22.6 (2 t, DHP), 23.5, 27.7 (2 q, Me), 64.0 (t, 7-CH_2), 65.2 (t, DHP), 72.6 (d, C-7), 75.2 (s, C-5), 115.4 (s, C-4a), 127.0, 127.9, 130.6, 139.2 (3 d, s, Ph), 143.2 (s, C-8a) ppm. IR (film): 3450 cm^{-1} (OH), 3100–2850 (=C-H, C-H), 1660 (C=C). ESI-TOF: *m/z* calc. for $C_{24}H_{39}NO_4Si$ $[M + H]^+$ 434.2721, found 434.2724.

((7*S*,8*R*)-8-(Benzyl(*tert*-butyldimethylsiloxy)amino)-5,5-dimethyl-2,3,4,5,6,7,8-hexahydropyrano[4,3-*b*]pyran-7-yl)methanol (*trans*-5b)

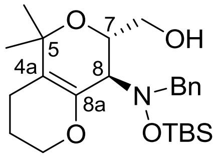

To a solution of *anti*-**4b** (270 mg, 0.622 mmol) in CH_2Cl_2 (3 mL) at −30 °C was added TMSOTf (228 μL, 1.25 mmol), and the resulting solution was stirred until it slowly reached room temperature (6 h). The reaction was then quenched by the addition of water. The resulting mixture was extracted three times with CH_2Cl_2. The combined organic phases were dried (Na_2SO_4) and the solvent was removed in vacuo. Purification by column chromatography (silica gel, hexane/EtOAc = 6:1) yielded *trans*-**5b** (221 mg, 82%) as a colorless oil. $[\alpha]_D^{22}$ = +39.8 (c = 0.49, $CHCl_3$). ^{1}H NMR (500 MHz, $CDCl_3$): δ = −0.24, 0.05 (2 s_{br}, 3 H each, $SiMe_2$), 0.85 (s, 9 H, *t*Bu), 1.23, 1.29 (2 s, 3 H each, Me), 1.79–1.93 (m, 3 H, DHP), 2.02–2.10 (m, 1 H, DHP), 2.84 (s_{br}, 1 H, OH), 3.36 (d, *J* = 7.4 Hz, 1 H, 8-H), 3.51 (td, *J* = 5.2, 10.6 Hz, 1 H, 7-CH_2), 3.81 (m_c, 1 H, 7-CH_2), 3.86 (m_c, 1 H, OCH_2), 3.92 (d, *J* = 12.6 Hz, 1 H, NCH_2), 4.01 (m_c, 1 H, 7-H), 4.14 (m_c, 1 H, OCH_2), 4.33 (d, *J* = 12.6 Hz, 1 H, NCH_2), 7.16–7.34 (m, 5 H, Ph) ppm. ^{13}C NMR (101 MHz, $CDCl_3$): δ = −5.0, −4.6 (2 q, $SiMe_2$), 17.8, 26.1 (s, q, *t*Bu), 20.9, 22.8 (2 t, DHP), 24.4, 28.7 (2 q, Me), 60.6 (t, NCH_2), 65.0 (t, OCH_2), 65.1 (t, 7-CH_2), 69.4 (d, C-7), 74.5 (s, C-5), 114.3 (s, C-4a), 127.2, 128.1, 130.3, 138.2 (3 d, s, Ph), 144.3 (s, C-8a) ppm. IR (film): 3450 cm^{-1} (OH), 3100-2850 (=C-H, C-H), 1670 (C=C). ESI-TOF: *m/z* calc. for $[M + H]^+$ 434.2721, found 434.2719.

((6*S*,7*S*)-7-[Benzyl(*tert*-butyldimethylsiloxy)amino]-4,4-dimethyl-3,4,6,7-tetrahydro-2*H*-furo[3,2-*c*]pyran-6-yl)methanol (*cis*-5c)

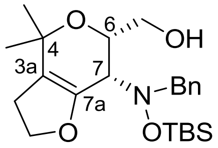

To a solution of *syn*-**4c** (1.52 g, 3.62 mmol) in CH_2Cl_2 (15 mL) at −30 °C was added TMSOTf (1.32 mL, 7.24 mmol), and the resulting solution was stirred until it slowly reached room temperature (6 h). The reaction was then quenched by the addition of water. The resulting

mixture was extracted three times with CH_2Cl_2. The combined organic phases were dried ($MgSO_4$) and the solvent was removed in vacuo. Purification by column chromatography (silica gel, hexane/EtOAc = 6:1) yielded *cis*-**5c** (1.29 g, 85%) as a colorless oil. $[\alpha]_D^{22}$ = −130.0 (c = 0.61, $CHCl_3$). ^{1}H NMR (500 MHz, $CDCl_3$): δ = −0.51, −0.25 (2 s_{br}, 3 H each, $SiMe_2$), 0.80 (s, 9 H, *t*Bu), 1.26, 1.32 (2 s, 3 H each, Me), 2.53–2.66 (m, 2 H, DHF), 3.28 (s_{br}, 1 H, OH), 3.64 (s_{br}, 1 H, 6-H), 3.84 (s_{br}, 1 H, NCH_2), 3.91 (s_{br}, 1 H, 6-CH_2), 3.96 (m_c, 1 H, 4-H), 4.10 (d, *J* = 13.4 Hz, 1 H, NCH_2), 4.13 (m_c, 1 H, 6-CH_2), 4.37–4.45 (m, 2 H, DHF), 7.17–7.32 (m, 5 H, Ph) ppm. ^{13}C NMR (101 MHz, $CDCl_3$): δ = −5.3, −5.1 (2 q, $SiMe_2$), 17.7, 26.0 (s, q, *t*Bu), 23.1, 27.8 (2 q, Me), 29.6 (t, DHF), 60.8 (t, NCH_2), 62.6 (d, C-7), 63.8 (t, 6-CH_2), 69.1 (t, DHF), 73.1 (d, C-6), 74.1 (s, C-4), 117.4 (s, C-3a), 127.1, 127.9, 130.6, 138.6 (3 d, s, Ph), 146.8 (s, C-7a) ppm. IR (film): 3460 cm^{-1} (OH), 3100–2800 (=C-H, C-H), 1690 (C=C). ESI-TOF: *m/z* calc. for $C_{23}H_{37}NO_4Si$ $[M + Na]^+$ 442.2379, found 442.2377.

((6*S*,7*R*)-7-(Benzyl(*tert*-butyldimethylsiloxy)amino)-4,4-dimethyl-3,4,6,7-tetrahydro-2*H*-furo[3,2-*c*]pyran-6-yl)methanol (*trans*-5c)

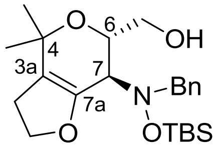

To a solution of *anti*-**4c** (209 mg, 0.498 mmol) in CH_2Cl_2 (2 mL) at −30 °C was added TMSOTf (182 μL, 0.996 mmol), and the resulting solution was stirred until it slowly reached room temperature (6 h). The reaction was then quenched by the addition of water. After separation of the phases the aqueous phase was extracted three times with CH_2Cl_2. The combined organic phases were dried (Na_2SO_4) and the solvent was removed in vacuo. Purification by column chromatography (silica gel, hexane/EtOAc = 6:1) yielded *trans*-**5c** (115 mg, 55%) as a colorless oil. $[\alpha]_D^{22}$ = +56.5 (c = 0.93, $CHCl_3$). ^{1}H NMR (500 MHz, $CDCl_3$): δ = −0.13, 0.03 (2 s_{br}, 3 H each, $SiMe_2$), 0.86 (s, 9 H, *t*Bu), 1.24, 1.31 (2 s, 3 H each, Me), 2.43–2.51 (m, 1 H, DHF), 2.57–2.67 (m, 1 H, DHF), 2.67 (s_{br}, 1 H, OH), 3.41 (s_{br}, 1 H, 7-H), 3.54 (s_{br}, 1 H, 6-CH_2), 3.84 (m_c, 1 H, 6-CH_2), 3.90–3.99 (m, 2 H, 6-H, NCH_2), 4.22–4.29 (m, 1 H, NCH_2), 4.34 (q, *J* = 9.0 Hz, 1 H, DHF), 4.44 (ddd, *J* = 6.3, 9.0, 10.5 Hz, 1 H, DHF), 7.18–7.39 (m, 5 H, Ph) ppm. ^{13}C NMR (101 MHz, $CDCl_3$): δ = −4.9, −4.6 (2 q, $SiMe_2$), 17.7, 26.0 (s, q, *t*Bu), 24.5, 28.1 (2 q, Me), 29.7 (t, DHF), 60.9 (t, NCH_2), 64.4 (t, 6-CH_2), 69.4 (t, DHF), 70.3 (d, C-6), 73.4 (s, C-4), 116.1 (s, C-3a),

127.4, 128.0, 130.0, 137.7 (3 d, s, Ph), 148.2 (s, C-7a) ppm. IR (film): 3460 cm^{-1} (OH), 3100–2800 (=C-H, C-H), 1700 (C=C). ESI-TOF: *m/z* calc. for $C_{23}H_{37}NO_4Si$ $[M + Na]^+$ 442.2379, found 442.2357.

((2*S*,3*S*)-3-(Benzyl(*tert*-butyldimethylsiloxy)amino)-4-ethoxy-1-oxaspiro[5.5]undec-4-en-2-yl)methanol (*cis*-5d)

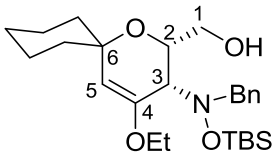

To a solution of *syn*-**4d** (420 mg, 0.910 mmol) in CH_2Cl_2 (6 mL) at −30 °C was added TMSOTf (331 μL, 1.82 mmol), and the resulting solution was stirred until it slowly reached room temperature (6 h). The reaction was then quenched by the addition of water. After separation of the phases the aqueous phase was extracted three times with CH_2Cl_2. The combined organic phases were dried (Na_2SO_4) and the solvent was removed in vacuo. Purification by column chromatography (silica gel, hexane/EtOAc = 10:1) yielded *cis*-**5d** (380 mg, 90%) as colorless crystals. $[\alpha]_D^{22}$ = −108.0 (c = 0.14, $CHCl_3$). ^{1}H NMR (500 MHz, $CDCl_3$): δ = −0.51, −0.27 (2 s_{br}, 3 H each, $SiMe_2$), 0.80 (s, 9 H, *t*Bu), 1.21–1.81 (m, 10 H, Cy), 1.37 (t, *J* = 7.0 Hz, 3 H, Et), 3.45 (s_{br}, 1 H, OH), 3.55 (s_{br}, 1 H, 3-H), 3.75 (q, *J* = 7.0 Hz, 2 H, Et), 3.82–3.96 (m, 3 H, 2-H, 1-H, NCH_2), 4.09–4.21 (m, 2 H, 1-H, NCH_2), 4.77 (s, 1 H, 5-H), 7.19–7.34 (m, 5 H, Ph) ppm. ^{13}C NMR (126 MHz, $CDCl_3$): δ = −5.4, −5.2 (2 q, $SiMe_2$), 14.7 (q, Et), 17.6, 25.9 (s, q, *t*Bu), 21.7, 22.1, 25.4, 33.3, 38.7 (5 t, Cy), 60.6 (t, NCH_2), 62.0 (t, Et), 64.0 (t, C-1), 65.3 (d, C-3), 72.3 (s, C-6), 73.5 (d, C-2), 106.0 (d, C-5), 126.9, 127.7, 130.6, 139.0 (3 d, s, Ph), 150.1 (s, C-4) ppm. IR (film): 3450 cm^{-1} (OH), 3090–2840 (=C-H, C-H), 1660 (C=C). ESI-TOF: *m/z* calc. for $[M + Na]^+$ 484.2848, found 484.2868. Anal. calc. for $C_{26}H_{43}NO_4Si$ (461.7): C 67.64, H 9.39, N 3.03, found: C 67.29, H 9.66, N 2.90.

***N*-Benzyl-*O*-(*tert*-butyldimethylsilyl)-*N*-((*S*)-((*S*)-2,2-dimethyl-1,3-dioxolan-4-yl)(furan-2-yl)methyl)hydroxylamine (*syn*-4f)**

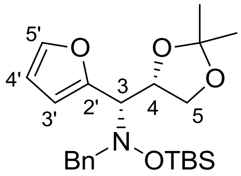

Furan (279 μL, 3.84 mmol) was dissolved in THF (4 mL) and cooled to −78 °C. *n*BuLi (2.5 M in hexanes, 1.54 mL, 3.84 mmol) was added and the reaction mixture stirred for 1 h during which time it was allowed to warm to 0 °C. After further stirring for 1 h at this temperature, it was once more cooled to −78 °C. A solution of nitrone **2a** (300 mg, 1.28 mmol) in THF (1 mL) was added dropwise over a period of 15 min. Then, the mixture was stirred at this temperature for 2 h and the reaction quenched by the addition of a sat. NH_4Cl solution. After the mixture reached room temperature it was extracted three times with Et_2O. The combined organic phases were dried ($MgSO_4$) and the solvent was removed in vacuo. The crude product (340 mg) was dissolved in CH_2Cl_2 (5 mL), and 2,6-lutidine (190 μL, 1.63 mmol) and TBSOTf (281 μL, 1.22 mmol) were added slowly at 0 °C. The mixture was stirred at room temperature for 30 min and then the reaction was quenched by the addition of sat. NH_4Cl solution. The phases were separated and the aqueous phase was extracted three times with CH_2Cl_2. The combined organic phases were dried ($MgSO_4$) and the solvent was removed in vacuo. Purification by column chromatography (silica gel, hexane/EtOAc = 20:1) yielded *syn*-**4f** (294 mg, 55%) as a colorless oil. $[\alpha]_D^{22}$ = −71.8 (c = 0.60, $CHCl_3$). ^{1}H NMR (500 MHz, $CDCl_3$): δ = 0.15, 0.26 (2 s_{br}, 3 H each, $SiMe_2$), 0.93 (s, 9 H, *t*Bu), 1.26, 1.36 (2 s, 3 H each, Me), 3.43 (m_c, 1 H, 3-H), 3.59 (m_c, 1 H, 5-H), 3.75–3.90 (m, 2 H, NCH_2), 4.04 (m_c, 1 H, 5-H), 4.55 (m_c, 1 H, 4-H), 6.32 (d, *J* = 3.1 Hz, 1 H, 3'-H), 6.38 (dd, *J* = 1.8, 3.1 Hz, 1 H, 4'-H), 7.19–7.41 (m, 6 H, Ph, 5'-H) ppm. ^{13}C NMR (126 MHz, $CDCl_3$): δ = −4.8 (q, $SiMe_2$), 17.8, 26.1 (s, q, *t*Bu), 25.6, 26.6 (2 q, Me), 61.2 (t, NCH_2), 67.3 (t, C-5), 74.7 (d, C-4), 109.4 (s, $\underline{C}Me_2$), 128.2 (d, C-4'), 129.4 (d, C-3'), 127.2, 128.2, 129.4 (3 d, Ph), 141.8 (d, C-5') ppm. IR (film): 3100–2830 cm^{-1} (=C-H, C-H). ESI-TOF: *m/z* calc. for $C_{23}H_{35}NO_4Si$ $[M + Na]^+$ 440.2228, found 440.2223.

(5*S*,6*S*)-5-[Benzyl(hydroxy)amino]-6-(hydroxymethyl)-2,2-dimethyldihydro-2*H*-pyran-4(3*H*)one (8)

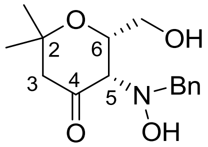

To compound *cis*-**5a** (350 mg, 0.830 mmol), was added satd. methanolic HCl (20 mL) and the resulting mixture stirred for 12 h at room temperature. Then the solvent was removed in vacuo and satd. $NaHCO_3$ solution and CH_2Cl_2 added to the residue. The layers were separated and the aqueous phase was extracted twice with CH_2Cl_2. The combined organic layers were dried (Na_2SO_4) and the solvent was removed in vacuo to yield **8** (180 mg, 78%) as a brownish oil. The product was used in the next step (preparation of **23**) without further purification.

(3*S*,5*S*,6*S*)-5-[Benzyl(*tert*-butyldimethylsilyl)amino]-3-hydroxy-6-(hydroxymethyl)-2,2-dimethyldihydro-2*H*-pyran-4(3*H*)one (9)

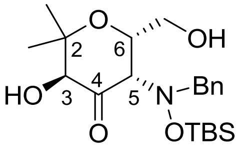

To a solution of *cis*-**5a** (260 mg, 0.616 mmol) in acetone (3 mL), were added H_2O (300 μL), $K_2OsO_4{\cdot}2H_2O$ (16 mg, 0.043 mmol) and *N*-methylmorpholine-*N*-oxide (50 wt % in H_2O, 170 μL, 0.840 mmol). The reaction mixture was stirred for 3 d at room temperature. Solid Na_2SO_3 (106 mg, 0.840 mmol) was then added and the mixture was stirred for 1 h. The mixture was filtered through a pad of celite, dried (Na_2SO_4) and the solvent removed in vacuo. Purification by column chromatography (silica gel, hexane/EtOAc = 3:1) yielded **9** (193 mg, 76%) as a colorless oil. $[\alpha]_D^{22}$ = −6.0 (c = 0.52, $CHCl_3$). ^{1}H NMR (500 MHz, $CDCl_3$): δ = 0.30 (s_{br}, 6 H, $SiMe_2$), 0.90 (s, 9 H, *t*Bu), 1.06, 1.42 (2 s, 3 H each, Me), 1.94 (s_{br}, 1 H, OH), 3.36 (s_{br}, 1 H), 3.63 (s_{br}, 2 H), 3.79 (d, *J* = 12.1 Hz, 1 H), 3.85–4.01 (m, 2 H), 4.12 (s_{br}, 1 H), 4.40 (d, *J* = 3.3 Hz, 1 H), 7.24–7.41 (m, 5 H, Ph) ppm. ^{13}C NMR (126 MHz, $CDCl_3$): δ = −4.7 (q, $SiMe_2$), 18.0, 28.2 (2 q, Me), 18.6, 25.9 (s, q, *t*Bu), 62.3, 63.6, 73.0, 83.6, 128.2, 128.8, 130.0 ppm. IR (film): 3480 cm^{-1} (OH), 3090–2820 (=C-H, C-H). ESI-TOF: *m/z* calc. for $C_{21}H_{35}NO_5Si$ $[M + Na]^+$432.2177, found 432.2184.

(3*S*,5*S*,6*S*)-5-[Benzyl(*tert*-butyldimethylsiloxy)amino]-3-bromo-6-(hydroxymethyl)-2,2-dimethyldihydro-2*H*-pyran-4(3*H*)one (10) and (3*S*,5*S*,6*S*)-5-[Benzyl(hydroxy)amino]3-bromo-6-(hydroxymethyl)-3,3-dimethyldihydro-2*H*-pyran-4(3*H*)one (11)

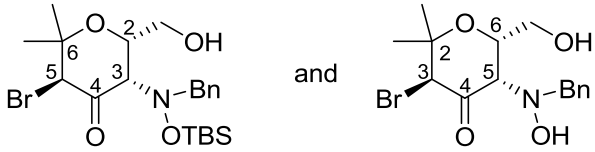

To a solution of *cis*-**5a** (100 mg, 0.237 mmol) in MeCN (2 mL), were added H_2O (200 μL) and *N*-bromosuccinimide (42 mg, 0.237 mmol). After stirring the reaction mixture for 15 min, a further quantity of H_2O was added and the mixture extracted three times with CH_2Cl_2. The combined organic phases were dried (Na_2SO_4) and the solvent was removed in vacuo. Purification by column chromatography (silica gel, hexane/EtOAc = 10:1 to 2:1) yielded **10** (28 mg, 25%) and **11** (44 mg, 52%) as colorless oils. **10**: $[\alpha]_D^{22}$ = −3.0 (c = 1.91, $CHCl_3$). ^{1}H NMR (500 MHz, $CDCl_3$): δ = 0.29, 0.36 (2 s_{br}, 3 H each, $SiMe_2$), 0.92 (s, 9 H, *t*Bu), 1.27, 1.52 (2 s, 3 H each, Me), 3.33 (s_{br}, 1 H, 3-H), 3.59 (d, *J* = 8.9 Hz, 1 H, NCH_2), 3.84 (d, *J* = 11.6 Hz, 1 H, 2-CH_2), 3.88–3.98 (m, 2 H, 2-H, NCH_2), 4.03 (d, *J* = 11.6 Hz, 1 H, 2-CH_2), 5.03 (s, 1 H, 5-H), 7.22–7.40 (m, 5 H, Ph) ppm. ^{13}C NMR (101 MHz, $CHCl_3$): δ = −4.7, −3.6 (2 q, $SiMe_2$), 17.9, 25.9 (s, q, *t*Bu), 20.7, 29.6 (2 q, Me), 62.1 (t, NCH_2), 63.2 (t, 2-CH_2), 66.5 (d, C-3), 66.6 (d, C-5), 72.6 (d, C-2), 79.6 (s, C-6), 128.3, 128.9, 130.0, 135.5 (3 d, s, Ph), 197.0 (s, C-4) ppm. IR (film): 3420 cm^{-1} (OH), 3090–2820 (=C-H, C-H), 1720 (C=O). ESI-TOF: *m/z* calc. for $C_{24}H_{38}BrNO_4Si$ $[M + Na]^+$ 534.1640, found 534.1655. **11**: $[\alpha]_D^{22}$ = +64.4 (c = 0.73, $CHCl_3$). ^{1}H NMR (500 MHz, $CHCl_3$): δ = 1.30, 1.54 (2 s, 3 H each, Me), 3.44 (d, *J* = 4.4 Hz, 1 H, 5-H), 3.79 (d, *J* = 13.2 Hz, 1 H, NCH_2), 3.79 (dd, *J* = 4.4, 12.1 Hz, 1 H, 6-CH_2), 3.85 (d, *J* = 13.2 Hz, 1 H, NCH_2), 3.93 (dd, *J* = 4.4, 12.1 Hz, 1 H, 6-CH_2), 4.01 (q, *J* = 4.4 Hz, 1 H, 6-H), 5.06 (s, 1 H, 3-H), 6.15 (s_{br}, 1 H, OH), 7.22–7.38 (m, 5 H, Ph) ppm. ^{13}C NMR (101 MHz, $CHCl_3$): δ = 20.5, 29.6 (2 q, Me), 61.7 (t, NCH_2), 62.6 (t, 6-CH_2), 65.3 (d, C-3), 71.8 (d, C-5), 72.1 (d, C-6), 80.0 (s, C-2), 127.8, 128.5, 129.4, 136.2 (3 d, s, Ph), 196.9 (s, C-4) ppm. IR (film): 3410 cm^{-1} (OH), 3090–2820 (=C-H, C-H), 1710 (C=O). ESI-TOF: *m/z* calc. for $C_{15}H_{20}BrNO_4$ $[M + Na]^+$ 380.0462, found 380.0478.

(3*R*,5*R*,6*S*)-5-[Benzyl(*tert*-butyldimethylsiloxy)amino]3-hydroxy-6-(hydroxymethyl)-2,2-dimethyldihydro-2*H*-pyran-4(3*H*)one (13) and (3*R*,4*R*,5*R*,6*S*)-5-[Benzyl(*tert*-butyldimethylsiloxy)amino]4-ethoxy-6-(hydroxymethyl)-2,2-dimethyltetrahydro-2*H*-pyran-3,4-diol (14)

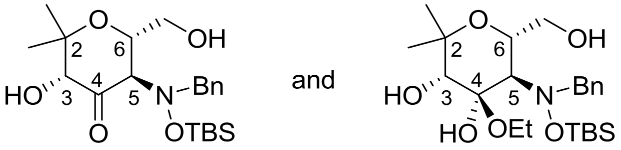

To a solution of *trans*-**5a** (165 mg, 0.391 mmol) in acetone (2 mL), were added H_2O (200 μL), $K_2OsO_4 \cdot 2H_2O$ (24 mg, 0.065 mmol) and *N*-methylmorpholine-*N*-oxide (50 wt % in H_2O, 167 μL, 0.825 mmol). The reaction mixture was stirred for 3 d at room temperature. Solid Na_2SO_3 (50 mg, 0.391 mmol) was then added and the mixture was stirred for 1 h. The mixture was filtered through a pad of celite, dried (Na_2SO_4) and the solvent removed in vacuo. Purification by column chromatography (silica gel, hexane/EtOAc = 3:1) gave starting material (50 mg) and a mixture of the desired α-hydroxyketone **13** and the hydroxylated hemiacetal **14** (108 mg, 1:1 ratio, 64%) as a colorless oil. The components of the mixture could not been separated and was directly used in the next step (preparation of **29**).

(3*R*,5*R*,6*S*)-5-[Benzyl(hydroxy)amino]3-bromo-6-(hydroxymethyl)-2,2-dimethyldihydro-2*H*-pyran-4(3*H*)one (15)

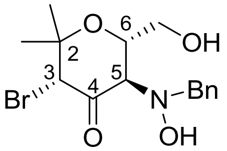

To a solution of *trans*-**5a** (500 mg, 1.19 mmol) in MeCN (8 mL), were added H_2O (800 μL) and *N*-bromosuccinimide (211 mg, 1.19 mmol). After stirring the reaction mixture for 15 min, a further quantity of H_2O was added and the mixture extracted three times with CH_2Cl_2. The combined organic phases were dried (Na_2SO_4) and the solvent was removed in vacuo. Purification by column chromatography (silica gel, hexane/EtOAc = 2:1) yielded **15** (298 mg, 70%) as colorless crystals. M.p. 109–111 °C. $[\alpha]_D^{22} = -144.5$ (c = 0.38, $CHCl_3$). ^1H NMR (700 MHz, $CDCl_3$): δ = 1.40, 1.42 (2 s, 3 H each, Me), 3.77 (dd, J = 3.6, 11.6 Hz, 1 H, 6-CH_2), 3.80 (dd, J = 3.5, 11.6 Hz, 1 H, 6-CH_2), 4.04 (s, 1 H, 3-H), 4.17 (m_c, 1 H, 6-H), 4.20 (d, J = 12.8 Hz, 1 H, NCH_2), 4.25 (d, J = 9.6 Hz, 1 H, 5-H), 4.55 (d, J = 12.8 Hz, 1 H, NCH_2), 5.63 (s_{br}, 1 H, OH), 7.26–7.37 (m, 5 H, Ph) ppm. ^{13}C NMR (176 MHz, $CDCl_3$): δ = 22.7, 28.0 (2 q, Me), 59.2 (d, C-

3), 62.5 (t, NCH_2), 63.6 (d, C-5), 63.7 (t, 6-CH_2), 73.6 (d, C-6), 74.8 (s, C-2), 127.8, 128.5, 129.1, 136.7 (3 d, s, Ph), 202.1 (s, C-4) ppm. IR (KBr): 3260 cm^{-1} (OH), 3090–2820 (=C-H, C-H), 1730 (C=O). ESI-TOF: *m/z* calc. for $C_{15}H_{20}BrNO_4$ $[M + Na]^+$ 380.0462, found 380.0453.

(3*R*,5*R*,6*S*)-5-(Benzyl(hydroxy)amino)-3-bromo-6-(hydroxymethyl)-3-(3-hydroxypropyl)-2,2-dimethyldihydro-2*H*-pyran-4(3*H*)one (16)

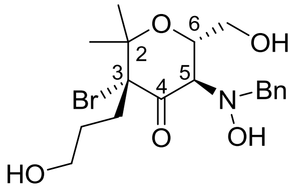

To a solution of *trans*-**5b** (1.15 g, 2.66 mmol) in MeCN (15 mL), were added H_2O (1.5 mL) and *N*-bromosuccinimide (472 mg, 2.66 mmol). After stirring the reaction mixture for 30 min, a further quantity of H_2O was added and the mixture extracted three times with CH_2Cl_2. The combined organic phases were dried (Na_2SO_4) and the solvent was removed in vacuo. Purification by column chromatography (silica gel, CH_2Cl_2/EtOAc = 1:1) yielded **16** (845 mg, 76%, d.r. > 92:8) as a colorless oil. $[\alpha]_D^{22}$ = −59.8 (c = 0.99, $CHCl_3$). ^1H NMR (500 MHz, $CHCl_3$): δ = 1.31, 1.46 (2 s, 3 H each, Me), 1.66–1.73 (m, 1 H, 1'-H), 1.73–1.81 (m, 1 H, 2'-H), 1.91-1.99 (m, 1 H, 2'-H), 2.05 (m_c, 1 H, 1'-H), 3.62–3.71 (m, 2 H, OCH_2), 3.77–3.84 (m, 2 H, 6-CH_2), 4.13–4.20 (m, 2 H, NCH_2, 6-H), 4.52–4.58 (m, 2 H, NCH_2, 5-H), 5.90 (s_{br}, 1 H, OH), 7.18–7.39 (m, 5 H, Ph) ppm. ^{13}C NMR (126 MHz, $CHCl_3$): δ = 21.5, 25.7 (2 q, Me), 29.9 (t, C-1'), 30.8 (t, C-2'), 62.4 (t, NCH_2), 62.5 (t, OCH_2), 63.8 (t, 6-CH_2), 64.5 (d, C-5), 73.6 (d, C-6), 79.1, 79.3 (2 s, C-2, C-3), 127.6, 128.4, 129.1, 137.0 (3 d, s, Ph), 203.7 (s, C-4) ppm. Characteristic signals of minor diastereomer: ^1H NMR (500 MHz, $CHCl_3$): 1.41, 1.63 (2 s, 3 H each, Me) ppm. IR (film): 3360 cm^{-1} (OH), 3090–2780 (=C-H, C-H), 1700 (C=O). ESI-TOF: *m/z* calc. for $C_{18}H_{26}BrNO_5$ $[M + Na]^+$ 438.0887, found 438.0883.

(2*S*,3*S*,5*S*)-3-(Benzyl(*tert*-butyldimethylsiloxy)amino)-5-bromo-2-(hydroxymethyl)-1-oxa-spiro[5.5]undecan-4-one (17)

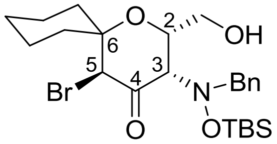

To a solution of *cis*-**5d** (330 mg, 0.715 mmol) in MeCN (6 mL) were added H_2O (600 μL) and *N*-bromosuccinimide (127 mg, 0.715 mmol). After stirring the reaction mixture for 10 min, a further quantity of H_2O was added and the mixture extracted three times with CH_2Cl_2. The combined organic phases were dried (Na_2SO_4) and the solvent was removed in vacuo. Purification by column chromatography (silica gel, hexane/EtOAc = 8:1) yielded **17** (230 mg, 63%) as a colorless oil. $[\alpha]_D^{22}$ = −10.4 (c = 1.30, $CHCl_3$). ^{1}H NMR (500 MHz, $CHCl_3$): δ = 0.28, 0.34 (2 s_{br}, 3 H each, $SiMe_2$), 0.90 (s, 9 H, *t*Bu), 1.12–1.31 (m, 2 H, Cy), 1.41–1.59 (m, 3 H, Cy), 1.59–1.78 (m, 4 H, Cy), 1.85 (s_{br}, 1 H, OH), 1.91–2.00 (m, 1 H, Cy), 3.34 (s_{br}, 1 H, 3-H), 3.61 (s_{br}, 1 H, NCH_2), 3.79 (s_{br}, 1 H, 2-H), 3.83 (d, *J* = 11.9 Hz, 1 H, 2-CH_2), 3.98–4.01 (m, 2 H, 2-CH_2, NCH_2), 4.96 (s, 1 H, 5-H), 7.22–7.39 (m, 5 H, Ph) ppm. ^{13}C NMR (126 MHz, $CHCl_3$): δ = −4.8, −3.6 (2 q, $SiMe_2$), 17.9, 25.9 (s, q, *t*Bu), 20.3, 21.4, 24.7, 25.6, 36.8 (5 t, Cy), 62.2 (t, NCH_2), 63.0 (t, 2-CH_2), 66.6 (d, C-3), 67.5 (d, C-5), 71.2 (d, C-2), 80.1 (s, C-6), 128.1, 128.8, 129.9, 135.5 (3 d, s, Ph), 196.9 (s, C-4) ppm. IR (film): 3700–3400 cm^{-1} (OH), 3090-2830 (=C-H, C-H), 1720 (C=O). ESI-TOF: *m/z* calc. for $C_{24}H_{38}BrNO_4Si$ $[M + Na]^+$ 534.1651, found 534.1655.

((1*S*,4*S*,5*S*,6*S*)-5-(Benzyl(*tert*-butyldimethylsiloxy)amino)-3,7-dioxa-spiro[bicyclo[4.1.0]heptane-2,1'-cyclohexane]-4-yl)methanol (18)

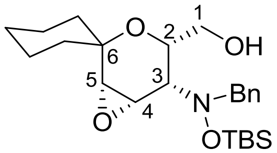

To a solution of **17** (195 mg, 0.380 mmol) in EtOH (3 mL), was added $NaBH_4$ (70 mg, 0.252 mmol) at 0 °C. The mixture was then stirred for 1 h at room temperature, the solvent removed in vacuo and CH_2Cl_2 and H_2O added to the residue. The layers were separated and the aqueous phase was extracted two times with CH_2Cl_2. The combined organic layers were dried (Na_2SO_4) and the solvent was removed in vacuo. Purification by recrystallization (hexane/Et_2O) yielded **18** (163 mg, 99%) as colorless crystals. M.p. 134–136 °C. $[\alpha]_D^{22}$ = +2.1 (c = 3.9, $CHCl_3$).

^{1}H NMR (500 MHz, $CHCl_3$): δ = −0.80, 0.00 (2 s_{br}, 3 H each, $SiMe_2$), 0.79 (s, 9 H, *t*Bu), 1.16–1.34 (m, 2 H, Cy), 1.44 (d, *J* = 13.3 Hz, 1 H, Cy), 1.49–1.56 (m, 2 H, Cy), 1.61–1.70 (m, 2 H, Cy), 1.86–2.06 (m, 3 H, Cy), 2.29 (s_{br}, 1 H, OH), 3.62–3.93 (m, 3 H), 4.07 (s_{br}, 1 H), 4.27–4.38 (m, 3 H), 4.45 (s_{br}, 1 H), 7.22–7.34 (m, 5 H, Ph) ppm. ^{13}C NMR (126 MHz, $CHCl_3$): δ = −4.8, −4.6 (2 q, $SiMe_2$), 17.6, 25.9 (s, q, *t*Bu), 20.5, 21.3, 24.8, 25.4, 36.5 (5 t, Cy), 63.3, 63.6, 70.2, 77.3, 99.9 (s, C-6), 127.9, 128.3, 130.5, 137.3 (3 d, s, Ph) ppm. IR (KBr): 3460 cm^{-1} (OH), 3100–2820 (=C-H, C-H). ESI-TOF: *m/z* calc. for $C_{24}H_{39}NO_4Si$ $[M + Na]^+$ 456.2541, found 456.2532.

N-Benzyl-N-((7S,8S)-7-(benzyloxymethyl)-5,5-dimethyl-2,3,4,5,7,8-hexahydropyrano[4,3-b]pyran-8-yl)-O-(tert-butyldimethylsilyl)hydroxylamine (19)

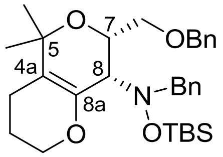

To a solution of *cis*-**5b** (600 mg, 1.38 mmol) in THF (13 mL), was added NaH (60% in paraffin oil, 84 mg, 2.16 mmol) at 0 °C. The reaction mixture was stirred for 1 h at room temperature, then cooled to 0 °C and BnBr (247 μL, 1.98 mmol) added. The mixture was stirred for 12 h at room temperature. Sat. NH_4Cl solution was added and the mixture extracted three times with CH_2Cl_2. The combined organic phases were dried ($MgSO_4$) and concentrated. Purification by column chromatography (silica gel, hexane/EtOAc = 15:1) yielded **19** (560 mg, 78%) as a colorless oil. $[\alpha]_D^{22}$ = −103.9 (c = 0.44, $CHCl_3$). ^{1}H NMR (500 MHz, $CDCl_3$): δ = 0.07, 0.08 (2 s, 3 H each, $SiMe_2$), 0.91 (s, 9 H, *t*Bu), 1.25, 1.26 (2 s, 3 H each, Me), 1.86–1.97 (m, 3 H, DHP), 2.04–2.13 (m, 1 H, DHP), 3.44 (s_{br}, 1 H, 8-H), 3.69 (dd, *J* = 3.8, 10.5 Hz, 1 H, 7-CH_2), 3.78–3.89 (m, 3 H, 7-H, 7-CH_2, NCH_2), 3.97 (m_c, 1 H, DHP), 4.11–4.17 (m, 2 H, DHP, NCH_2), 4.24 (d, *J* = 11.1 Hz, 1 H, $OC\underline{H}_2Ph$), 4.40 (d, *J* = 11.1 Hz, 1 H, $OC\underline{H}_2Ph$), 7.04–7.10 (m, 2 H, Ph), 7.15–7.42 (m, 8 H, Ph) ppm. ^{13}C NMR (126 MHz, $CDCl_3$): δ = −5.1, −4.9 (2 q, $SiMe_2$), 18.3, 26.0 (s, q, *t*Bu), 20.5, 22.7 (2 t, DHP), 23.4, 28.0 (2 q, Me), 61.1 (t, NCH_2), 61.6 (d, C-8), 64.1 (t, 7-CH_2), 65.1 (t, DHP), 73.4 (d, C-7), 74.8 (t, $O\underline{C}H_2Ph$), 75.6 (s, C-5), 115.3 (s, C-4a), 126.8, 127.3, 127.9, 128.0, 128.8, 129.9, 137.9, 139.4 (6 d, 2 s, Ph), 143.4 (s, C-8a) ppm. IR (film): 3100–2850 cm^{-1} (=C-H, C-H), 1670 (C=C). ESI-TOF: *m/z* calc. for $[M + H]^+$ 546.3005, found 546.2981. Anal. calc. for $C_{31}H_{45}NO_4Si$ (523.8): C 71.09, H 8.66, N 2.67, found: C 71.21, H 8.65, N 2.82.

(3*S*,4*S*)-3-[Benzyl(*tert*-butyldimethylsiloxy)amino]-4-(benzyloxymethyl)-6,6-dimethyl-1,5-dioxecane-2,7-dione (20)

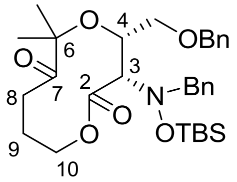

To a solution of compound **19** (100 mg, 0.191 mmol) in H_2O:CCl_4:MeCN (1.5:1:1, 1.4 mL), was added $RuCl_3$ (0.1 M in H_2O, 57 μL, 5.73 μmol) and $NaIO_4$ (168 mg, 0.783 mmol). The mixture was stirred at room temperature for 2 h and the reaction then quenched by the addition of satd. $Na_2S_2O_3$ solution. The resulting mixture was extracted three times with CH_2Cl_2. The combined organic phases were dried (Na_2SO_4) and concentrated to afford 94 mg of **20** (94 mg, 88%) as an analytically pure yellow oil. $[\alpha]_D^{22}$ = −43.8 (c = 0.96, $CHCl_3$). ^{1}H NMR (500 MHz, $CDCl_3$): δ = 0.10, 0.11 (2 s, 3 H each, $SiMe_2$), 0.92 (s, 9 H, *t*Bu), 1.31, 1.35 (2 s, 3 H each, Me), 1.82 (m_c, 1 H, 9-H), 2.01 (ddd, *J* = 1.3, 11.6, 16.7 Hz, 1 H, 8-H), 2.58 (m_c, 1 H, 9-H), 2.94 (ddd, *J* = 1.5, 11.6, 16.7 Hz, 1 H, 8-H), 3.72 (dd, *J* = 5.0, 9.4 Hz, 1 H, 4-CH_2), 3.81 (ddd, *J* = 2.0, 7.3, 10.9 Hz, 1 H, 10-H), 3.96 (m_c, 1 H, 4-H), 4.11 (t, *J* = 9.4 Hz, 1 H, 4-CH_2), 4.22 (d, *J* = 3.3 Hz, 1 H, 3-H), 4.29–4.40 (m, 3 H, OC$\underline{H}_2$Ph, NCH_2), 4.69 (d, *J* = 13.8 Hz, 1 H, NCH_2), 4.91 (dt, *J* = 6.0, 10.9 Hz, 1 H, 10-H), 6.98–7.49 (m, 10 H, Ph) ppm. ^{13}C NMR (101 MHz, $CDCl_3$): δ = −5.1 (q, $SiMe_2$), 18.4, 26.1 (s, q, *t*Bu), 21.7 (t, C-9), 21.8, 26.0 (2 q, Me), 31.4 (t, C-8), 60.0 (t, NCH_2), 61.0 (t, C-10), 62.7 (t, 4-CH_2), 66.7 (d, C-3), 75.7 (t, O$\underline{C}H_2$Ph), 75.8 (d, C-4), 80.9 (s, C-6), 127.2, 127.6, 128.1, 128.2, 128.7, 129.8, 137.2, 139.3 (6 d, 2 s, Ph), 169.9 (s, C-2), 209.3 (s, C-7) ppm. IR (film): 3100–2830 cm^{-1} (=C-H, C-H), 1750, 1710 (C=O). ESI-TOF: *m/z* calc. for $C_{31}H_{45}NO_6Si$ [M + Na]$^+$ 578.2903, found 578.2911.

(2*S*,3*R*,*Z*)-*N*-Benzylidene-2-(hydroxymethyl)-6,6-dimethyl-4-oxotetrahydropyran-3-amine oxide (21)

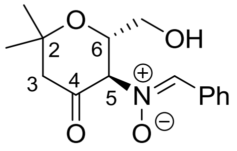

To a solution of **15** (110 mg, 0.307 mmol) in DMF (3 mL), was added NaN_3 (99 mg, 1.52 mmol) and the reaction mixture was stirred for 12 h at room temperature. H_2O was added and the resulting mixture extracted three times with ethyl acetate. The combined organic phases were

dried (Na_2SO_4) and the solvent was removed in vacuo. Purification by column chromatography (silica gel, CH_2Cl_2/EtOAc = 1:1) yielded **21** (74 mg, 87%) as colorless crystals. M.p. 156 °C. $[\alpha]_D^{22}$ = +73.8 (c = 0.13, $CHCl_3$). ^{1}H NMR (700 MHz, $CDCl_3$): δ = 1.39, 1.41 (2 s, 3 H each, Me), 2.50, 2.57 (AB system, J_{AB} = 14.5 Hz, 1 H each, 3-H), 2.57 (s_{br}, 1 H, OH), 3.70 (dd, *J* = 2.2, 12.3 Hz, 1 H, 6-CH_2), 3.94 (dd, *J* = 2.2, 12.3 Hz, 1 H, 6-CH_2), 4.79 (dt, J = 2.2, 9.7 Hz, 1 H, 6-H), 4.83 (d, *J* = 9.7 Hz, 1 H, 5-H), 7.39 (s, 1 H, N=C<u>H</u>Ph), 7.40–7.45 (m, 3 H, Ph), 8.23-8.26 (m, 2 H, Ph) ppm. ^{13}C NMR (176 MHz, $CDCl_3$): δ = 24.3, 30.3 (2 q, Me), 51.8 (t, C-3), 62.1 (t, 6-CH_2), 72.4 (d, C-6), 75.4 (s, C-2), 77.8 (d, C-5), 128.4, 128.9, 129.7, 131.0 (3 d, s, Ph), 138.2 (d, N=<u>C</u>HPh), 199.5 (s, C-4) ppm. IR (KBr): 3280 cm^{-1} (OH), 3090–2860 (=C-H, C-H), 1720 (C=O). ESI-TOF: *m/z* calc. for $C_{15}H_{19}NO_4$ $[M + Na]^+$ 300.1212, found 300.1256.

(4*R*,5*R*,6*S*)-5-[Benzyl(hydroxy)amino]-6-(hydroxymethyl)-2,2-dimethyltetrahydro-2*H*-pyran-4-ol (23)

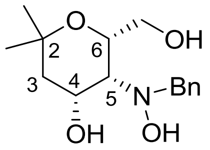

Crude ketone **8** (180 mg, 0.645 mmol) was dissolved in ethanol (1 mL) and cooled to 0 °C. $NaBH_4$ (36 mg, 0.966 mmol) was added and the mixture stirred for 1 h at 0 °C. The solvent was then removed in vacuo and CH_2Cl_2 and H_2O added to the residue. The layers were separated and the aqueous phase was extracted twice with CH_2Cl_2. The combined organic layers were dried (Na_2SO_4) and the solvent was removed in vacuo. Purification by recrystallization (hexane/EtOAc) yielded the product **23** (160 mg, 88%) as colorless crystals. M.p. 86–88 °C. $[\alpha]_D^{22}$ = +26.7 (c = 0.61, $CHCl_3$). ^{1}H NMR (500 MHz, $CDCl_3$): δ = 1.20, 1.25 (2 s, 3 H each, Me), 1.76 (dd, *J* = 5.6, 12.8 Hz, 1 H, 3-H), 1.96 (t, *J* = 12.8 Hz, 1 H, 3-H), 3.16 (dd, *J* = 2.9, 5.6 Hz, 1 H, 5-H), 3.70 (dt, *J* = 2.9, 5.1 Hz, 1 H, 6-H), 3.79 (dd, *J* = 5.1, 11.6 Hz, 1 H, 6-CH_2), 3.89 (dd, *J* = 5.1, 11.6 Hz, 1 H, 6-CH_2), 4.04 (dt, *J* = 5.6, 11.8 Hz, 1 H, 4-H), 4.24 (s, 2 H, NCH_2), 6.42 (s_{br}, 1 H, OH), 7.24–7.36 (m, 5 H, Ph) ppm. ^{13}C NMR (101 MHz, $CDCl_3$): δ = 23.1, 31.4 (2 q, Me), 42.3 (t, C-3), 63.2 (d, C-5), 63.7 (t, 6-CH_2), 64.0 (t, NCH_2), 67.9 (d, C-4), 71.9 (d, C-6), 73.4 (s, C-2), 127.5, 128.4, 129.3, 138.0 (3 d, s, Ph) ppm. IR (KBr): 3390–3200 cm^{-1} (OH), 3090–2840 (=C-H, C-H). ESI-TOF: *m/z* calc. for $C_{15}H_{23}NO_4$ $[M + H]^+$ 282.1700, found 282.1713. Anal. calc. for $C_{15}H_{23}NO_4$ (281.3): C 64.03, H 8.24, N 4.98, found: C 63.77, H 7.86, N 4.98.

(4*R*,5*R*,6*S*)-5-Amino-6-(hydroxymethyl)-2,2-dimethyltetrahydro-2*H*-pyran-4-ol (24)

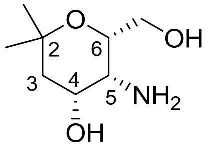

A suspension of palladium on charcoal (10% Pd, 50 mg) in MeOH (4 mL) was saturated with hydrogen for 1 h. After the addition of compound **23** (50 mg, 0.178 mmol) in MeOH (2 mL), hydrogen was bubbled through the mixture for a further 30 min. The reaction mixture was then stirred under an atmosphere of hydrogen for 24 h. Filtration through a short pad of celite and concentration of the solution yielded **24** (28 mg, 90%) as a colorless oil. $[\alpha]_D^{22}$ = +70.9 (c = 0.47, MeOH). ^{1}H NMR (500 MHz, CD_3OD): δ = 1.20, 1.25 (2 s, Me), 1.49 (t, *J* = 13.1 Hz, 1 H, 3-H), 1.70 (dd, *J* = 5.1, 13.1 Hz, 1 H, 3-H), 3.23 (d, *J* = 5.1 Hz, 1 H, 5-H), 3.62 (dd, *J* = 5.3 Hz, 11.5, 1 H, 6-CH_2), 3.89 (dd, *J* = 5.3, 11.5 Hz, 1 H, 6-CH_2), 3.76 (t, *J* = 5.3 Hz, 1 H, 6-H), 4.07 (td, 5.1, 13.1 Hz, 1 H, 4-H) ppm. ^{13}C NMR (126 MHz, CD_3OD): δ = 23.1, 31.3 (2 q, Me), 39.6 (t, C-3), 53.2 (d, C-5), 63.5 (t, 6-CH_2), 65.9 (d, C-4), 71.5 (d, C-6), 74.5 (s, C-2) ppm. IR (film): 3400–3100 cm^{-1} (OH), 2950-2840 (C-H). ESI-TOF: *m/z* calc. for $C_8H_{17}NO_3$ $[M + H]^+$ 176.1281, found 176.1277.

(4*S*,5*S*,6*S*)-5-[Benzyl(hydroxy)amino]-6-(hydroxymethyl)-2,2-dimethyltetrahydro-2*H*-pyran-4-ol (25)

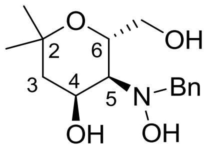

To a solution of **21** (70 mg, 0.252 mmol) in EtOH (2 mL), was added $NaBH_4$ (24 mg, 0.631 mmol) at 0 °C and the mixture stirred for 1 h at room temperature. The solvent was then removed in vacuo and CH_2Cl_2 and H_2O added to the residue. The layers were separated and the aqueous phase was extracted two times with CH_2Cl_2. The combined organic layers were dried (Na_2SO_4) and the solvent was removed in vacuo. Purification by column chromatography (silica gel, hexane/EtOAc = 1:1) yielded the product **25** (63 mg, 89%) as colorless crystals. M.p. 128–129 °C. $[\alpha]_D^{22}$ = +40.0 (c = 0.08, $CHCl_3$). ^{1}H NMR (700 MHz, $CDCl_3$): δ = 1.18, 1.51 (2 s, 3 H each, Me), 1.51 (dd, *J* = 3.1, 14.3 Hz, 1 H, 3-H), 1.88 (dd, *J* = 3.1, 14.3 Hz, 1 H, 3-H), 2.61 (dd, *J*

= 3.1, 10.3 Hz, 1 H, 5-H), 3.69 (dd, *J* = 5.3, 11.2 Hz, 1 H, 6-CH_2), 3.83 (dd, *J* = 5.3, 11.2 Hz, 1 H, 6-CH_2), 4.00, 4.27 (2 d, *J* = 13.1 Hz, 1 H each, NCH_2), 4.34 (td, *J* = 5.3, 10.3 Hz, 1 H, 6-H), 4.79 (q, *J* = 3.1 Hz, 1 H, 4-H), 7.27–7.43 (m, 5 H, Ph) ppm. ^{13}C NMR (176 MHz, $CDCl_3$): δ = 25.3, 32.1 (2 q, Me), 42.5 (t, C-3), 61.2 (t, NCH_2), 65.0 (d, C-5), 65.8 (t, 6-CH_2), 65.8 (d, C-6), 65.8 (d, C-4), 71.8 (s, C-2), 127.7, 128.5, 129.0, 136.9 (3 d, s, Ph) ppm. IR (KBr): 3440 cm^{-1} (OH), 3130–2850 (=C-H, C-H). ESI-TOF: *m/z* calc. for $C_{15}H_{23}NO_4$ $[M + Na]^+$ 304.1519, found 304.1523.

(4*S*,5*S*,6*S*)-5-Amino-6-(hydroxymethyl)-2,2-dimethyltetrahydro-2*H*-pyran-4-ol (26)

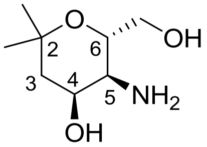

A suspension of palladium on charcoal (10% Pd, 60 mg) in MeOH (4 mL) was saturated with hydrogen for 1 h. After addition of compound **25** (60 mg, 0.214 mmol) in MeOH (2 mL), hydrogen was bubbled through the mixture for a further 30 min. The reaction mixture was then stirred under an atmosphere of hydrogen for 24 h. Filtration through a short pad of celite and concentration of the solution yielded **26** (35 mg, 94%) as a colorless oil. $[\alpha]_D^{22}$ = +173.8 (c = 1.8, MeOH). ^{1}H NMR (700 MHz, CD_3OD): δ = 1.19, 1.44 (2 s, 3 H each, Me), 1.66 (dd, *J* = 3.3, 14.4 Hz, 1 H, 3-H), 1.87 (dd, *J* = 3.3, 14.4 Hz, 1 H, 3-H), 2.84 (dd, *J* = 3.3, 10.0 Hz, 1 H, 5-H), 3.70–3.72 (m, 2 H, 6-CH_2), 3.80 (td, *J* = 4.5, 10.0 Hz, 1 H, 6-H), 4.06 (q, *J* = 3.3 Hz, 1 H, 4-H) ppm. ^{13}C NMR (176 MHz, CD_3OD): δ = 25.4, 32.3 (2 q, Me), 43.3 (t, C-3), 52.8 (d, C-5), 64.5 (t, 6-CH_2), 67.5 (d, C-4), 70.2 (d, C-6), 72.7 (s, C-2) ppm. IR (film): 3350 cm^{-1} (OH), 2990–2850 (C-H). ESI-TOF: *m/z* calc. for $C_8H_{17}NO_3$ $[M + H]^+$ 176.1281, found 176.1278.

(3*R*,4*S*,5*R*,6*S*)-5-[Benzyl(*tert*-butyldimethylsiloxy)amino]-6-(hydroxymethyl)-2,2-dimethyltetrahydro-2*H*-pyran-3,4-diol (27)

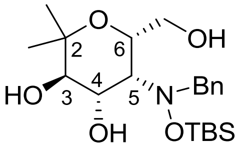

Compound **9** (90 mg, 0.220 mmol) was dissolved in ethanol (1.5 mL) and cooled to −40 °C. $CeCl_3$ (164 mg, 0.440 mmol) followed by $NaBH_4$ (17 mg, 0.440 mmol) were added and the mixture was stirred until it slowly reached room temperature (5 h). CH_2Cl_2 and H_2O were then added. The layers were separated and the aqueous phase was extracted two times with CH_2Cl_2. The combined organic layers were dried (Na_2SO_4), filtered and concentrated. Purification by column chromatography (silica gel, hexane/EtOAc = 2:1) yielded the product **27** (81 mg, 86%) as colorless crystals. M.p. 94–96 °C. $[\alpha]_D^{22}$ = +85.7 (c = 1.1, $CHCl_3$). ^{1}H NMR (500 MHz, $CDCl_3$): δ = −0.78, −0.03 (2 s_{br}, 3 H each, $SiMe_2$), 0.80 (s, 9 H, *t*Bu), 1.20, 1.34 (2 s, 3 H each, Me), 2.56 (s_{br}, 1 H), 3.11 (s_{br}, 1 H), 3.67–4.09 (m, 6 H), 4.25 (m_c, 1 H), 4.43 (s_{br}, 1 H) 7.21–7.34 (m, 5 H, Ph) ppm. ^{13}C NMR (126 MHz, $CDCl_3$): δ = −4.6 (q, $SiMe_2$), 17.3, 28.6 (2 q, Me), 17.6, 26.0 (s, q, *t*Bu), 63.4, 63.7, 64.9, 71.4, 127.8, 128.3, 130.5, 137.5 (3 d, s, Ph) ppm. IR (KBr): 3080 cm^{-1} (OH), 3070–2830 (=C-H, C-H). ESI-TOF: *m/z* calc. for $C_{21}H_{37}NO_5Si$ $[M + (2H) - (TBS)]^+$ 298.1649, found 298.1414.

(3*R*,4*S*,5*R*,6*S*)-5-Amino-6-(hydroxymethyl)-2,2-dimethyltetrahydro-2*H*-pyran-3,4-diol (28)

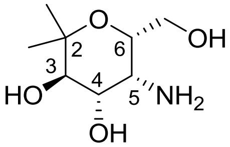

A suspension of palladium on charcoal (10% Pd, 50 mg) in MeOH (3 mL) was saturated with hydrogen for 1 h. After addition of compound **27** (50 mg, 0.121 mmol) in MeOH (2 mL), hydrogen was bubbled through the mixture for another 30 min. The reaction mixture was then stirred under an atmosphere of hydrogen for 24 h. Filtration through a short pad of celite and concentration of the solution yielded **28** (21 mg, 91%) as a colorless oil. $[\alpha]_D^{22}$ = +85.6 (c = 0.36, MeOH). ^{1}H NMR (500 MHz, D_2O): δ = 1.23, 1.31 (2 s, 3 H each, Me), 3.41 (d, *J* = 10.3 Hz, 1 H, 3-H), 3.69 (dd, *J* = 1.4, 4.6 Hz, 1 H, 5-H), 3.72 (m_c, 2 H, 6-CH_2), 4.02 (m_c, 1 H, 6-H), 4.04 (dd, *J* = 4.6, 10.3 Hz, 1 H, 4-H) ppm. ^{13}C NMR (101 MHz, D_2O): δ = 16.9, 26.9 (2 q, Me), 53.8 (d, C-

5), 61.3 (t, 6-CH_2), 67.2 (d, C-4), 68.3 (d, C-6), 73.2 (d, C-3), 77.5 (s, C-2) ppm. IR (film): 3400–3100 cm^{-1} (OH, NH), 2950–2800 (C-H). ESI-TOF: *m/z* calc. for $C_8H_{17}NO_4$ $[M + H]^+$ 192.1230, found 192.1233.

(3*S*,4*R*,5*S*,6*S*)-5-[Benzyl(*tert*-butyldimethylsiloxy)amino]-6-(hydroxymethyl)-2,2-dimethyltetrahydro-2*H*-pyran-3,4-diol (29)

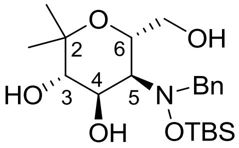

230 mg of the above described mixture of compounds **13** and **14** were dissolved in ethanol (5 mL) and cooled to 0 °C. $NaBH_4$ (42 mg, 1.13 mmol) was added and the mixture stirred for 1 h at room temperature. The solvent was then removed in vacuo and CH_2Cl_2 and H_2O added to the residue . The layers were separated and the aqueous phase was extracted two times with CH_2Cl_2. The combined organic layers were dried (Na_2SO_4) and the solvent was removed in vacuo. Purification by recrystallization (Et_2O) yielded **29** (190 mg, 82%) as colorless crystals. M.p. 127–128 °C. $[\alpha]_D^{22}$ = +89.2 (c = 0.44, $CHCl_3$). ^1H NMR (500 MHz, $CDCl_3$): δ = 0.21, 0.22 (2 s_{br}, 3 H each, $SiMe_2$), 0.91 (s, 9 H, *t*Bu), 1.12, 1.47 (2 s, 3 H each, Me), 2.10 (s_{br}, 1 H, OH), 2.26 (s_{br}, 1 H, OH), 2.72 (dd, *J* = 2.6, 10.8 Hz, 1 H, 5-H), 3.39–3.47 (m, 2 H, 3-H, 6-CH_2), 3.84 (d, *J* = 11.3 Hz, 1 H, 6-CH_2), 4.09 (ddd, *J* = 2.8, 6.5, 10.5 Hz, 1 H, 6-H), 4.17, 4.27 (2 d, *J* = 12.4 Hz, 1 H each, NCH_2), 4.66 (m_c, 1 H, 4-H), 5.18 (s_{br}, 1 H, OH), 7.19-7.31 (m, 5 H, Ph) ppm. ^{13}C NMR (126 MHz, $CDCl_3$): δ = −5.1, −4.1 (2 q, $SiMe_2$), 17.7, 25.8 (s, q, *t*Bu), 23.0, 26.6 (2 q, Me), 56.3 (d, C-5), 61.7 (t, NCH_2), 63.8 (t, 6-CH_2), 67.3 (d, C-6), 70.2 (d, C-4), 74.1 (d, C-3), 74.3 (s, C-2), 128.0, 128.7, 129.2, 135.9 (3 d, s, Ph) ppm. IR (KBr): 3440 cm^{-1} (OH), 3080–2830 (=C-H, C-H). ESI-TOF: *m/z* calc. for $C_{21}H_{37}NO_5Si$ $[M + Na]^+$ 434.2333, found 434.2341.

(3*S*,4*R*,5*R*,6*S*)-5-Amino-6-(hydroxymethyl)-2,2-dimethyltetrahydropyran-3,4-diol (30)

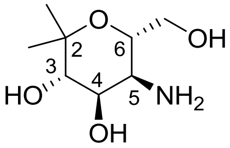

A suspension of palladium on charcoal (10% Pd, 60 mg) in MeOH (3 mL) was saturated with hydrogen for 1 h. After addition of compound **29** (60 mg, 0.146 mmol) in MeOH (2 mL),

hydrogen was bubbled through the mixture for another 30 min. The reaction mixture was then stirred under an atmosphere of hydrogen for 24 h. Filtration through a short pad of celite and concentration of the solution yielded **30** (29 mg, quant.) as colorless crystals. M.p. 148–149 °C. $[\alpha]_D^{22}$ = +64.4 (c = 0.23, MeOH). ^{1}H NMR (400 MHz, D_2O): δ = 1.09, 1.30 (2 s, 3 H each, Me), 3.01 (dd, *J* = 3.7, 9.9 Hz, 1 H, 5-H), 3.47 (d, *J* = 3.7 Hz, 1 H, 3-H), 3.58 (dd, *J* = 5.3, 11.9 Hz, 1 H, 6-CH_2), 3.65–3.75 (m, 2 H, 6-H, 6-CH_2), 3.90 (t, *J* = 3.7 Hz, 1 H, 4-H) ppm. ^{13}C NMR (101 MHz, D_2O): δ = 22.0, 25.8 (2 q, Me), 46.6 (d, C-5), 61.3 (t, 6-CH_2), 67.2 (d, C-4), 68.3 (d, C-6), 73.2 (d, C-3), 75.4 (s, C-2) ppm. IR (film): 3460–3240 cm^{-1} (OH, NH), 2990–2830 (C-H). ESI-TOF: *m/z* calc. for $C_8H_{17}NO_4$ $[M + H]^+$ 192.1230, found 192.1225.

***N1,N3,N5*-tris((2*S*,3*R*,4*R*)-4-hydroxy-2-hydroxymethyl-6,6-dimethyltetrahydro-2*H*-pyran-3-yl)benzene-1,3,5-tricarboxamide (31)**

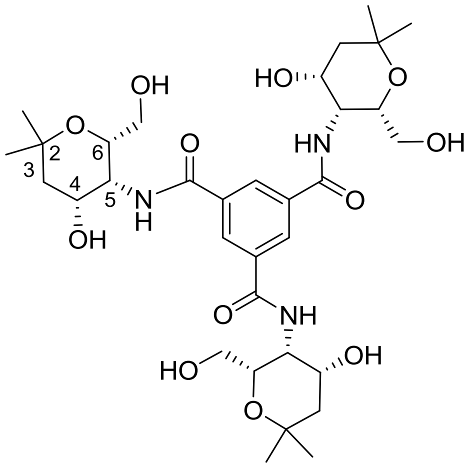

Compound **24** (120 mg, 0.685 mmol) was dissolved in pyridine (3 mL). After addition of HMDS (460 μL, 3.43 mmol) and TMSCl (414 μL, 3.43 mmol), the reaction mixture was stirred at room temperature until TLC analysis indicated complete consumption of **24** (ca. 5 h). The solvent was removed in vacuo and the product twice co-evaporated with toluene. The crude product (220 mg) was directly used in the next step. The obtained compound (220 mg) was dissolved in CH_2Cl_2 (4 mL) and the solution cooled to 0 °C. After the addition of NEt_3 (137 μL, 0.988 mmol) and 1,3,5-benzenetricarboxylic acid chloride (61 mg, 0.229 mmol), the reaction mixture was stirred for 3 h at room temperature. The solvent was removed in vacuo and the resulting crude product (296 mg) used in the next step without purification. To the obtained compound (296 mg), was added a mixture of methanol and TFA (5 mL, 9:1) and the reaction stirred for 3 h at room temperature. After removal of the solvent in vacuo, crystallization from methanol yielded **31** (120 mg, 77%

overall yield) as colorless crystals. M.p. 210 °C. $[\alpha]_D^{22}$ = +66.7 (c = 0.40, DMF). ^{1}H NMR (700 MHz, DMF-d_7): δ = 1.35, 1.63 (2 s, 9 H each, Me), 1.88 (dd, J = 3.0, 14.1 Hz, 3 H, 3-H), 2.06 (dd, J = 3.0 Hz, 14.1 Hz, 3 H, 3-H), 3.77–3.84 (m, 6 H, 6-CH_2), 4.13 (m_c, 3 H, 6-H), 4.22 (m_c, 3 H, 5-H), 4.36 (m_c, 3 H, 4-H), 8.31 (s_{br}, 3 H, NH), 8.84 (s, 3 H, Ar) ppm. ^{13}C NMR (127 MHz, DMF-d_7): δ = 24.9, 32.1 (2 q, Me), 42.7 (t, C-3), 50.8 (d, C-5), 63.3 (t, 6-CH_2), 66.3 (d, C-4), 69.7 (d, C-6), 71.3 (s, C-2), 129.3, 135.4 (d, s, Ar), 166.1 (s, NCOR) ppm. IR (ATR): 3370 cm^{-1} (OH), 3000–2840 (C-H). ESI-TOF: *m/z* calc. for $C_{33}H_{51}N_3O_{12}$ $[M + Na]^+$ 704.3365, found 704.3345.

***N1,N3,N5*-tris((2*S*,3*R*,4*S*,5*R*)-4,5-dihydroxy-2-hydroxymethyl-6,6-dimethyltetrahydro-2*H*-pyran-3-yl)benzene-1,3,5-tricarboxamide (32)**

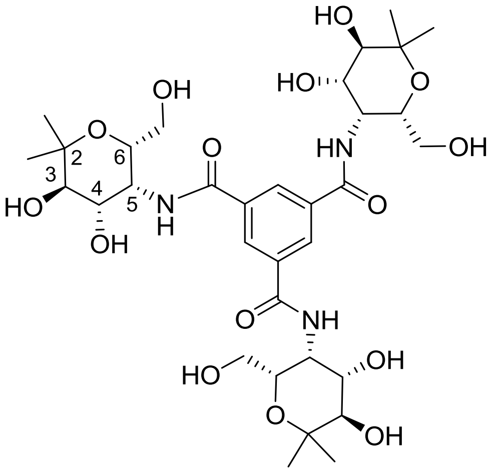

Compound **28** (112 mg, 0.586 mmol) was dissolved in pyridine (2.5 mL). After addition of HMDS (944 mg, 5.86 mmol) and TMSCl (749 μL, 5.86 mmol), the reaction mixture was stirred at room temperature until TLC analysis indicated complete consumption of **28** (ca. 5 h). The solvent was removed in vacuo and the product twice co-evaporated with toluene. The crude product (201 mg) was directly used in the next step. The obtained compound (201 mg) was dissolved in CH_2Cl_2 (3 mL) and the solution cooled to 0 °C. After the addition of NEt_3 (137 μL, 0.988 mmol) and 1,3,5-benzenetricarboxylic acid chloride (43 mg, 0.165 mmol), the reaction mixture was stirred for 3 h at room temperature. The solvent was removed in vacuo and the resulting crude product (162 mg) used in the next step without purification. To 122 mg of the obtained compound, was added a mixture of methanol and TFA (1.5 mL, 9:1) and the reaction stirred for 3 h at room temperature. Crystallization from methanol yielded **32** (16 mg, 16%

overall yield) as colorless crystals. M.p. 262 °C. $[\alpha]_D^{22}$ = +150.8 (c = 0.7, H_2O). ^{1}H NMR (700 MHz, D_2O): δ = 1.35, 1.42 (2 s, 9 H each, Me), 3.63 (d, *J* = 10.4 Hz, 3 H, 3-H), 3.67–3.74 (m, 6 H, 6-CH_2), 4.10–4.17 (m, 6 H, 6-H, 4-H), 4.75 (dd, *J* = 3.2 Hz, 3 H, 5-H), 8.35 (s, 3 H, Ar) ppm. ^{13}C NMR (127 MHz, D_2O): δ = 17.0, 27.3 (2 q, Me), 52.4 (d, C-5), 61.5 (t, 6-CH_2), 69.7, 71.3 (2 d, C-6, C-4), 74.3 (d, C-3), 77.4 (s, C-2), 129.7, 134.8 (d, s, Ar) ppm. IR (film): 3350 cm^{-1} (OH, NH), 3000–2840 (C-H). ESI-TOF: *m/z* calc. for $C_{33}H_{51}N_3O_{15}$ $[M + Na]^+$ 752.3212, found 752.3196.

^{1}H and ^{13}C NMR spectra:

^{13}C NMR spectra recorded at 101 MHz show signals at 27.5, 103.5 and 179.1 ppm, which are caused by external electromagnetic interference.

500 MHz, $CDCl_3$:

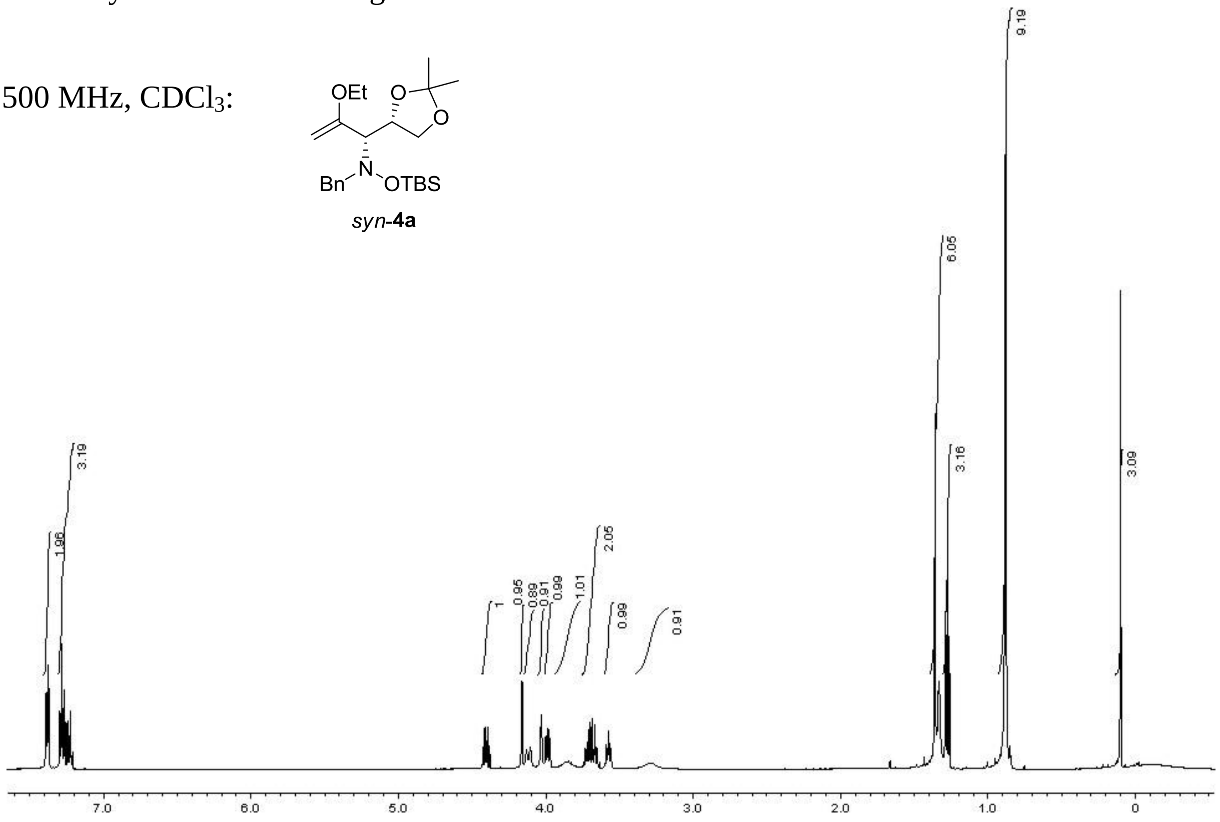

176 MHz, $CDCl_3$:

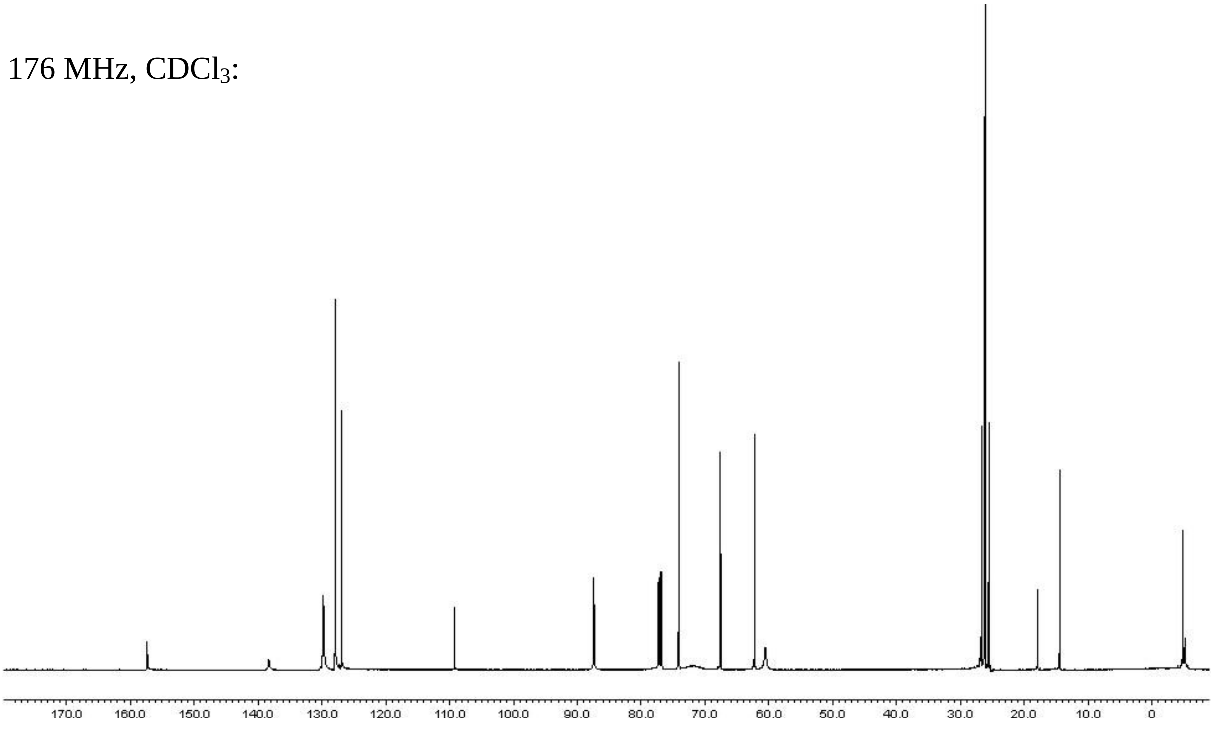

500 MHz, $CDCl_3$:

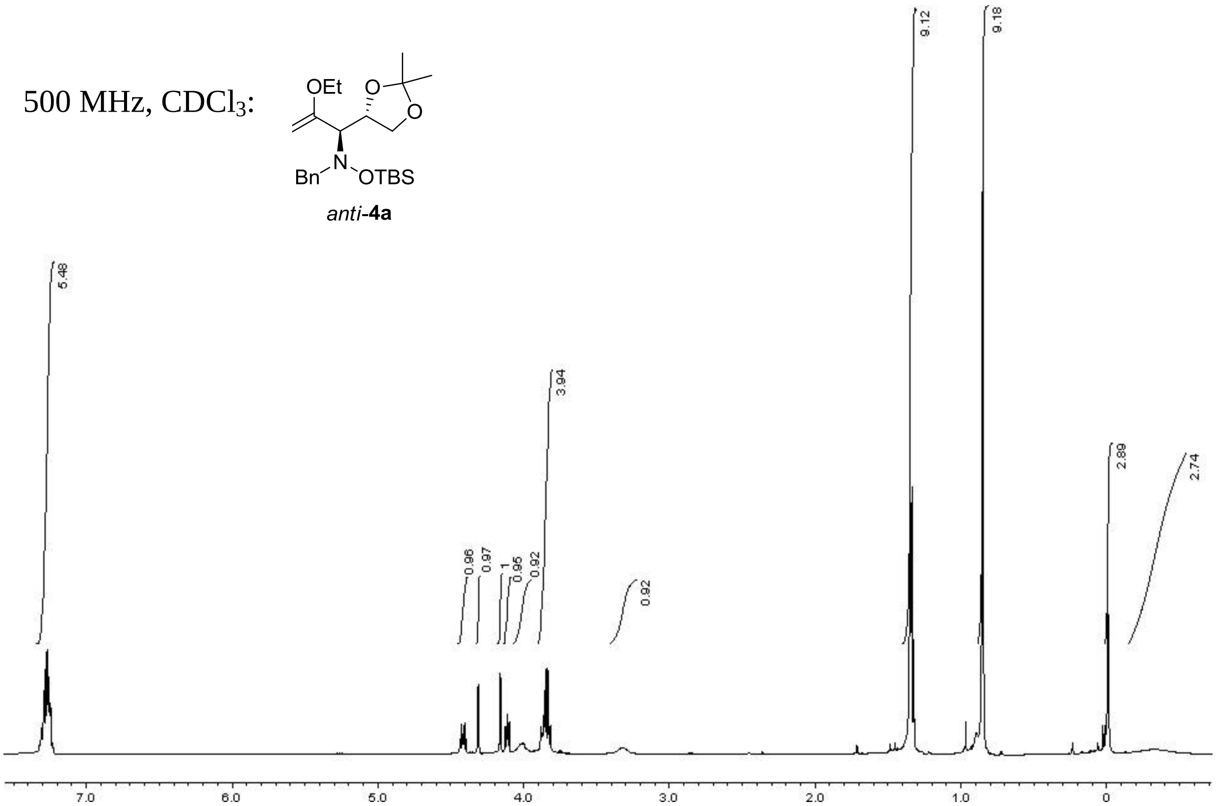

126 MHz, $CDCl_3$:

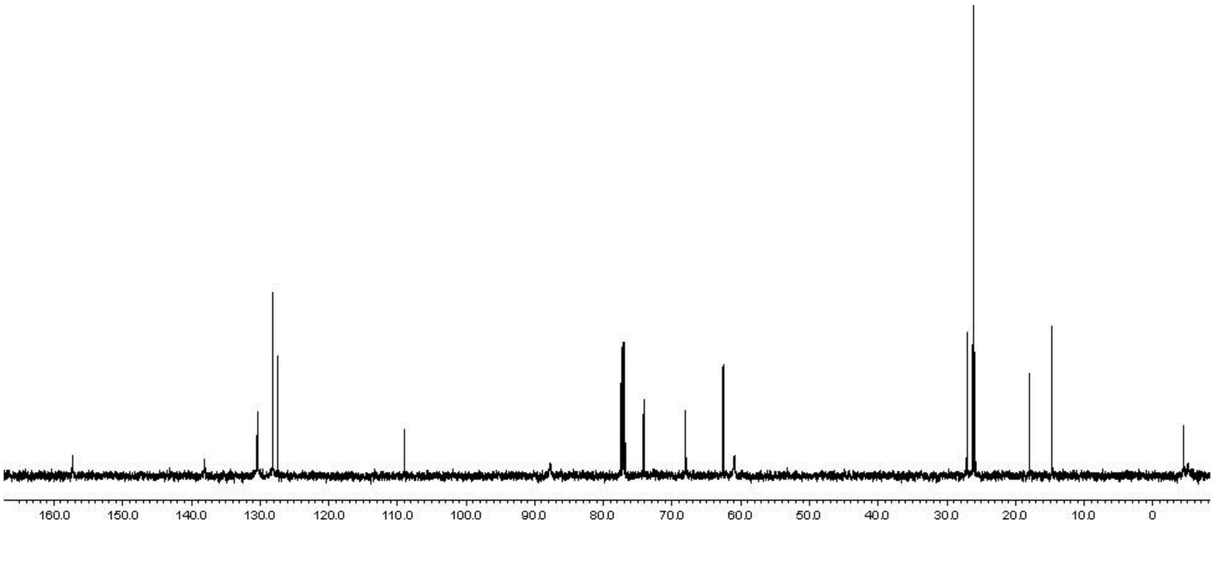

500 MHz, $CDCl_3$:

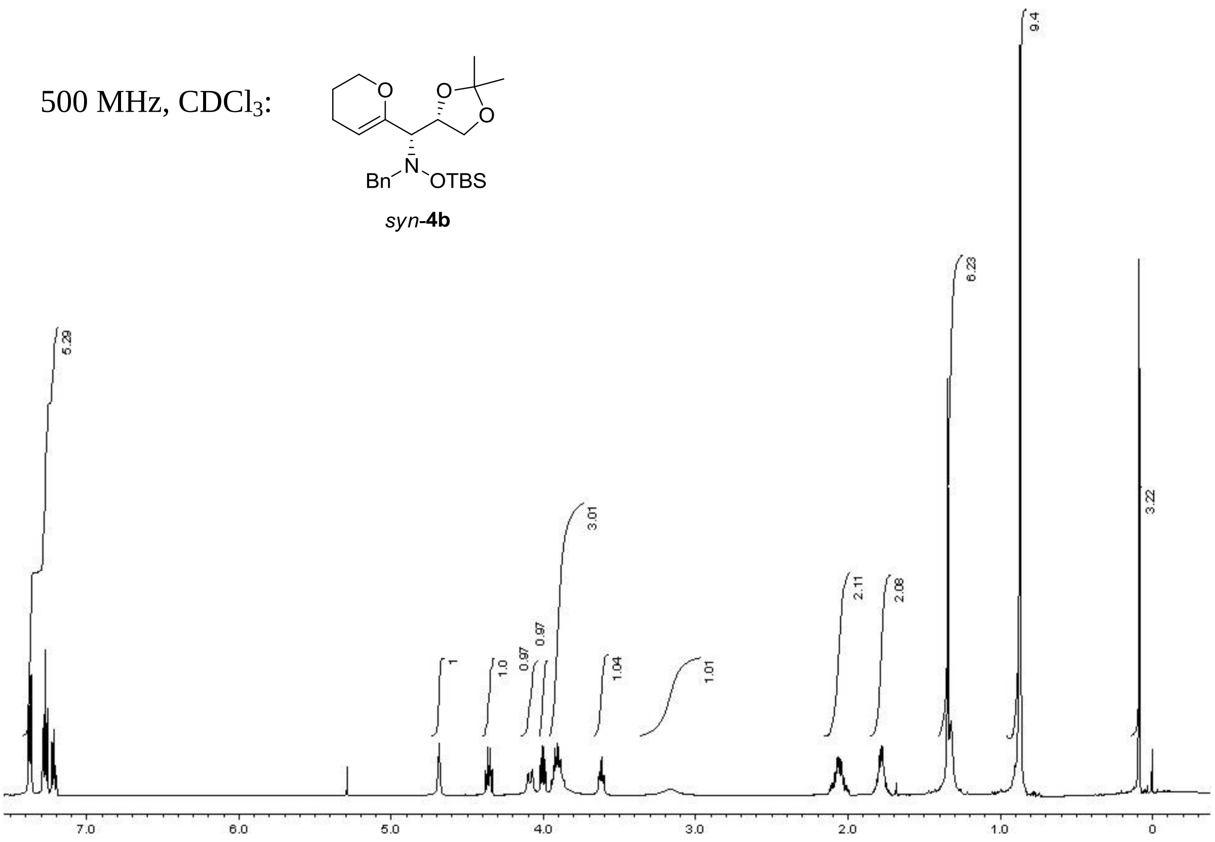

126 MHz, $CDCl_3$:

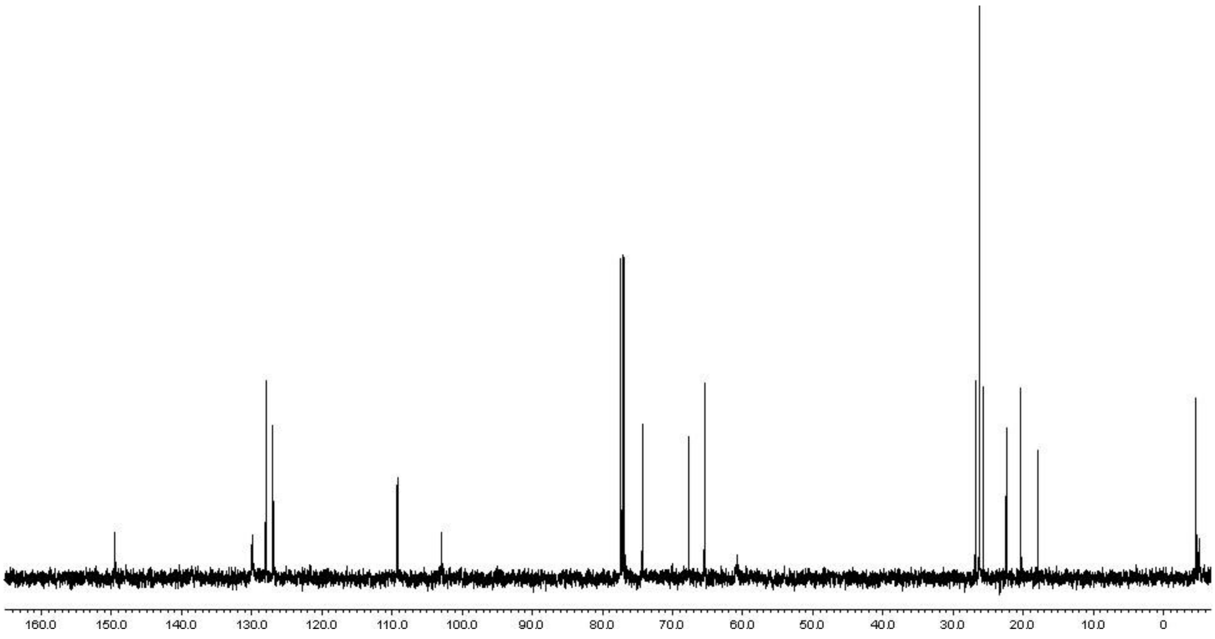

500 MHz, $CDCl_3$:

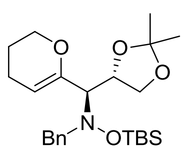

anti-**4b**

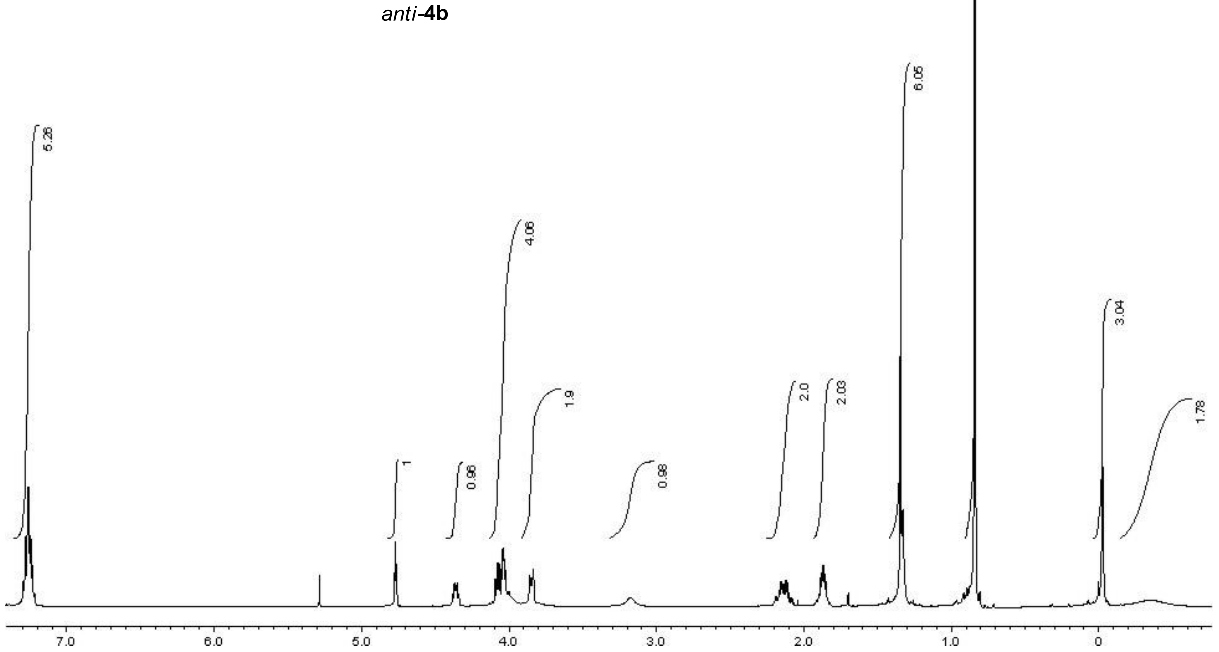

126 MHz, $CDCl_3$:

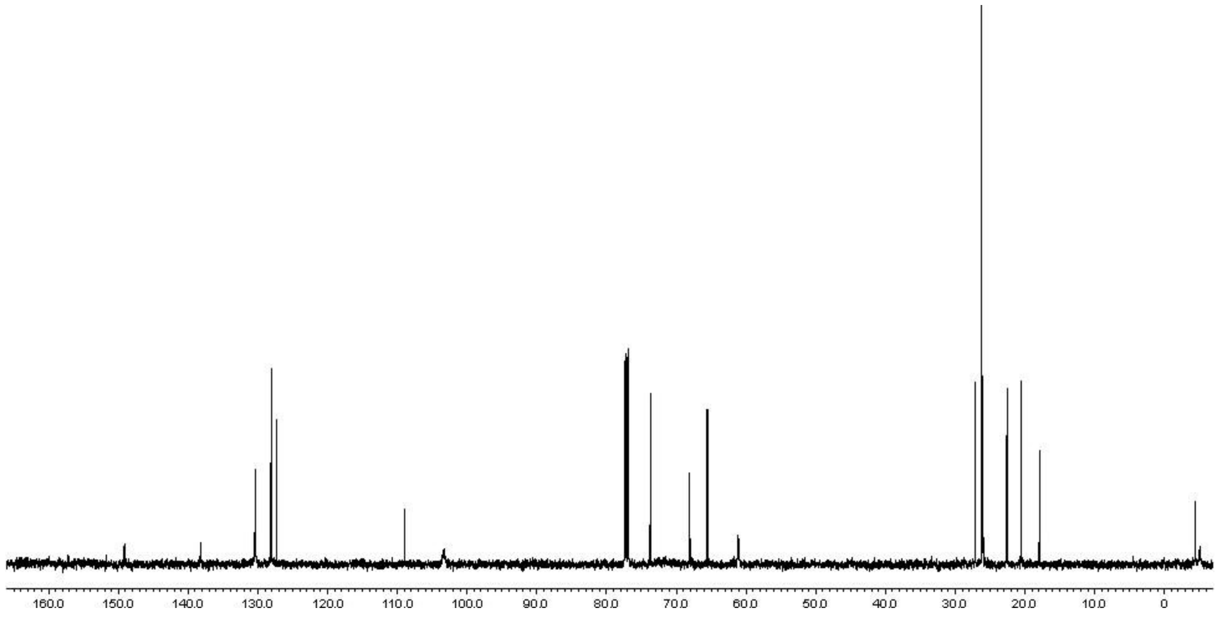

500 MHz, $CDCl_3$:

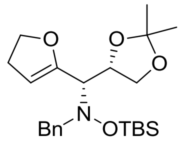

syn-**4c**

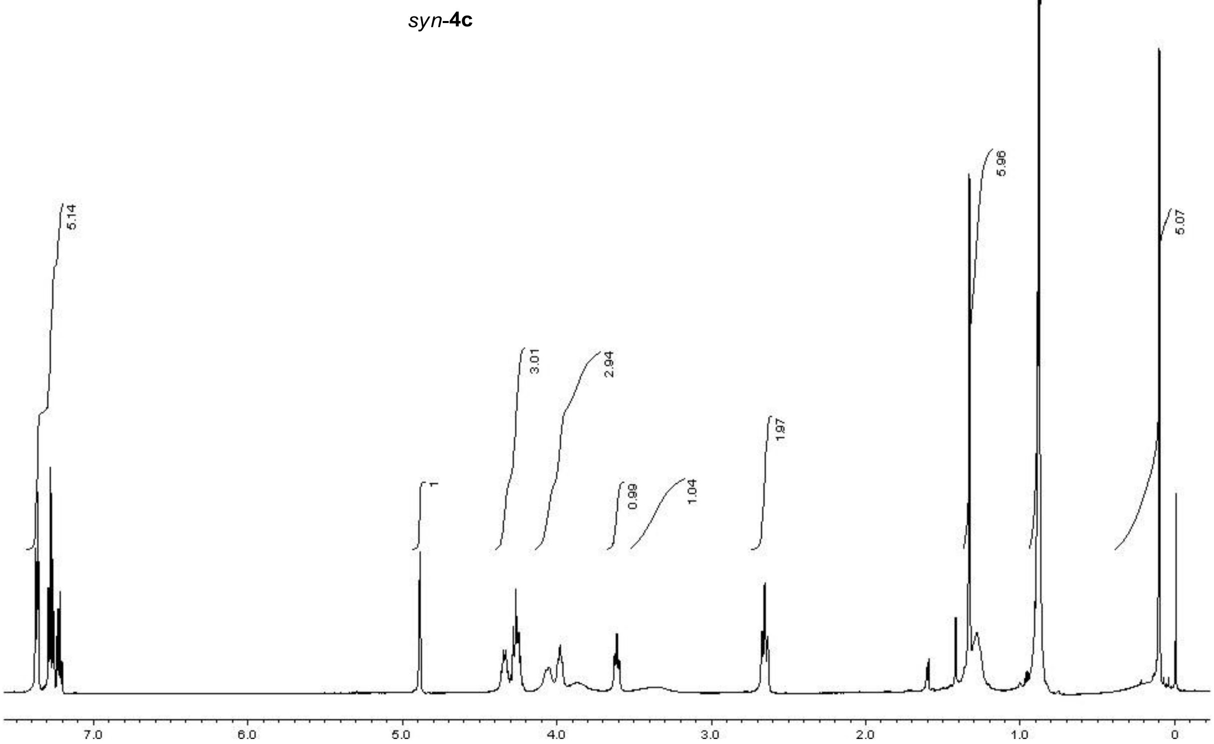

126 MHz, $CDCl_3$:

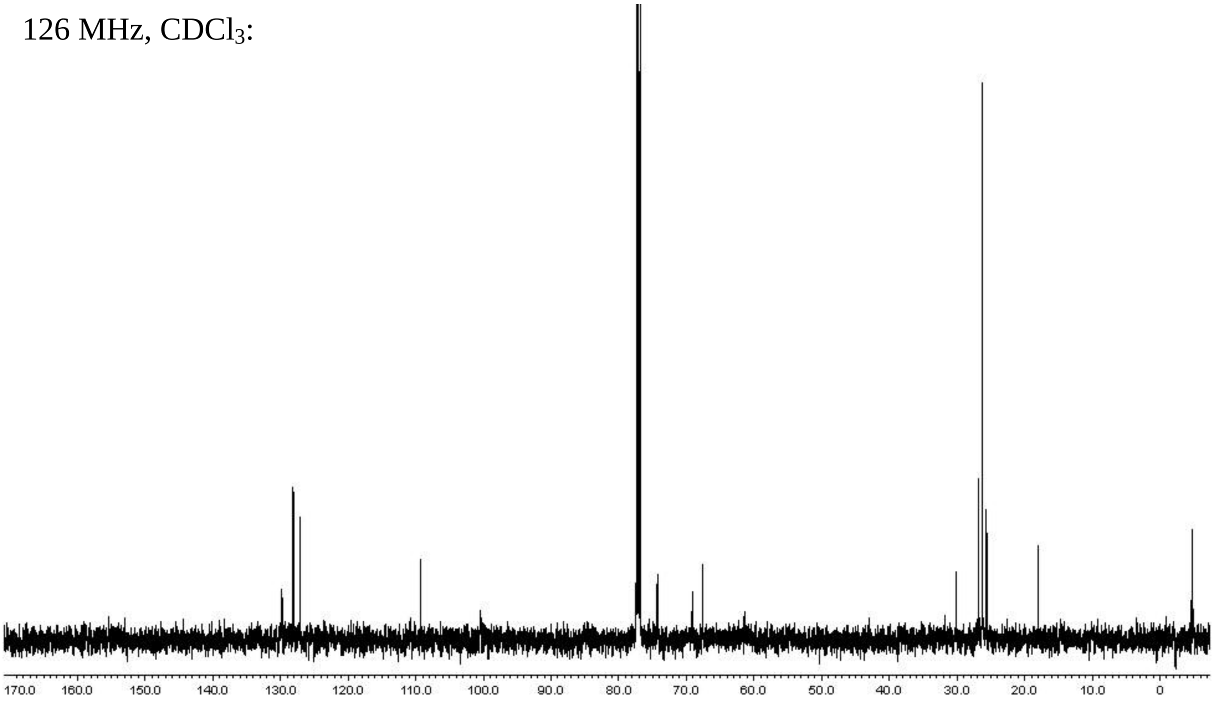

500 MHz, $CDCl_3$:

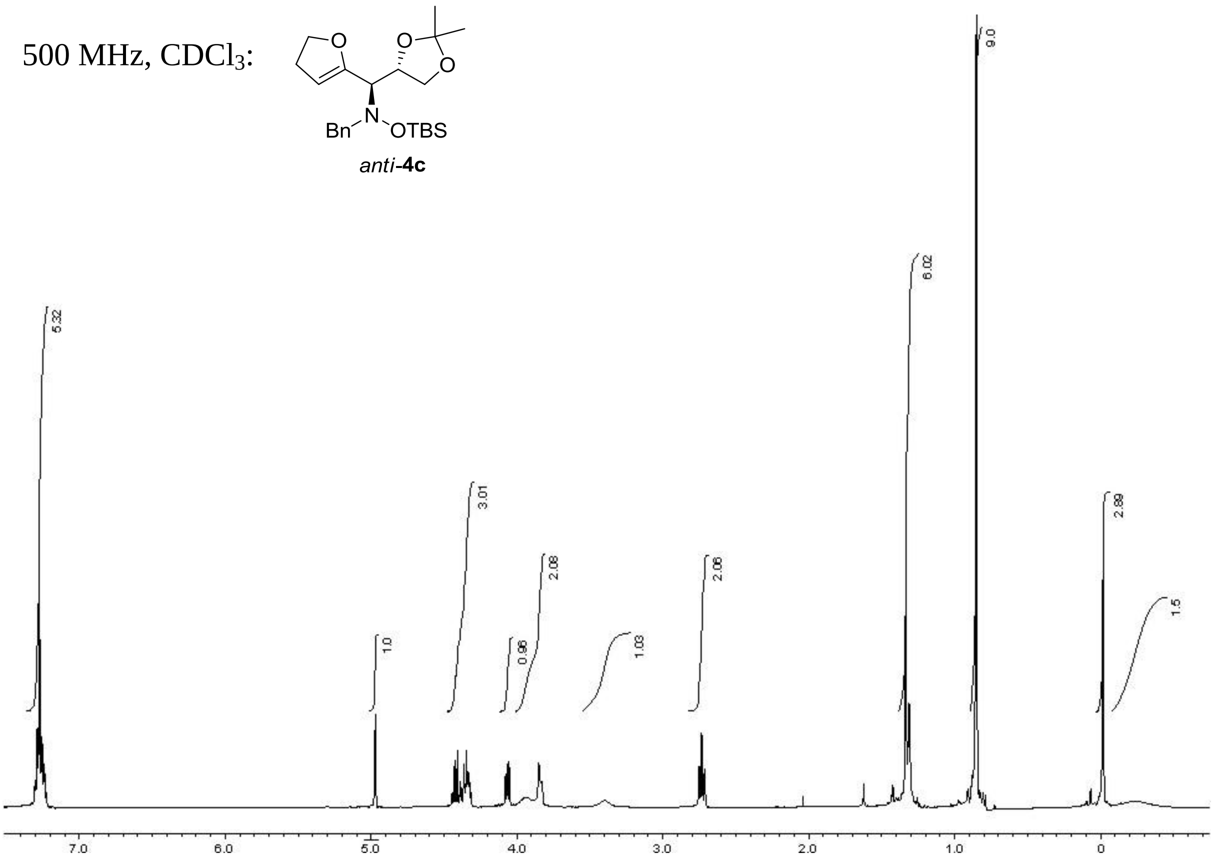

anti-**4c**

126 MHz, $CDCl_3$:

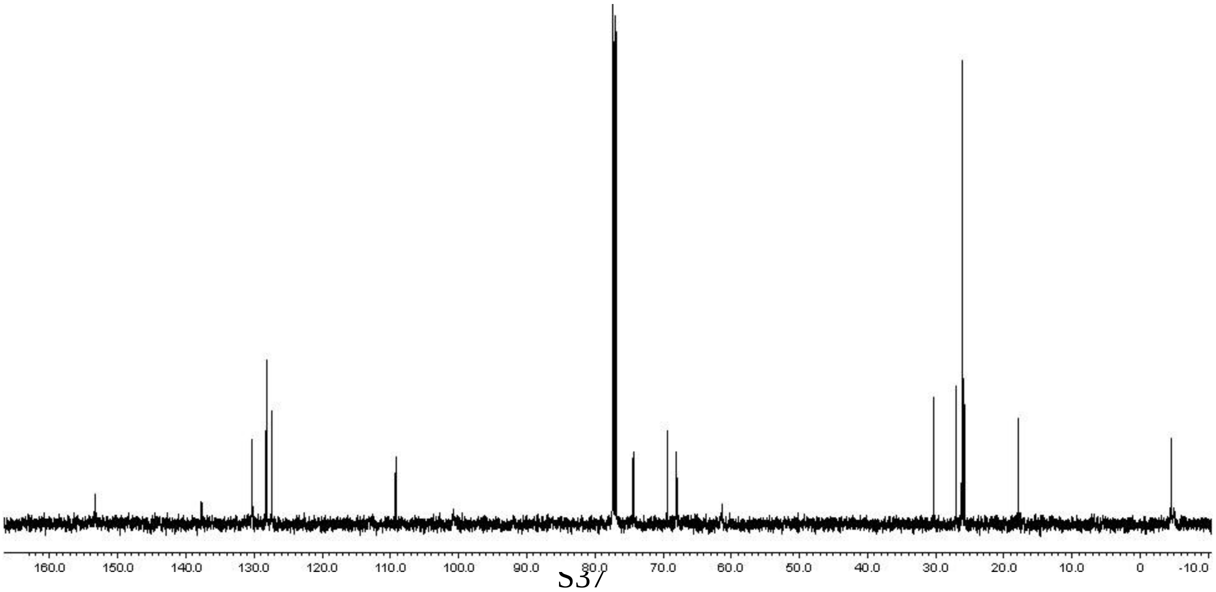

500 MHz, $CDCl_3$:

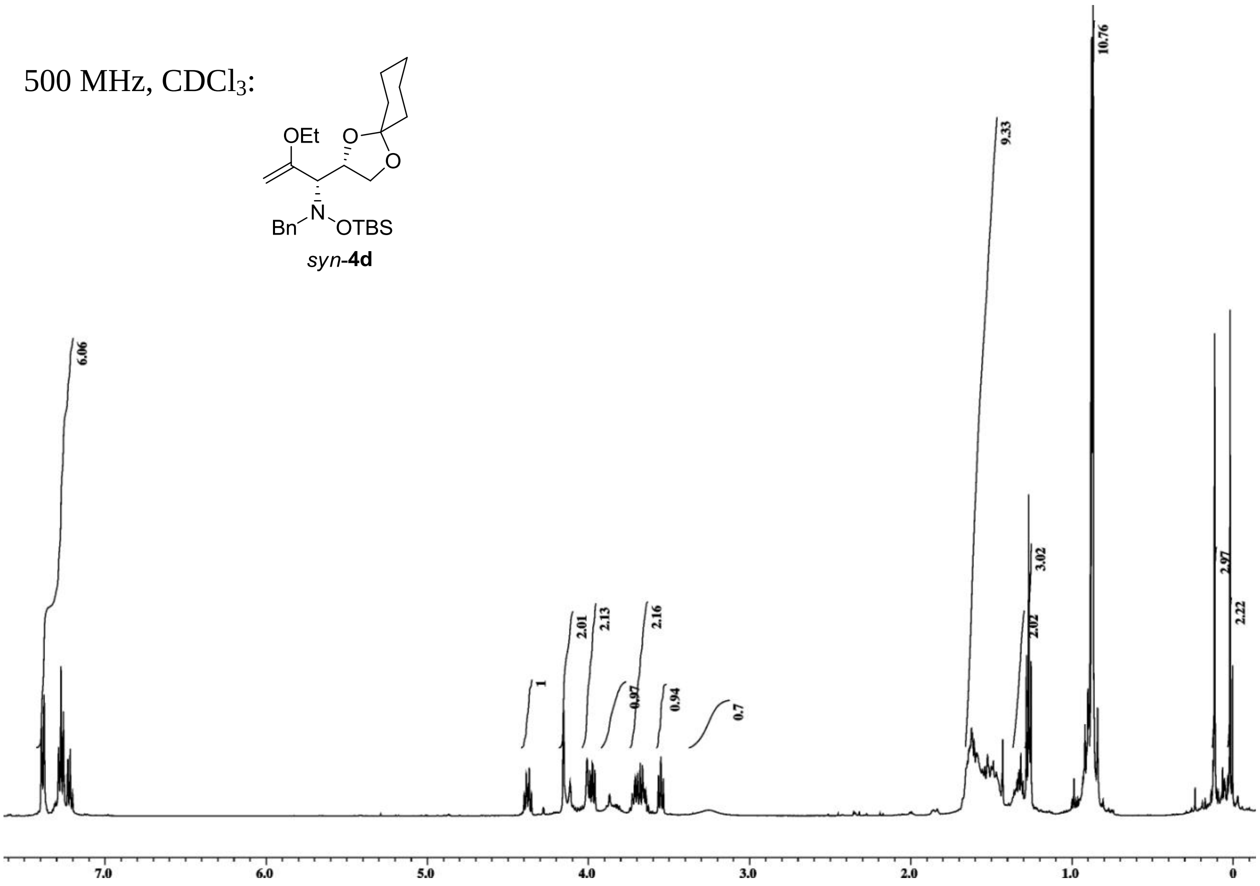

syn-**4d**

126 MHz, $CDCl_3$:

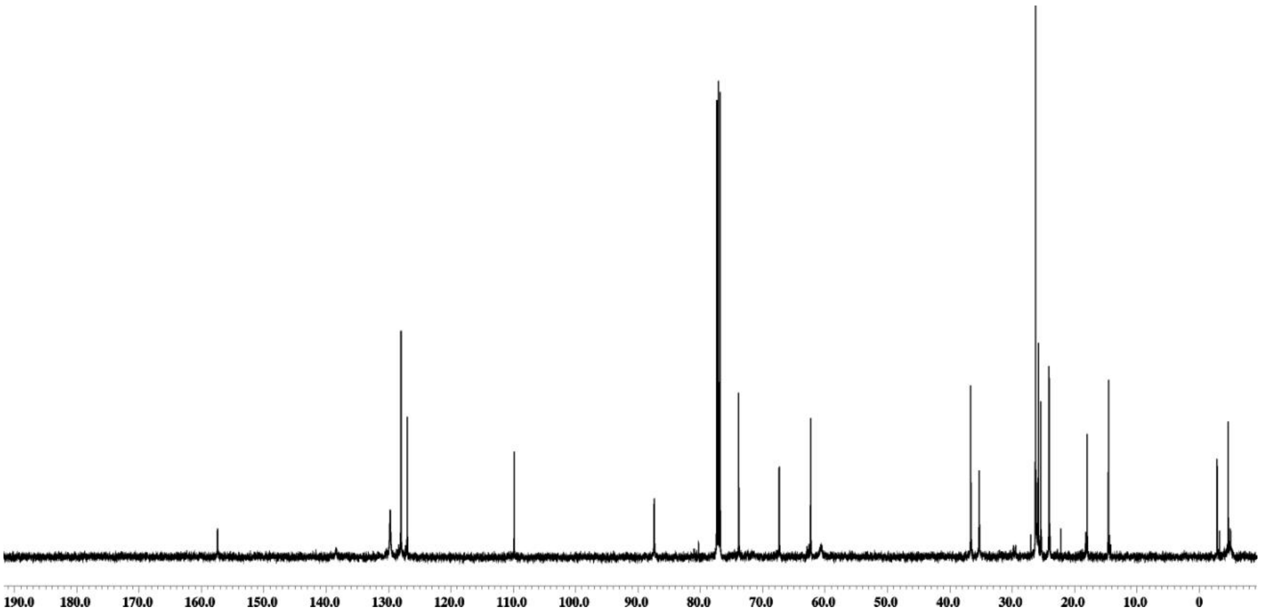

500 MHz, $CDCl_3$:

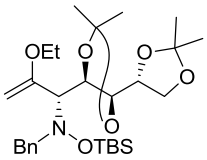

anti-**4e**

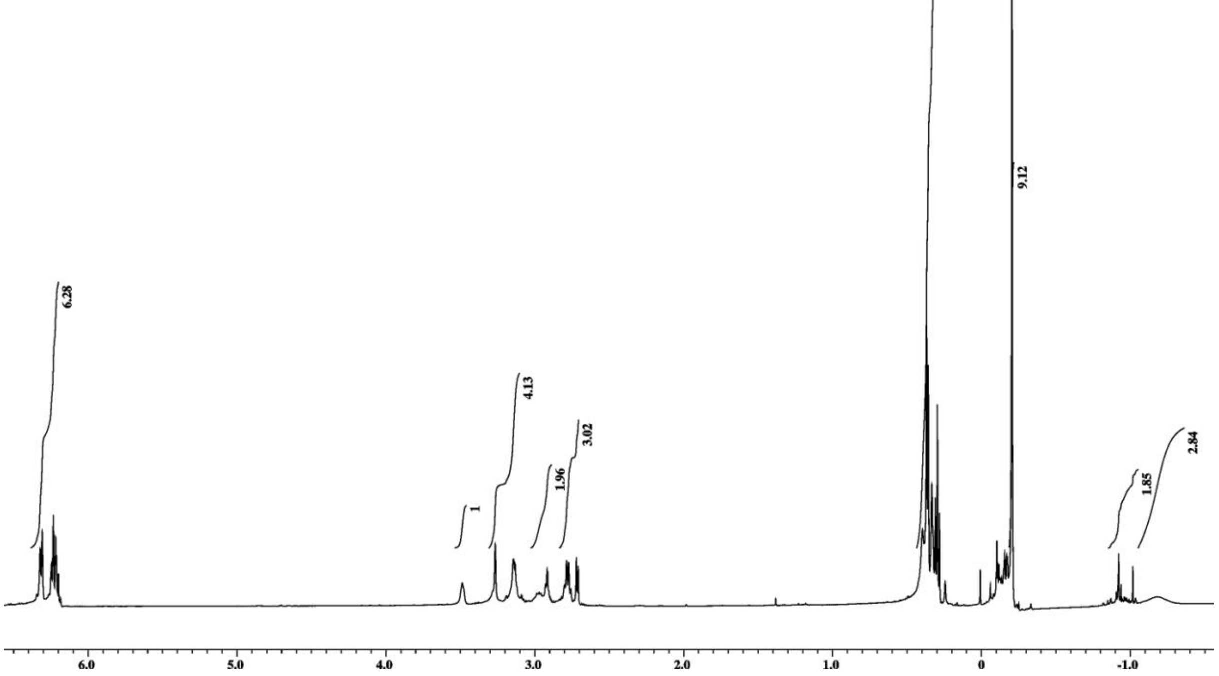

126 MHz, $CDCl_3$:

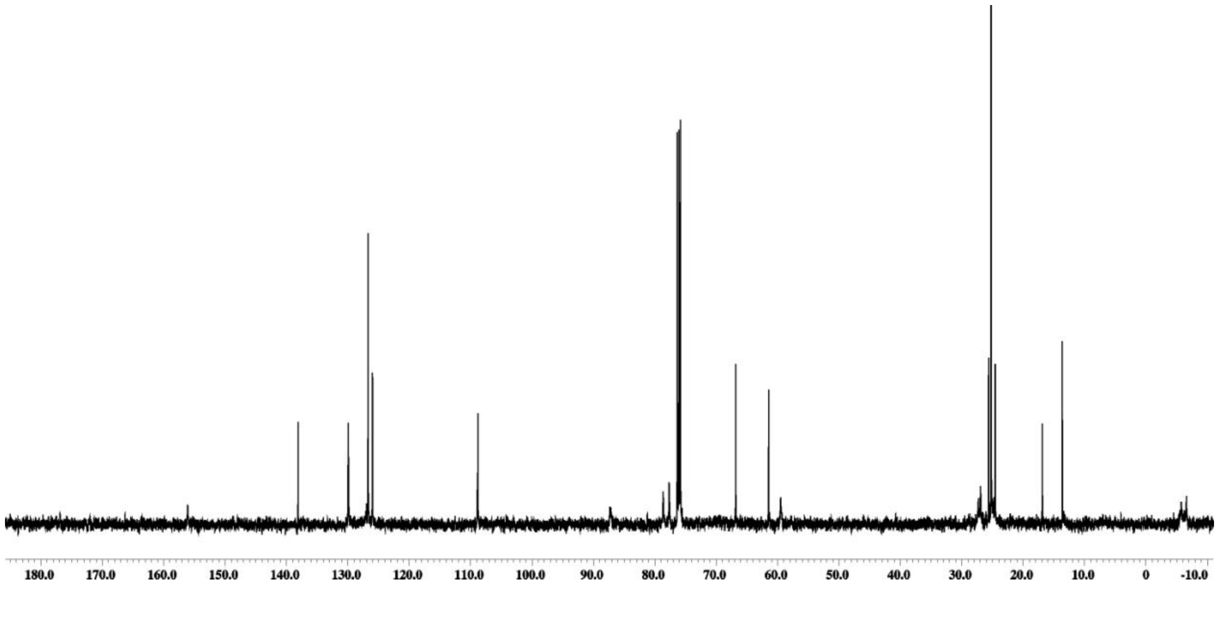

400 MHz, $CDCl_3$:

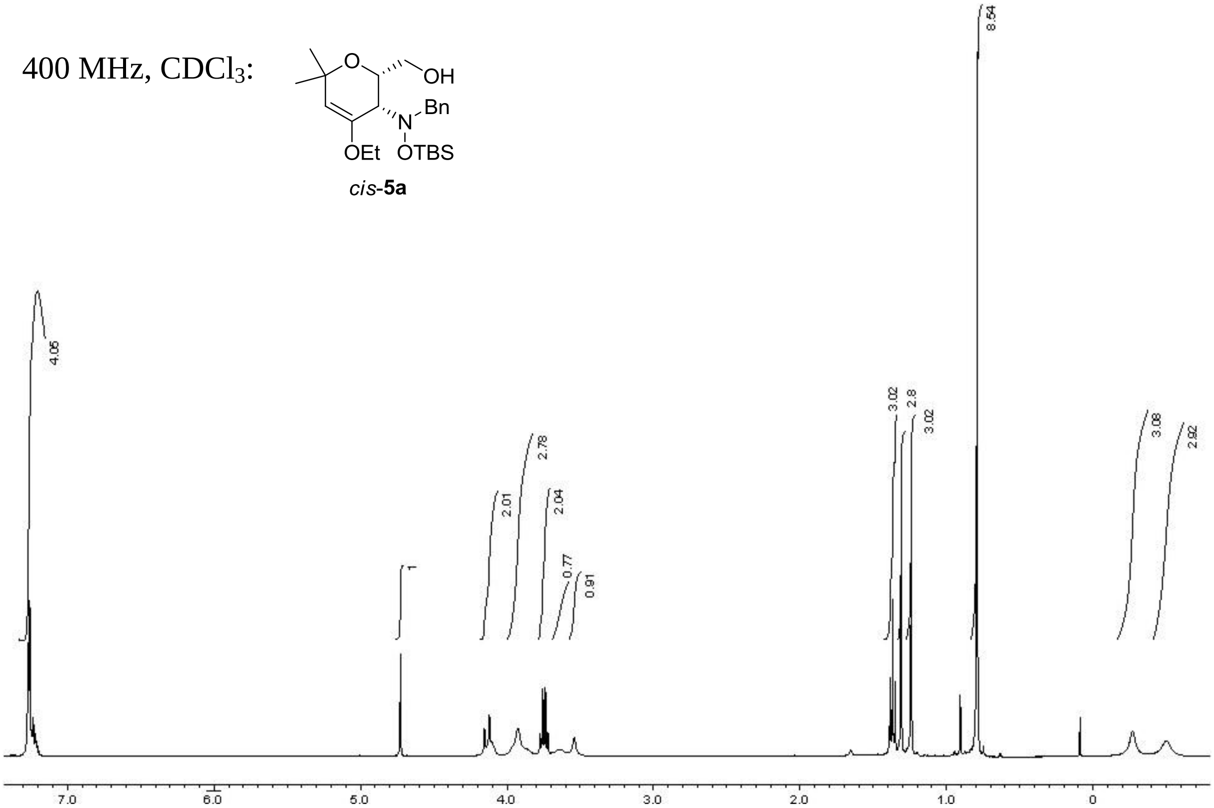

cis-**5a**

101 MHz, $CDCl_3$:

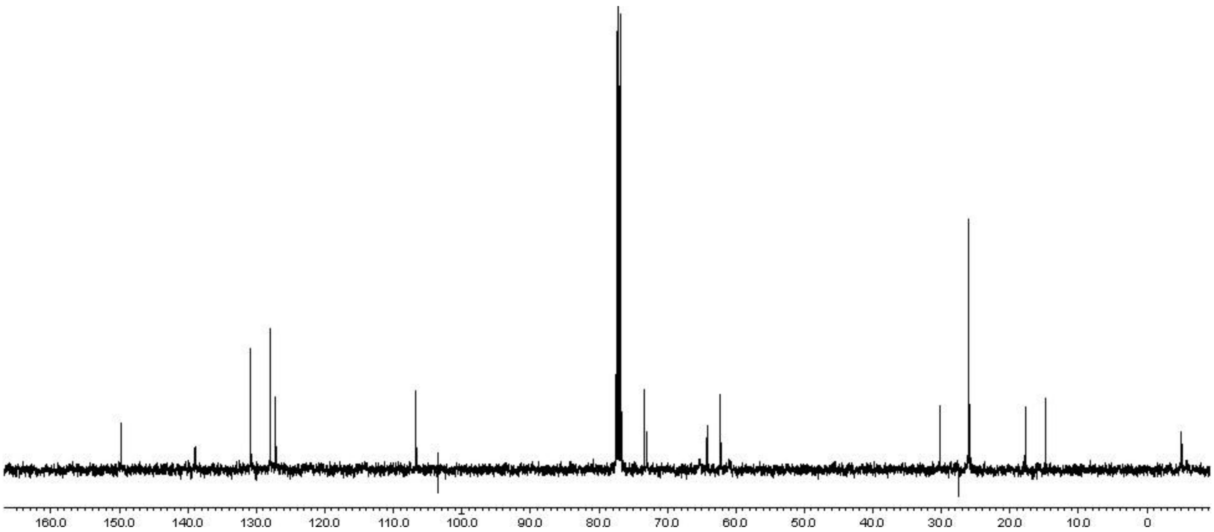

500 MHz, CDCl$_3$:

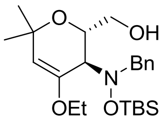

trans-**5a**

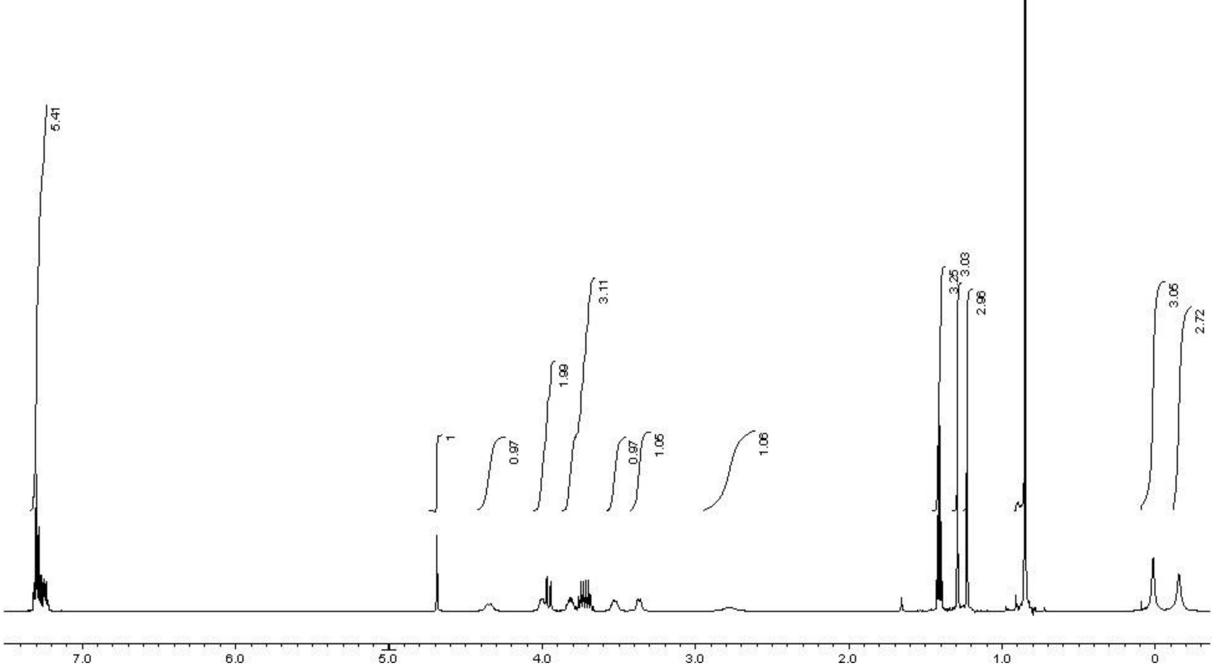

126 MHz, CDCl$_3$:

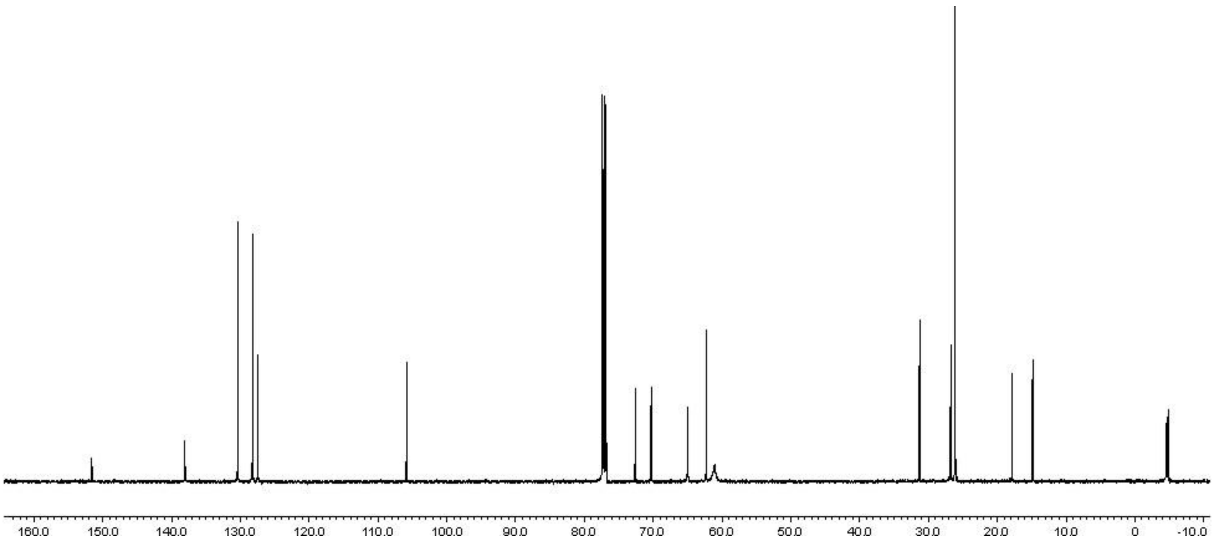

500 MHz, $CDCl_3$:

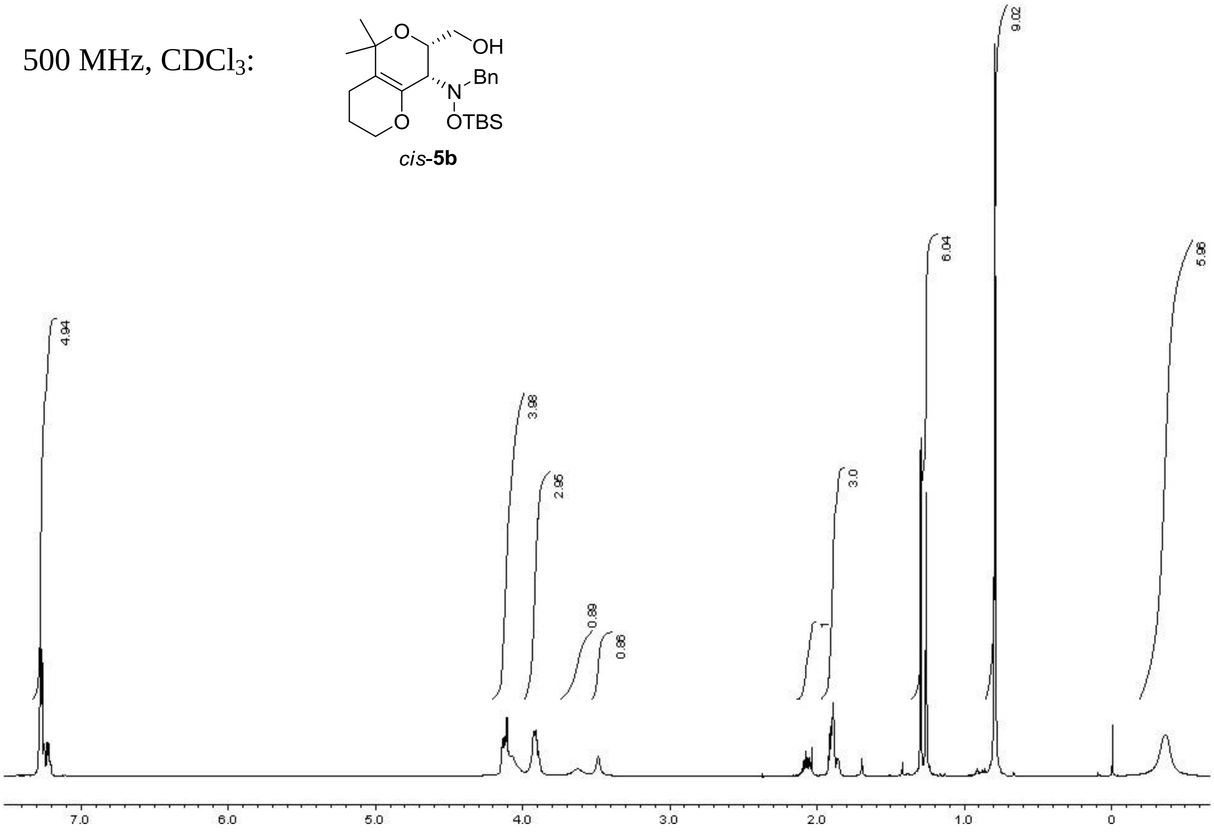

101 MHz, $CDCl_3$:

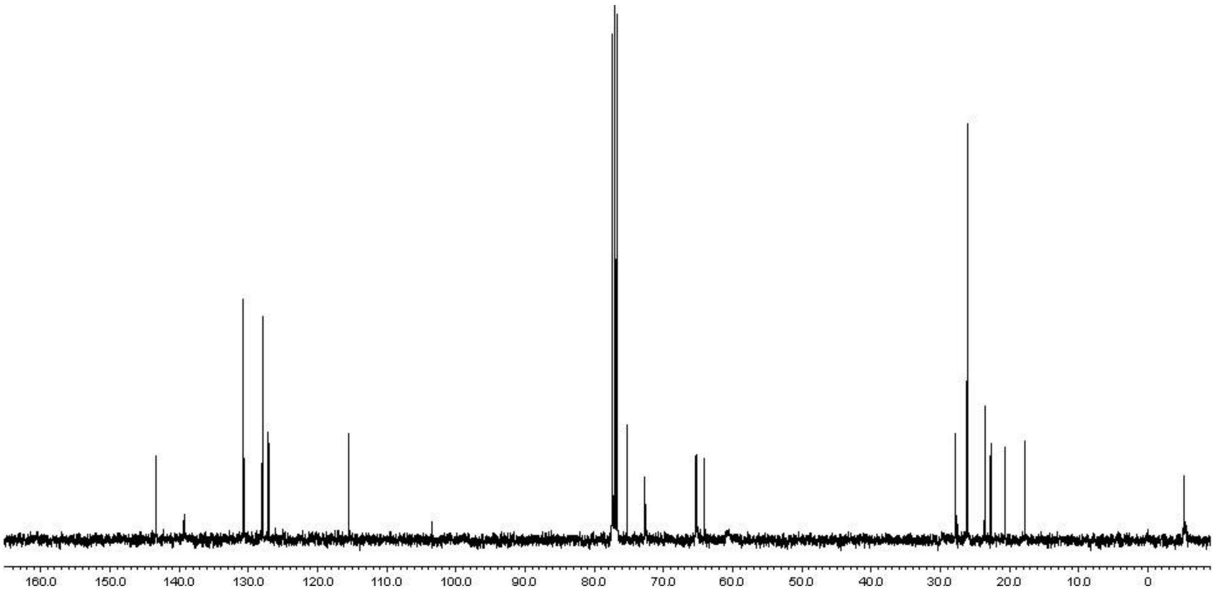

500 MHz, $CDCl_3$:

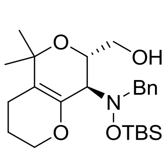

trans-**5b**

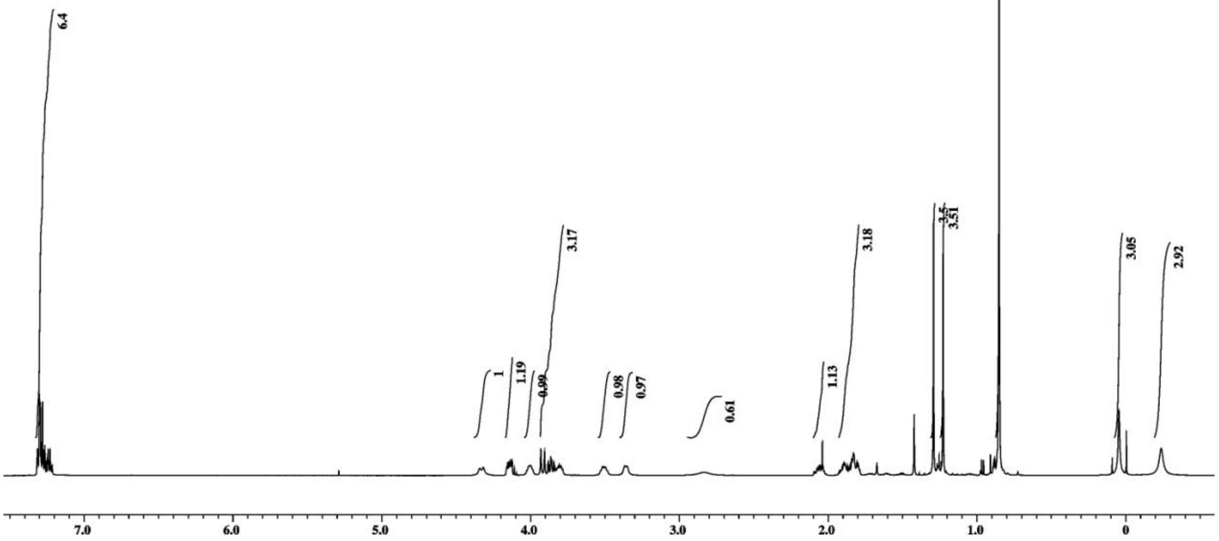

101 MHz, $CDCl_3$:

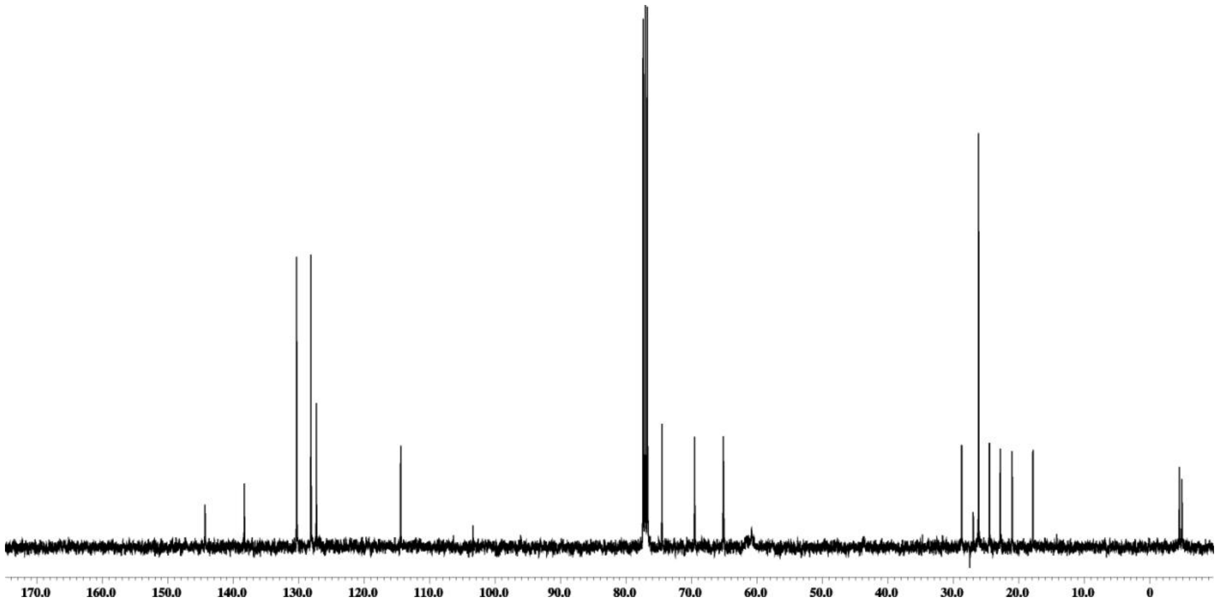

500 MHz, $CDCl_3$:

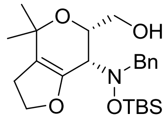

cis-**5c**

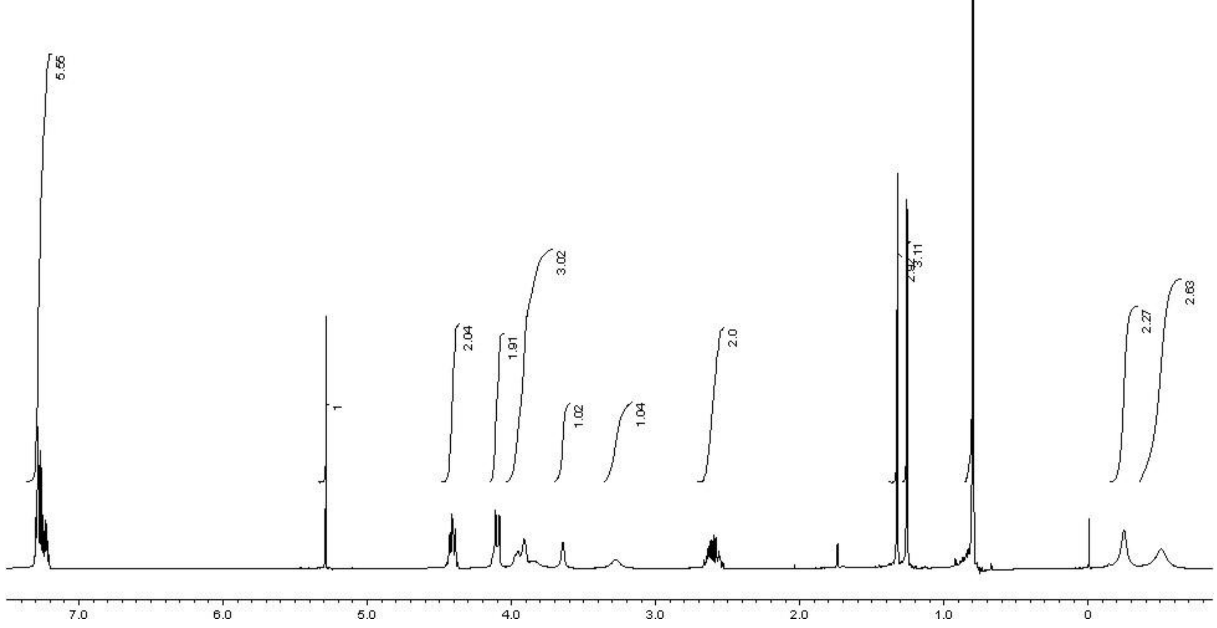

101 MHz, $CDCl_3$:

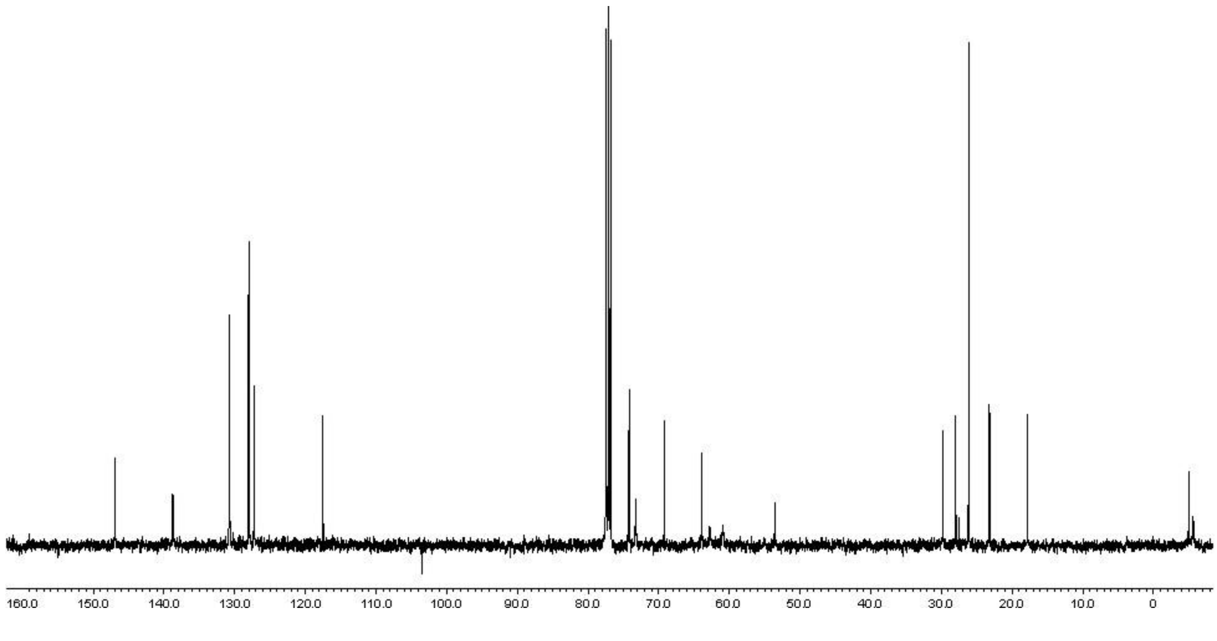

500 MHz, $CDCl_3$:

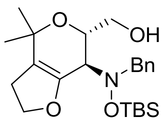

trans-**5c**

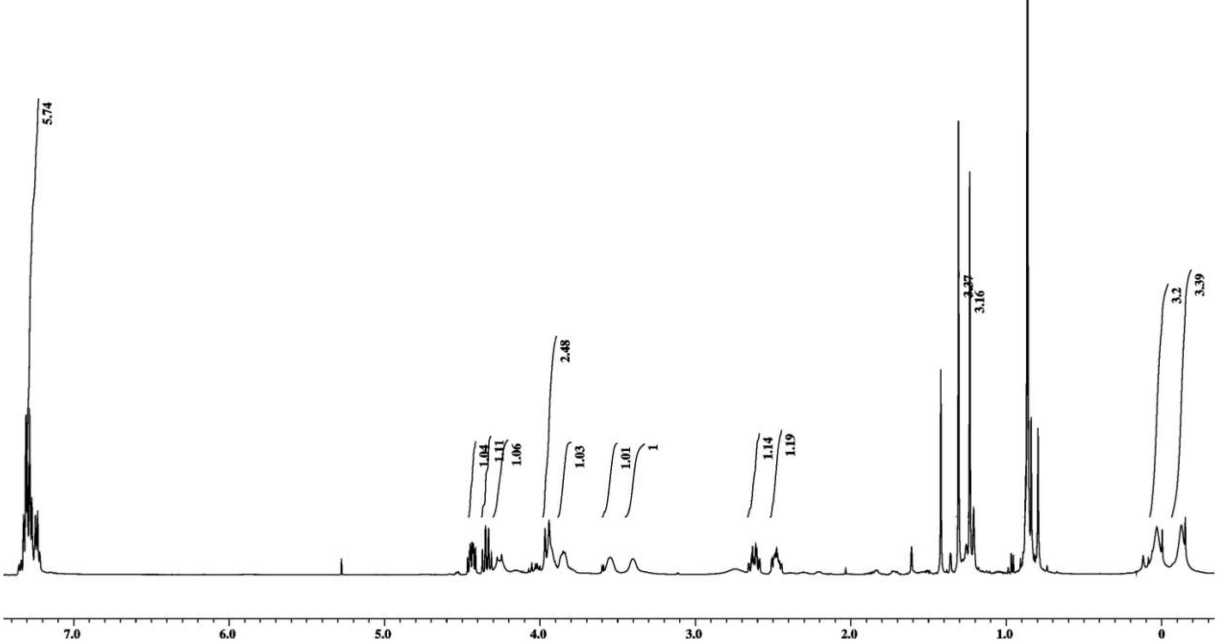

101 MHz, $CDCl_3$:

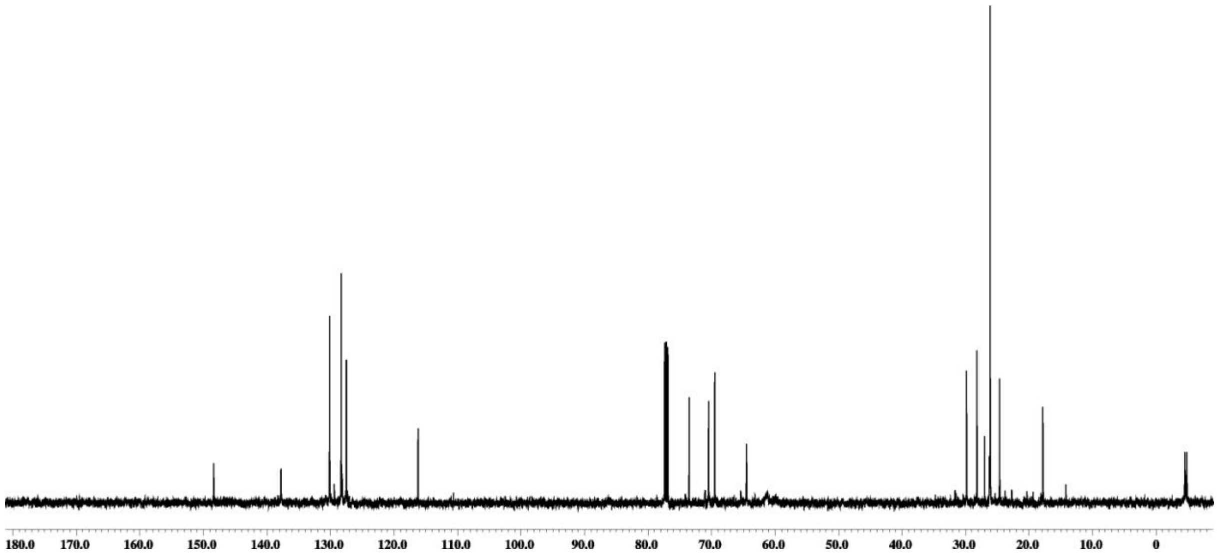

500 MHz, $CDCl_3$:

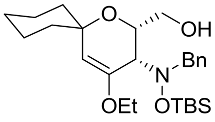

cis-**5d**

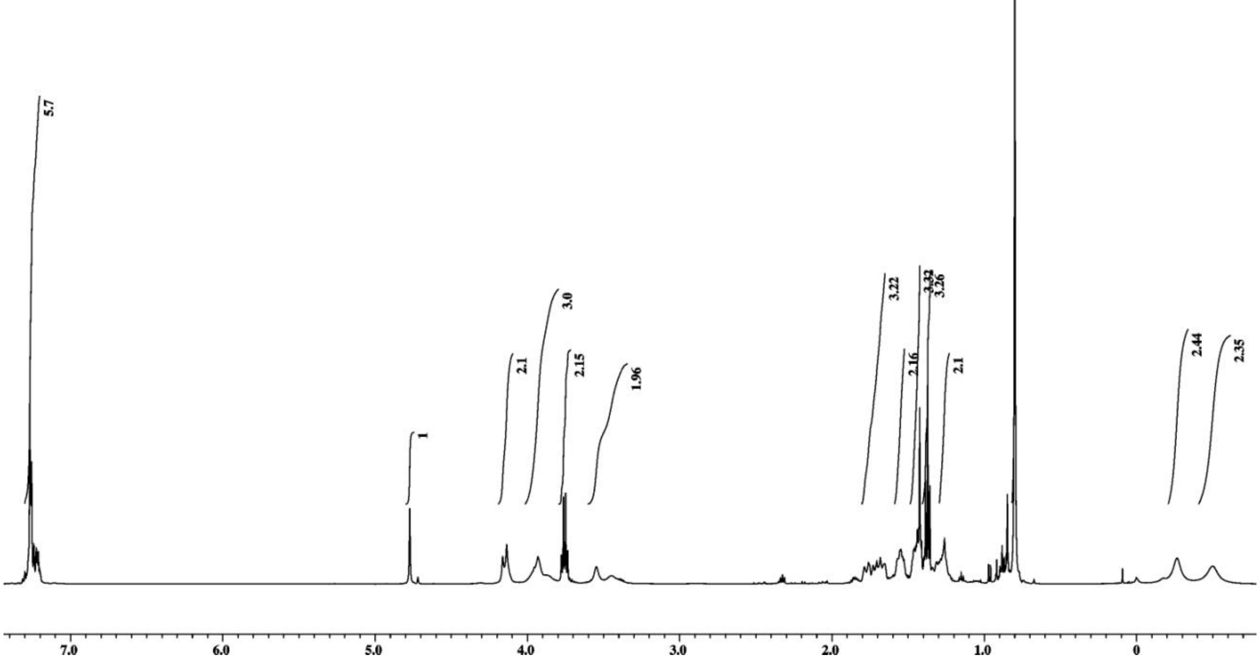

126 MHz, $CDCl_3$:

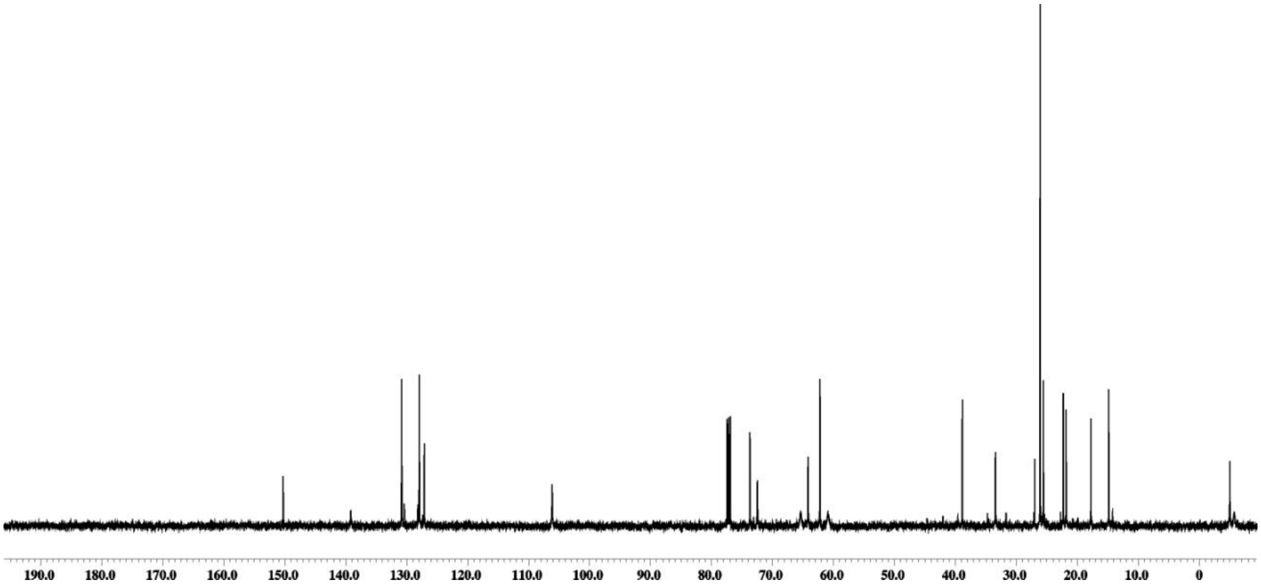

500 MHz, $CDCl_3$:

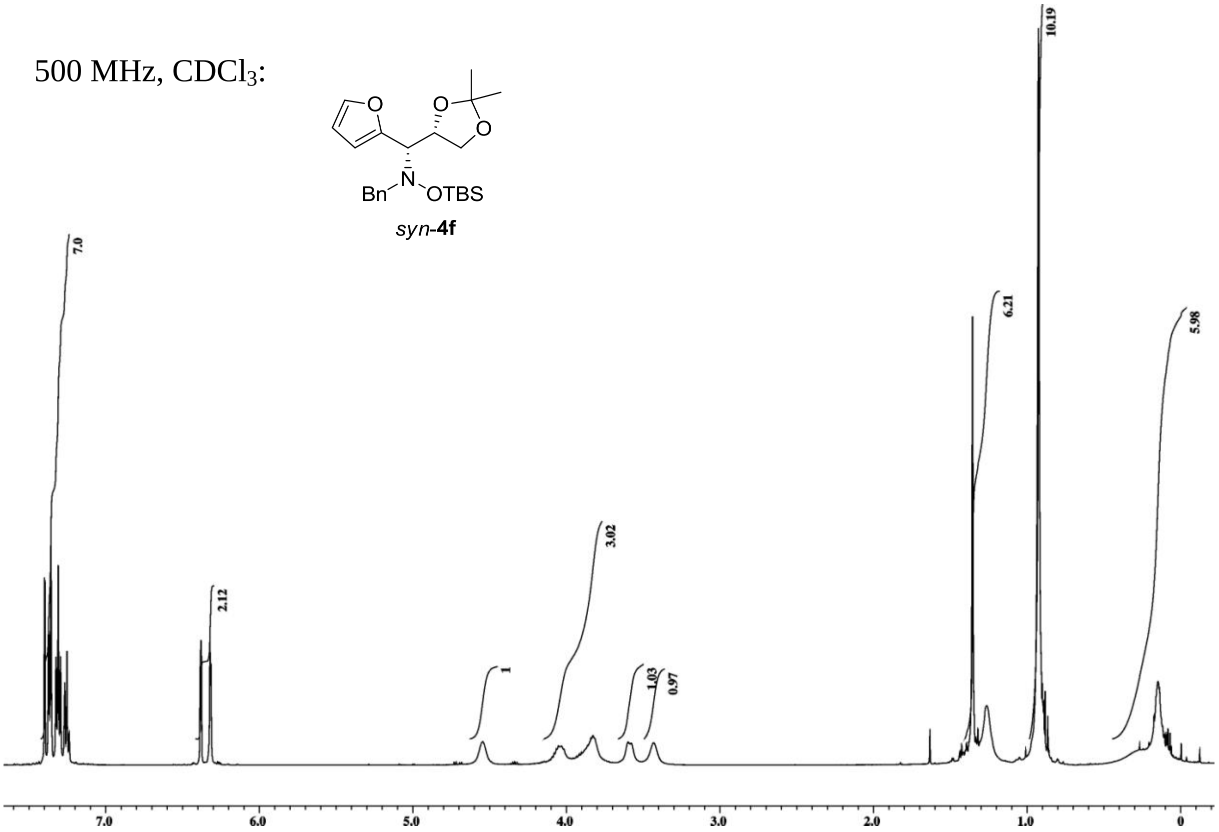

126 MHz, $CDCl_3$:

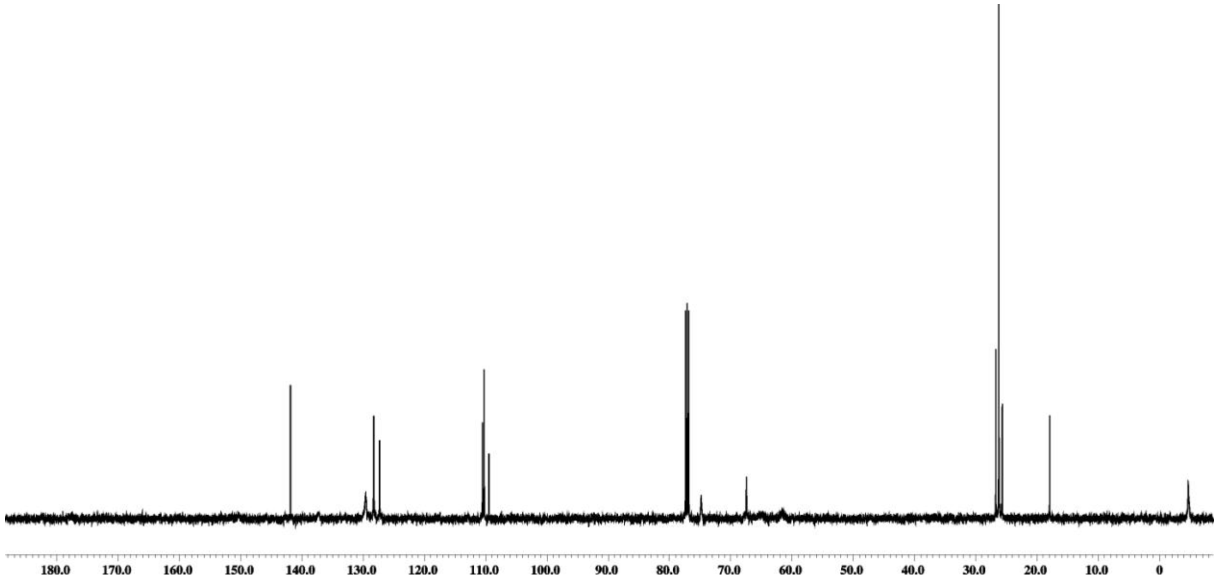

500 MHz, $CDCl_3$:

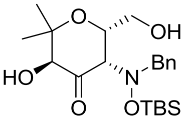

9

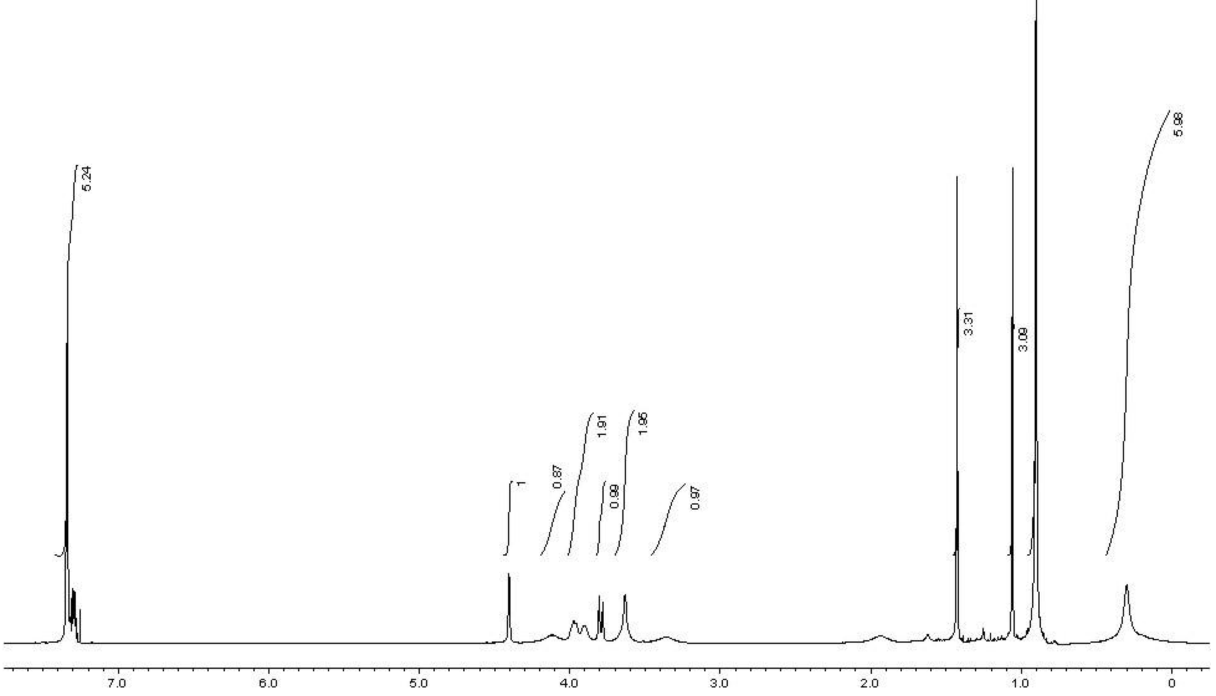

126 MHz, $CDCl_3$:

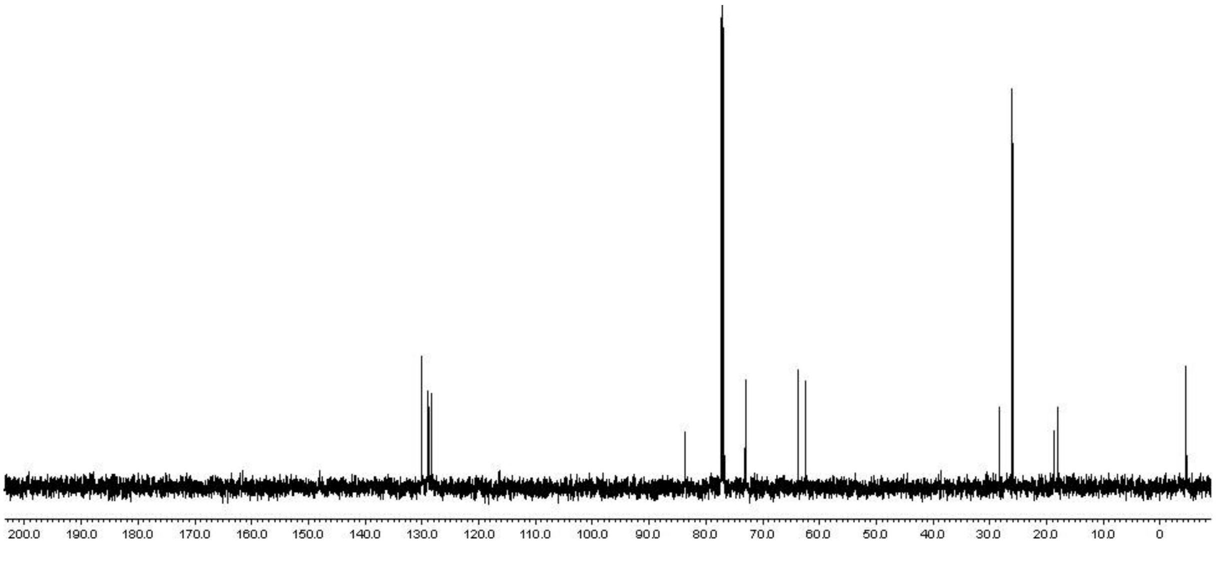

500 MHz, $CDCl_3$:

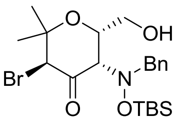

10

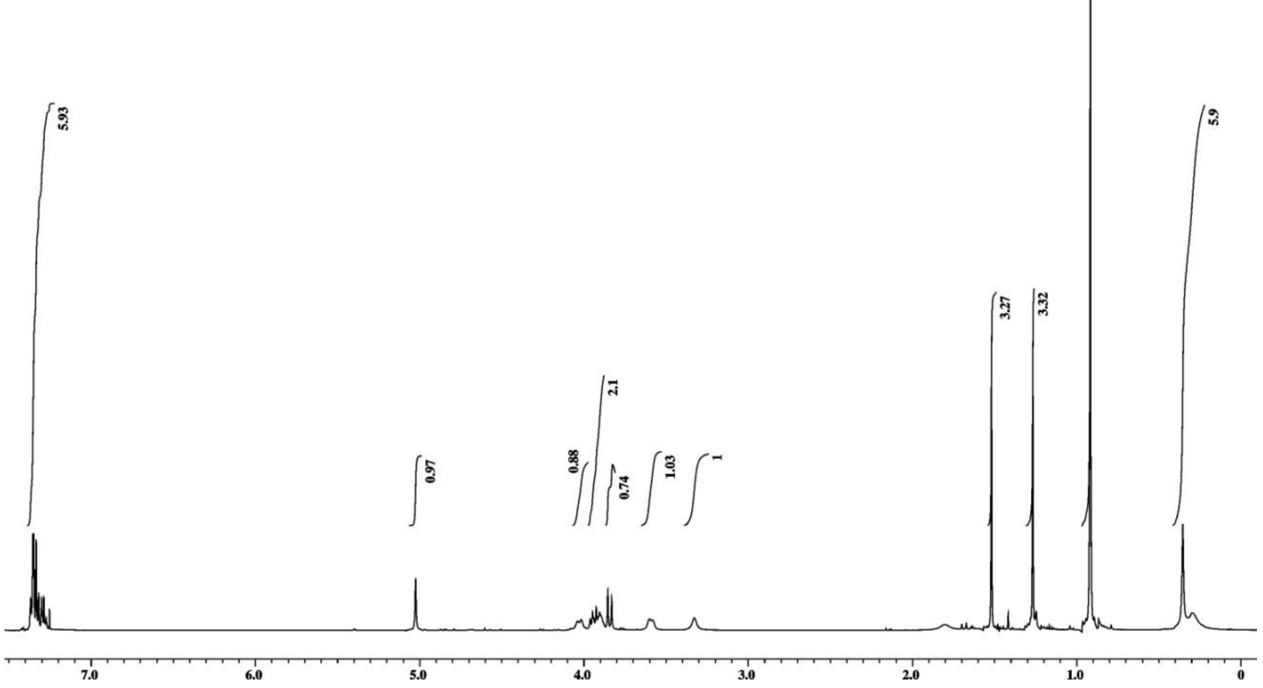

101 MHz, $CDCl_3$:

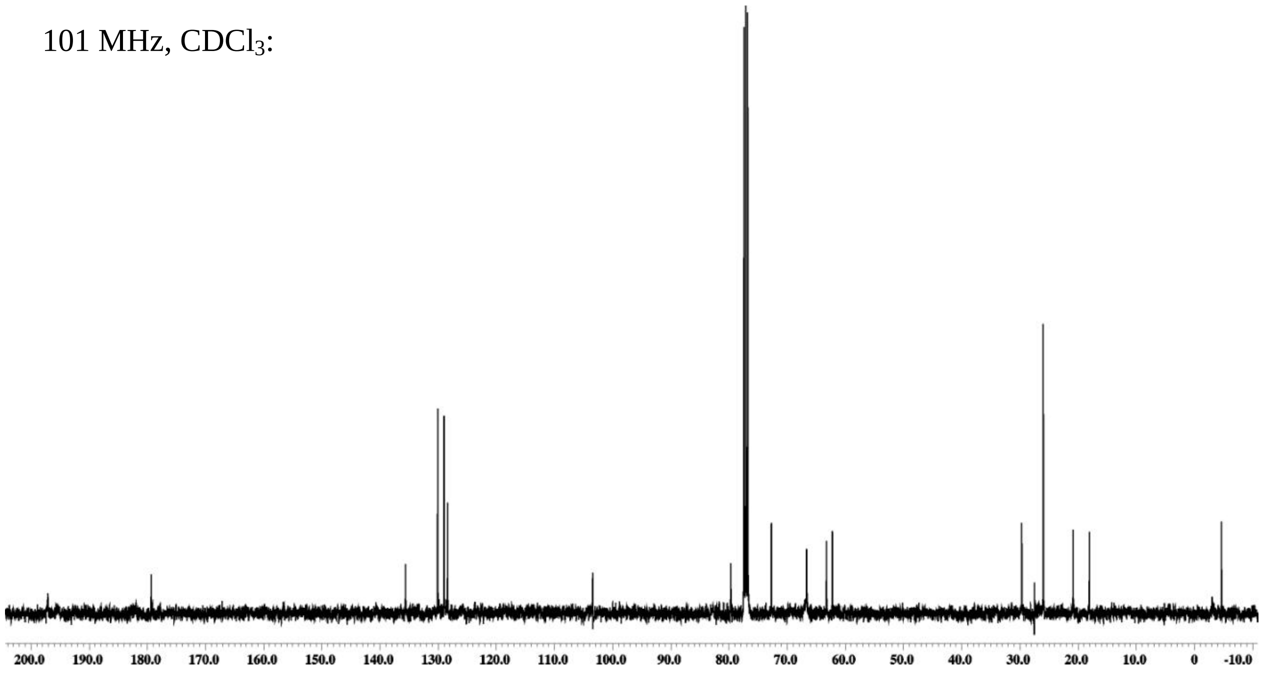

500 MHz, $CDCl_3$:

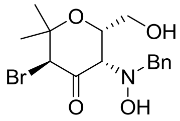

11

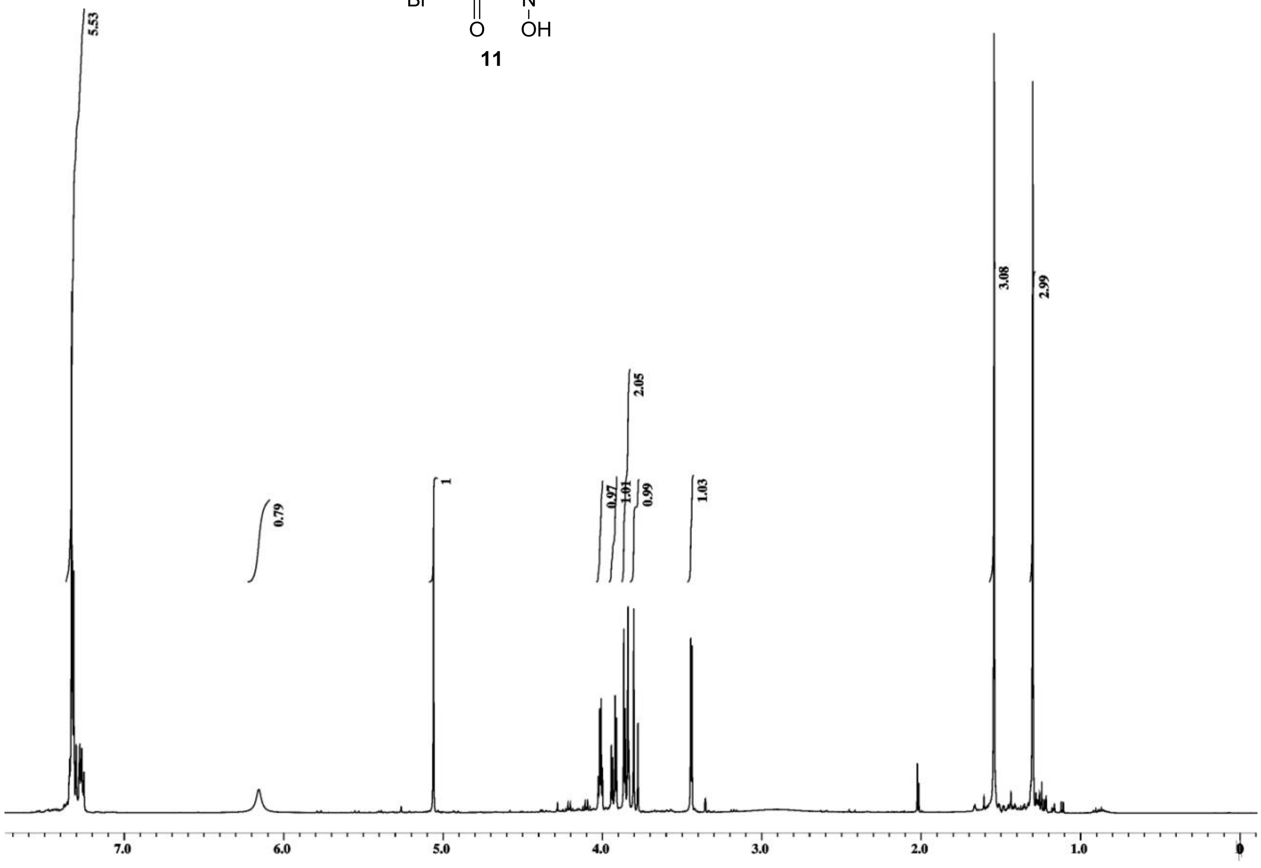

101 MHz, $CDCl_3$:

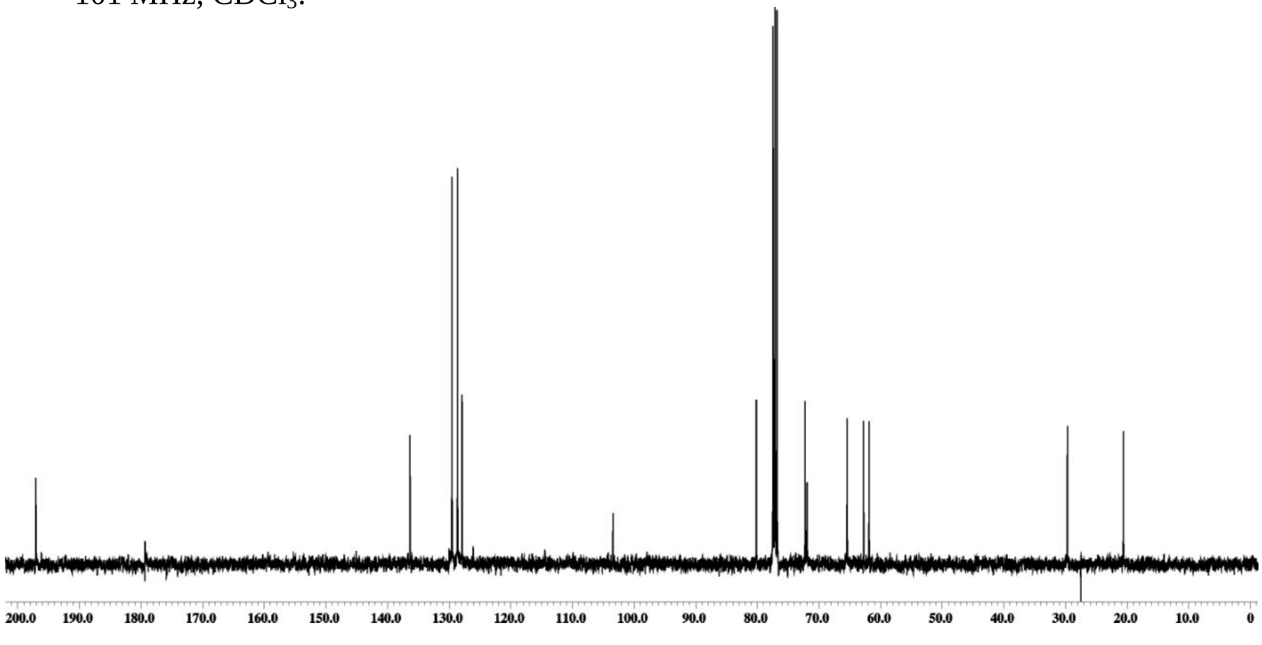

700 MHz, $CDCl_3$:

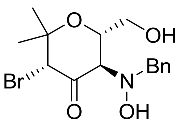

15

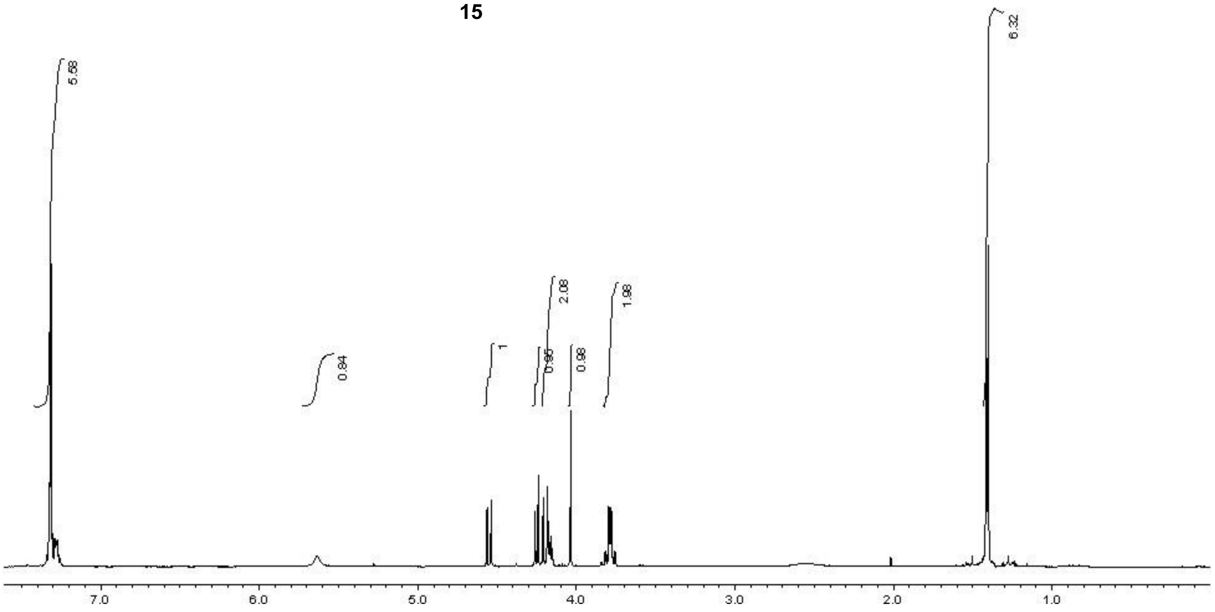

176 MHz, $CDCl_3$:

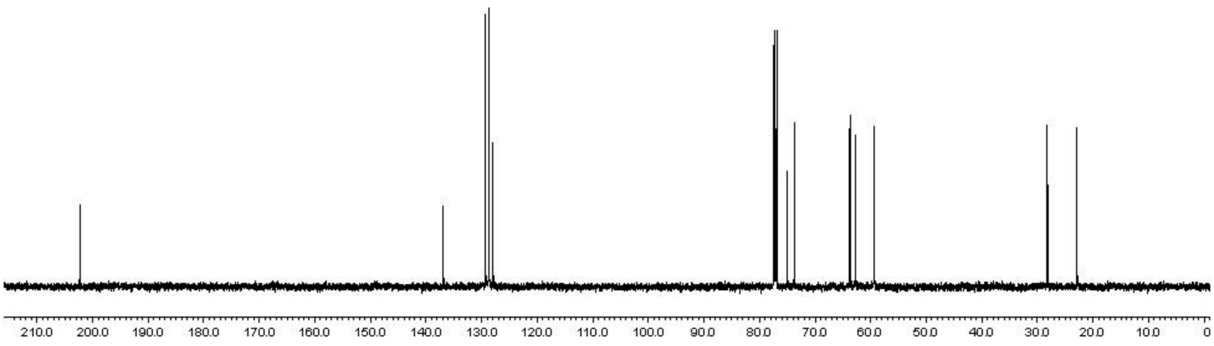

500 MHz, $CDCl_3$:

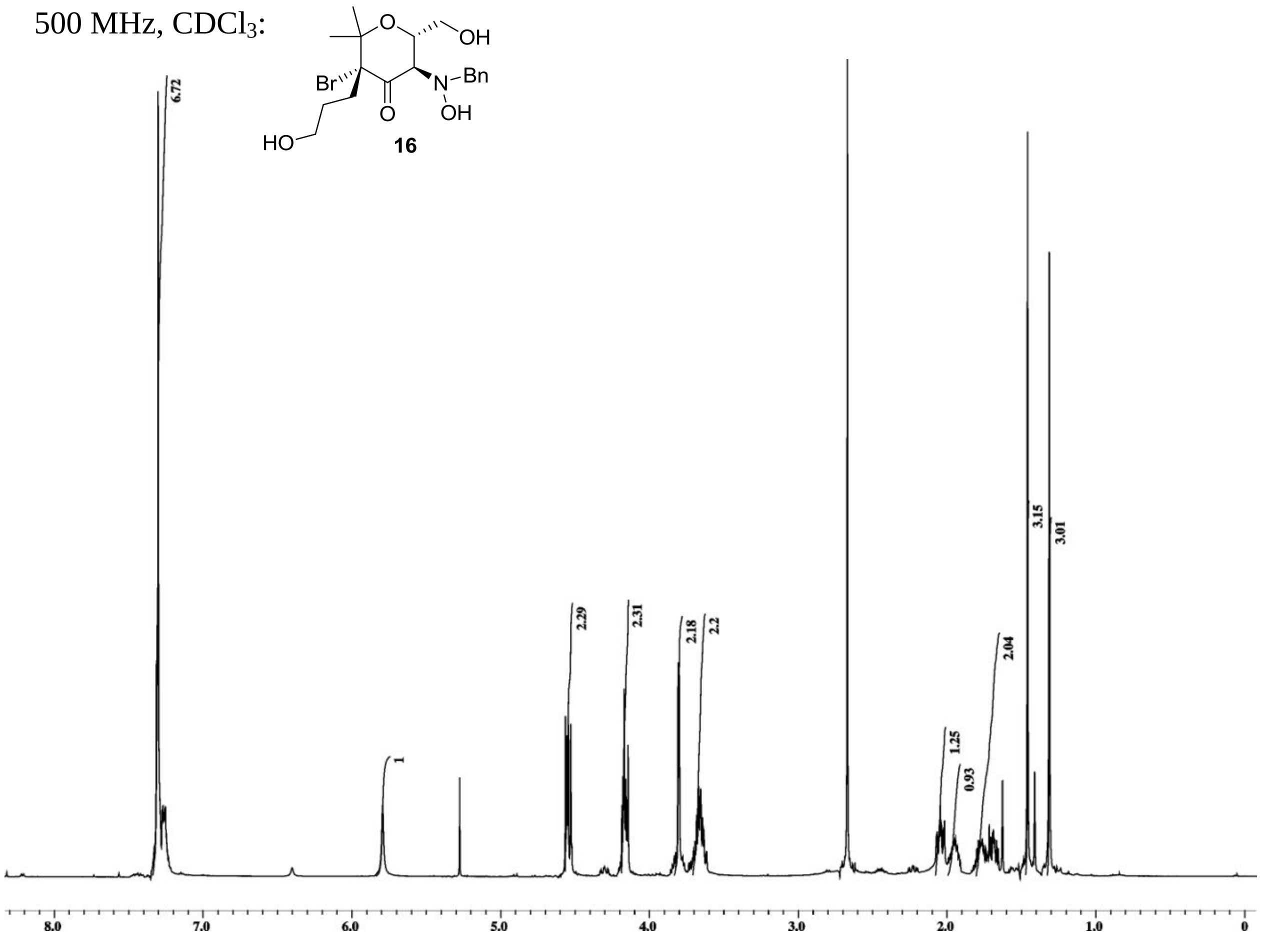

126 MHz, $CDCl_3$:

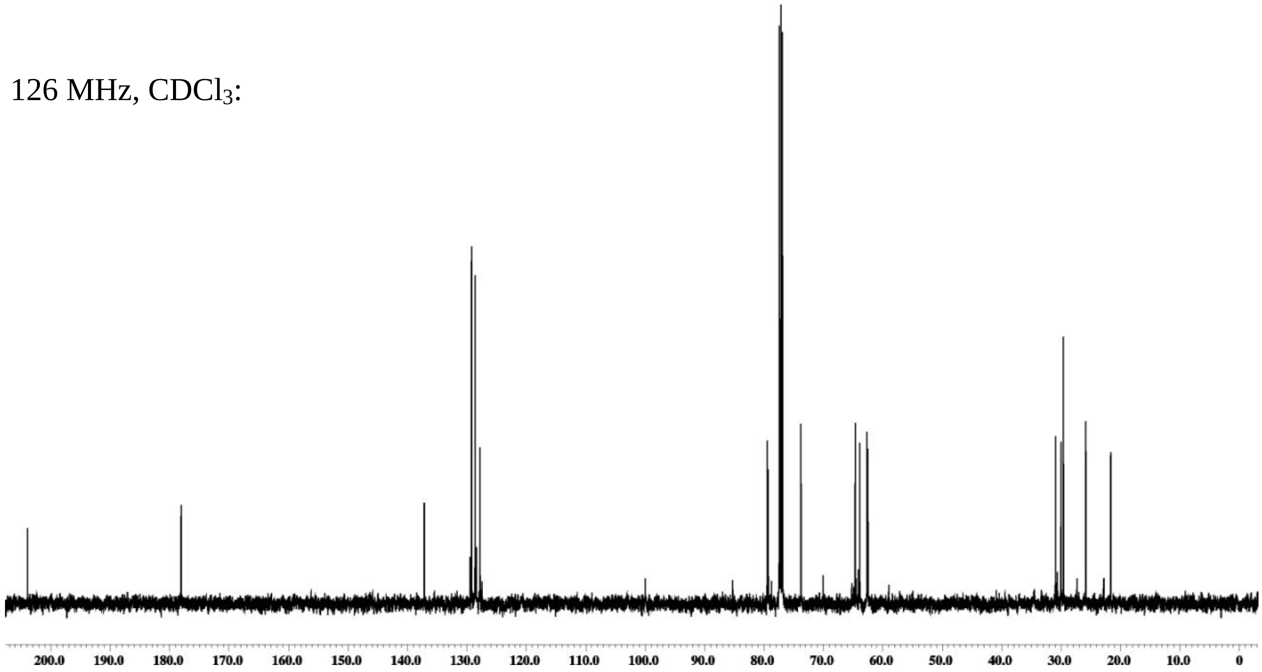

500 MHz, $CDCl_3$:

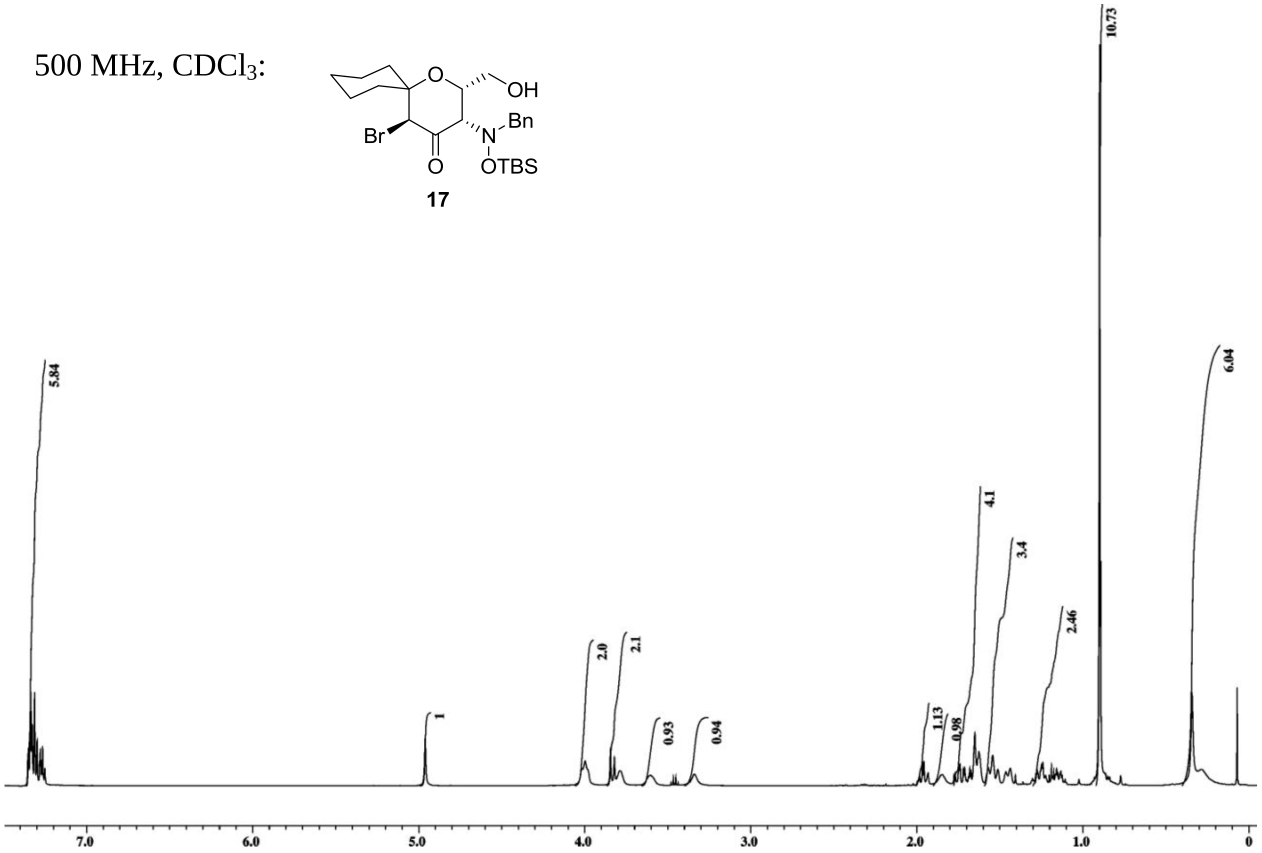

17

126 MHz, $CDCl_3$:

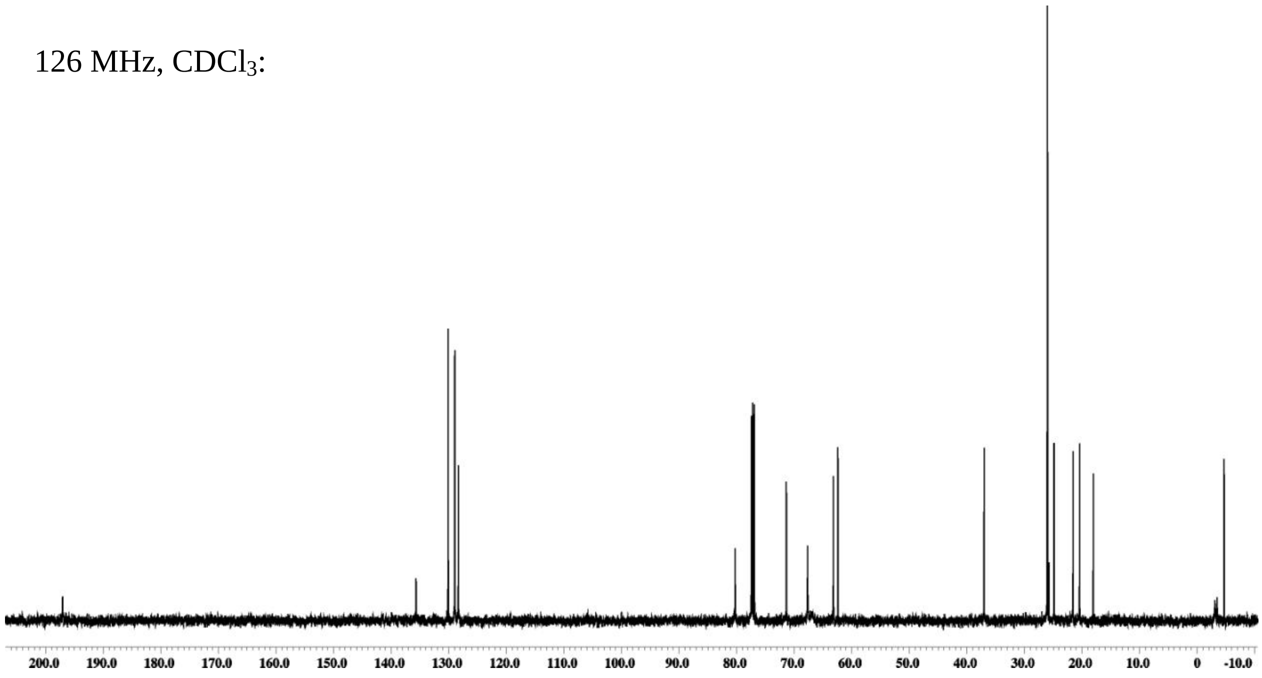

500 MHz, $CDCl_3$:

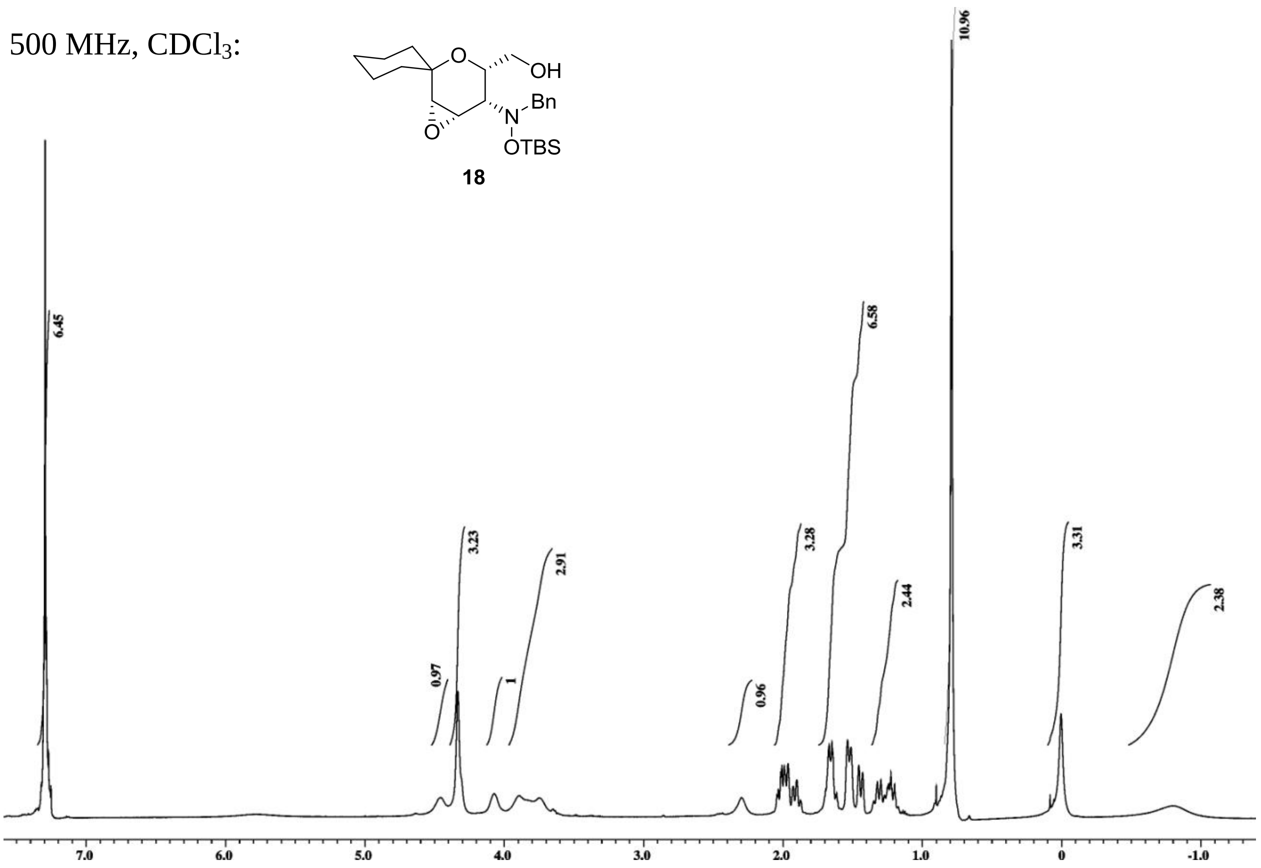

126 MHz, $CDCl_3$:

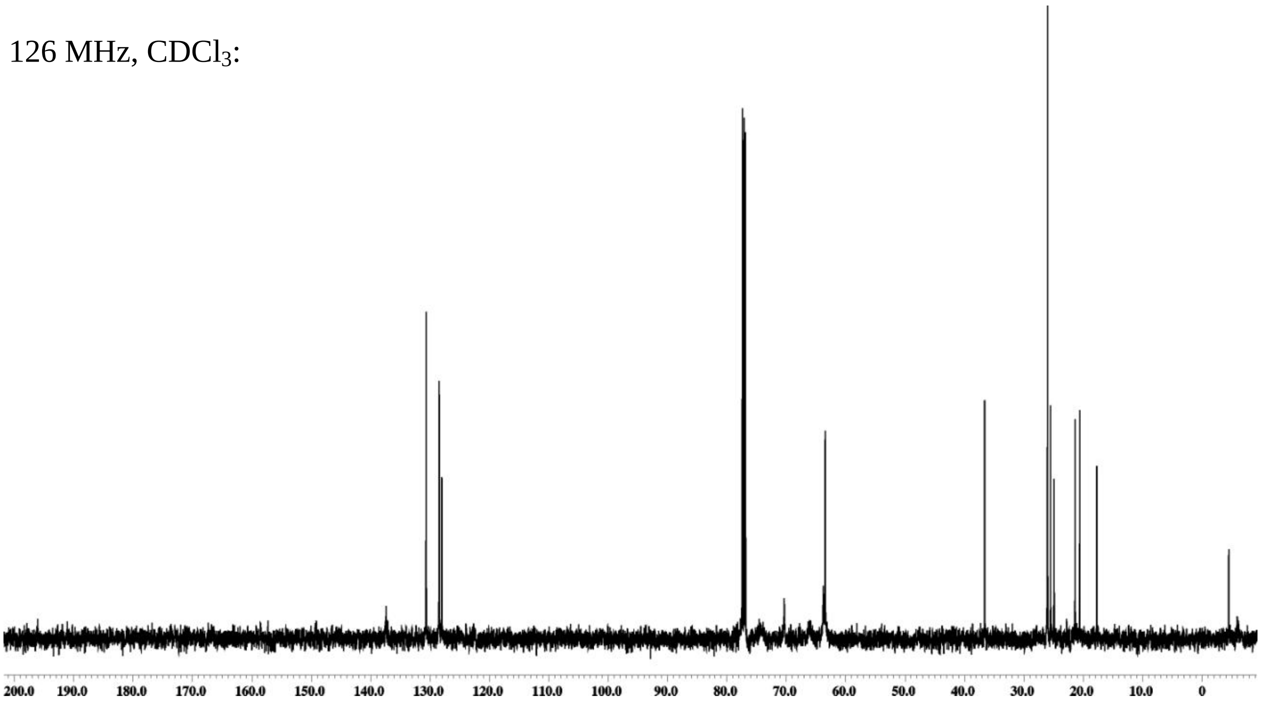

500 MHz, $CDCl_3$:

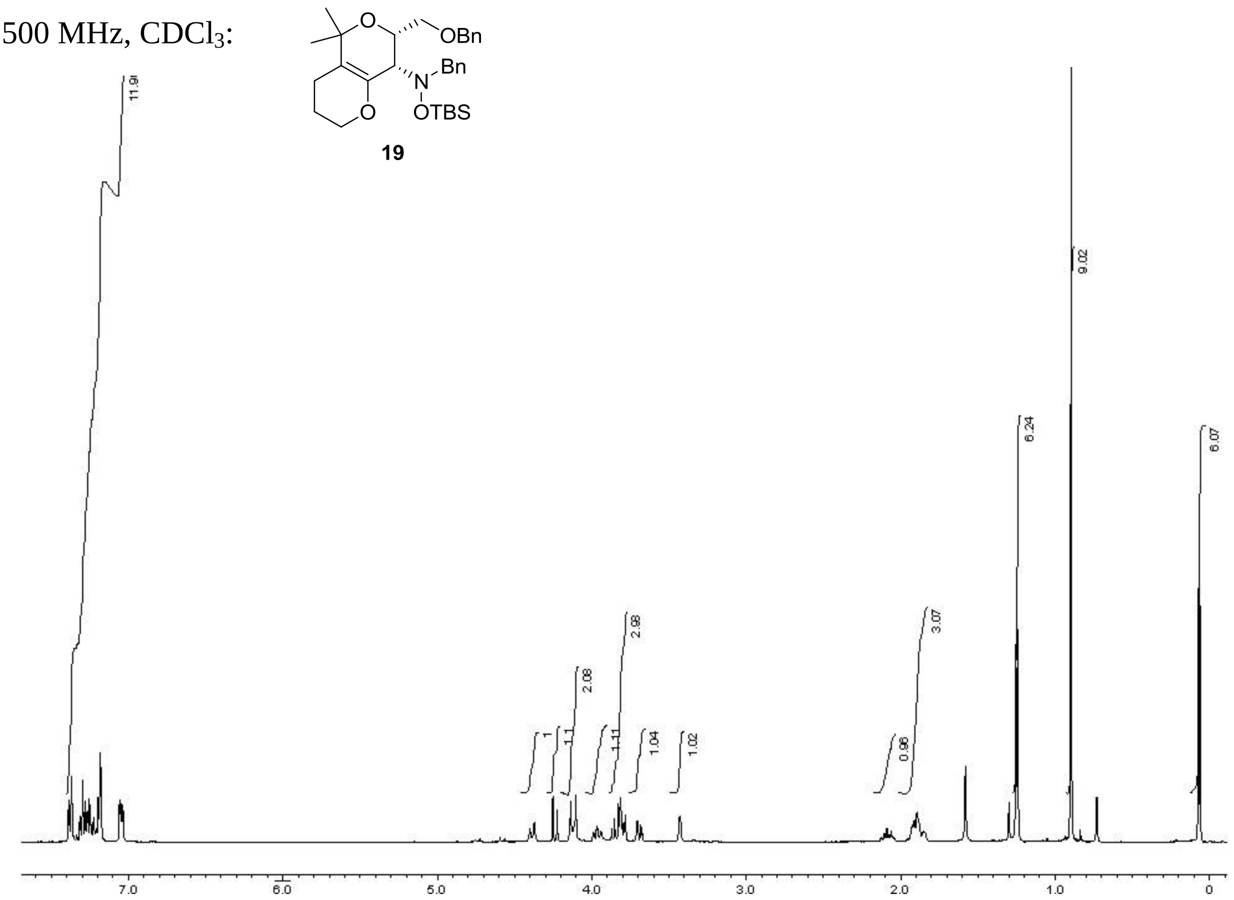

19

126 MHz, $CDCl_3$:

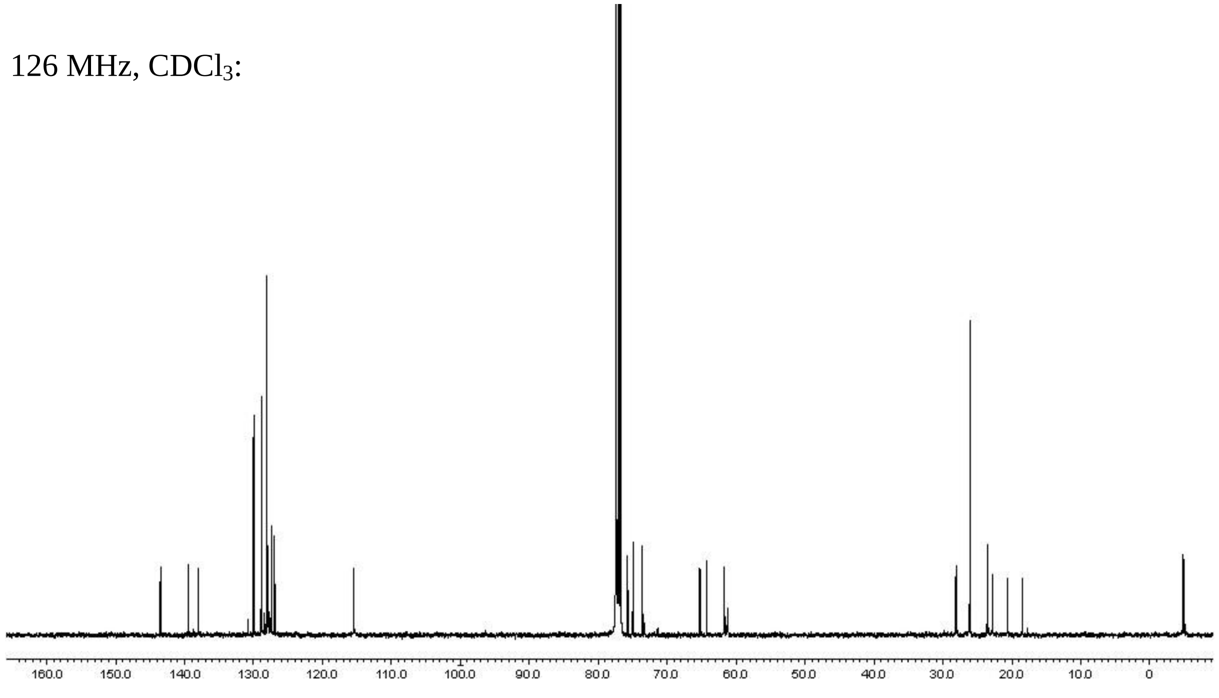

500 MHz, $CDCl_3$:

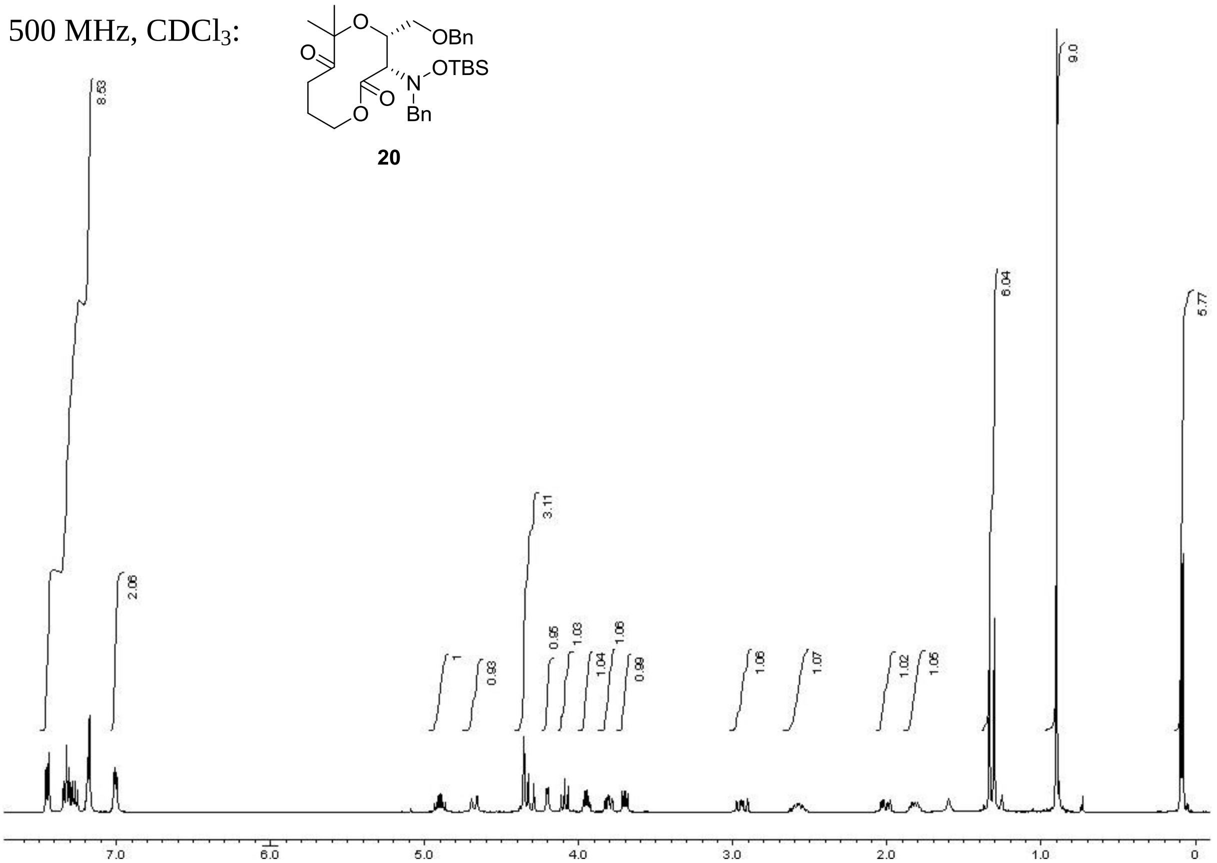

20

101 MHz, $CDCl_3$:

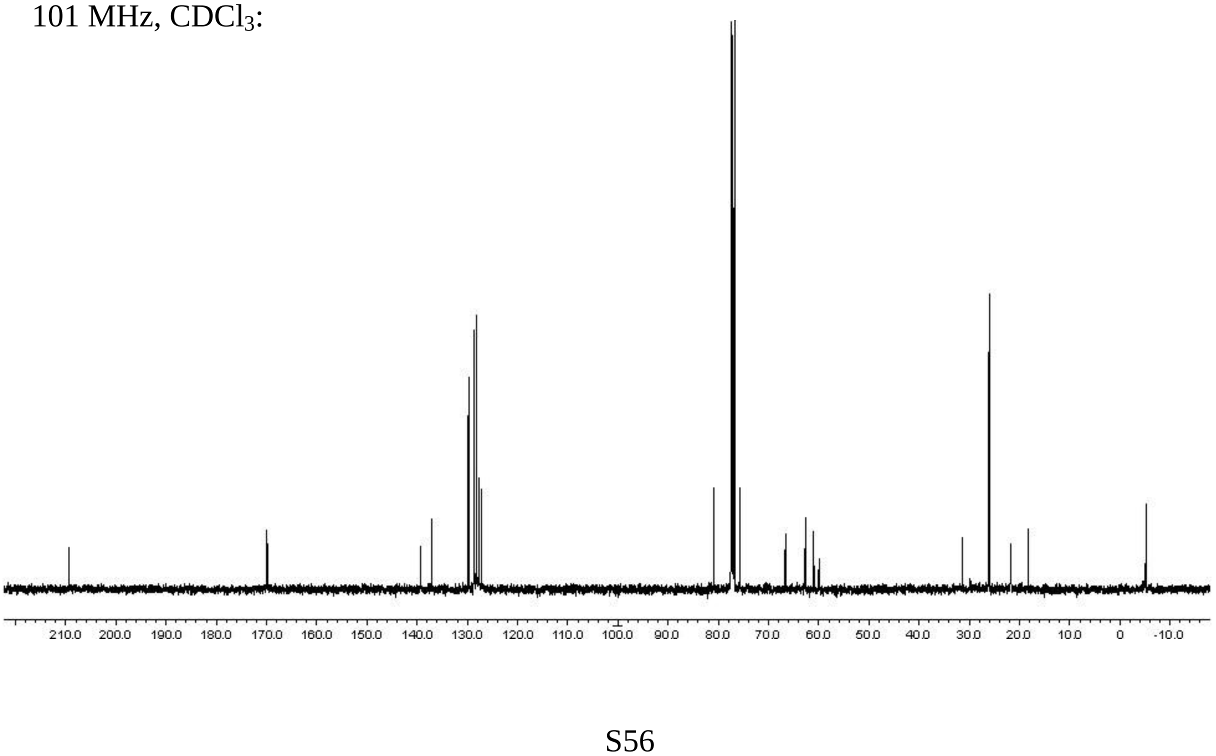

700 MHz, $CDCl_3$:

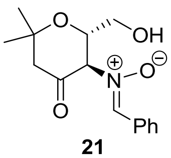

21

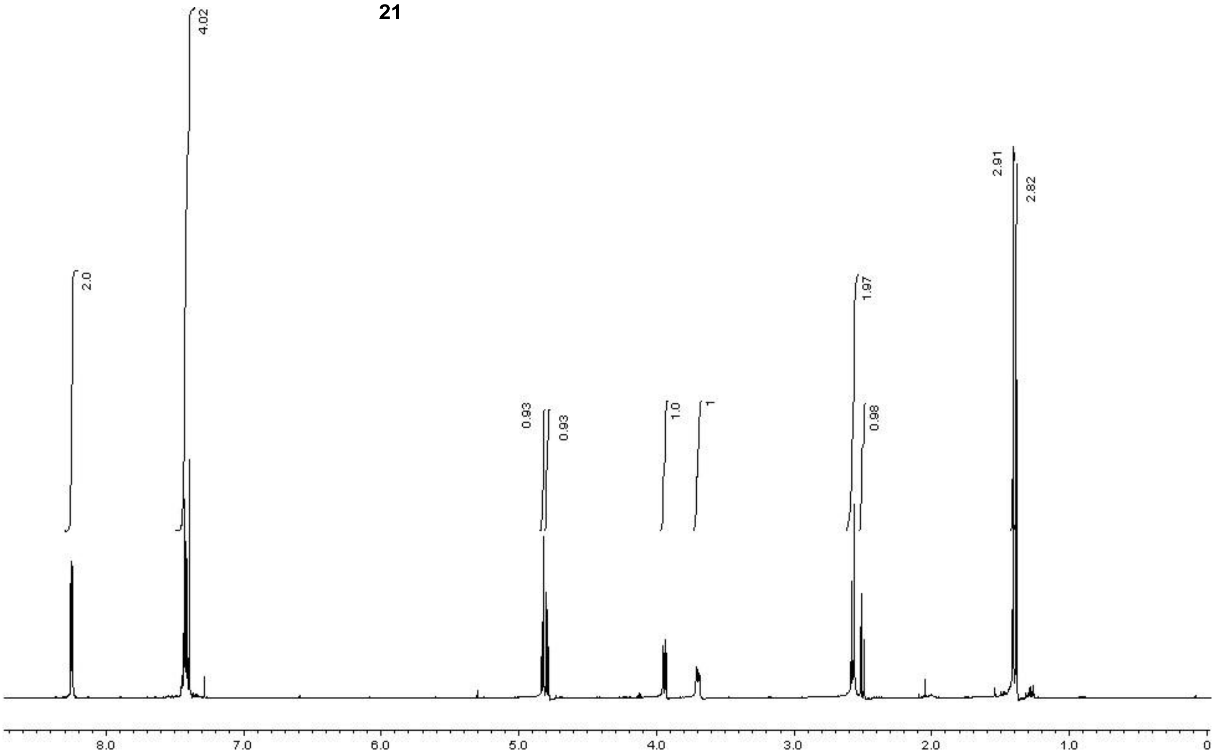

176 MHz, $CDCl_3$:

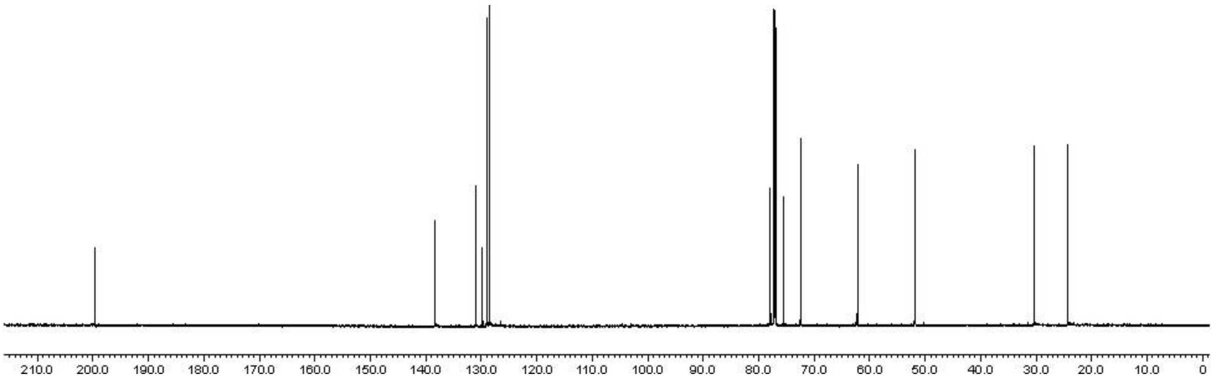

500 MHz, $CDCl_3$:

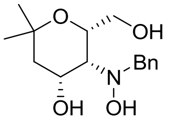

23

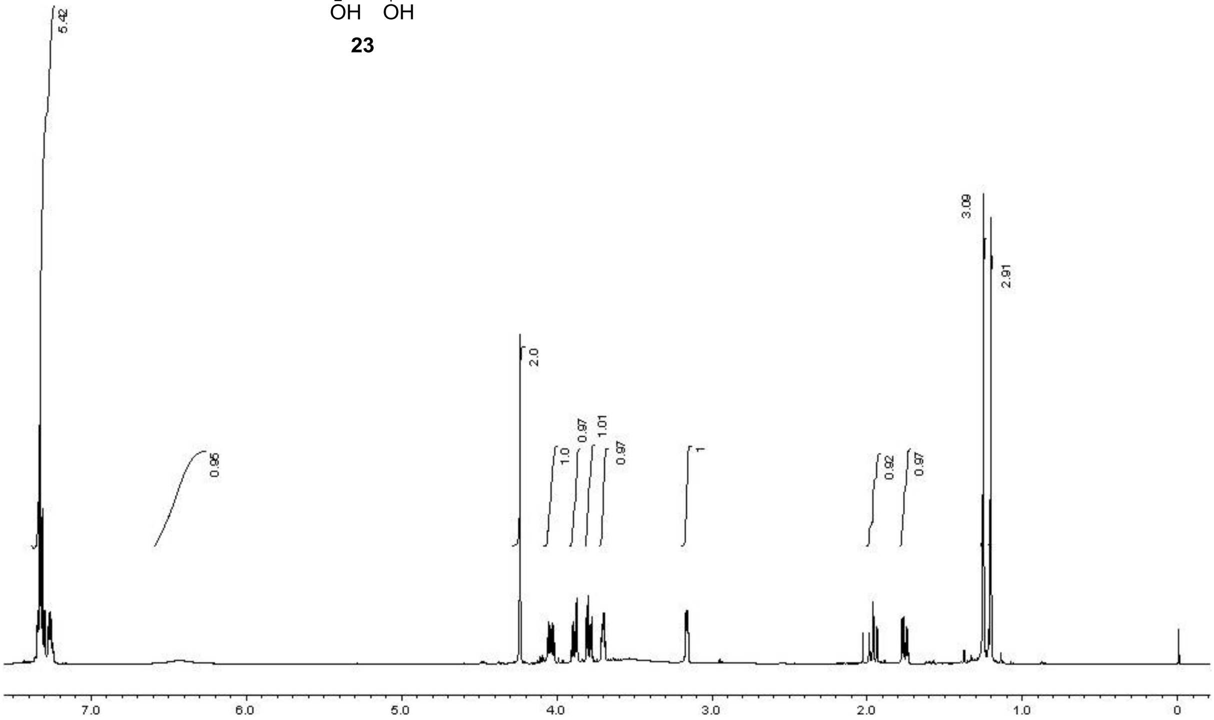

101 MHz, $CDCl_3$:

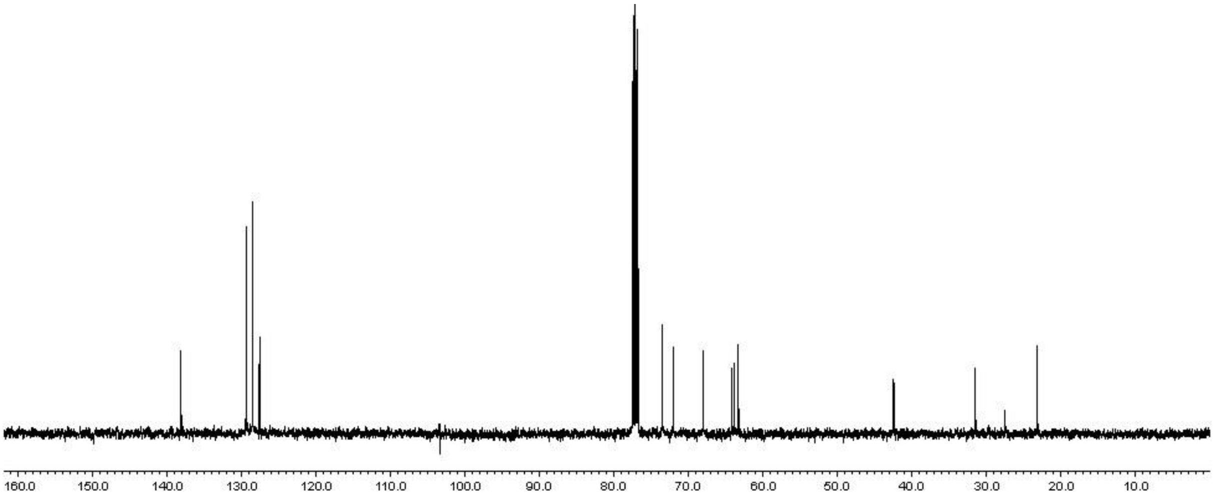

500 MHz, CD_3OD:

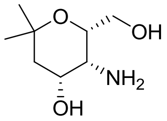

24

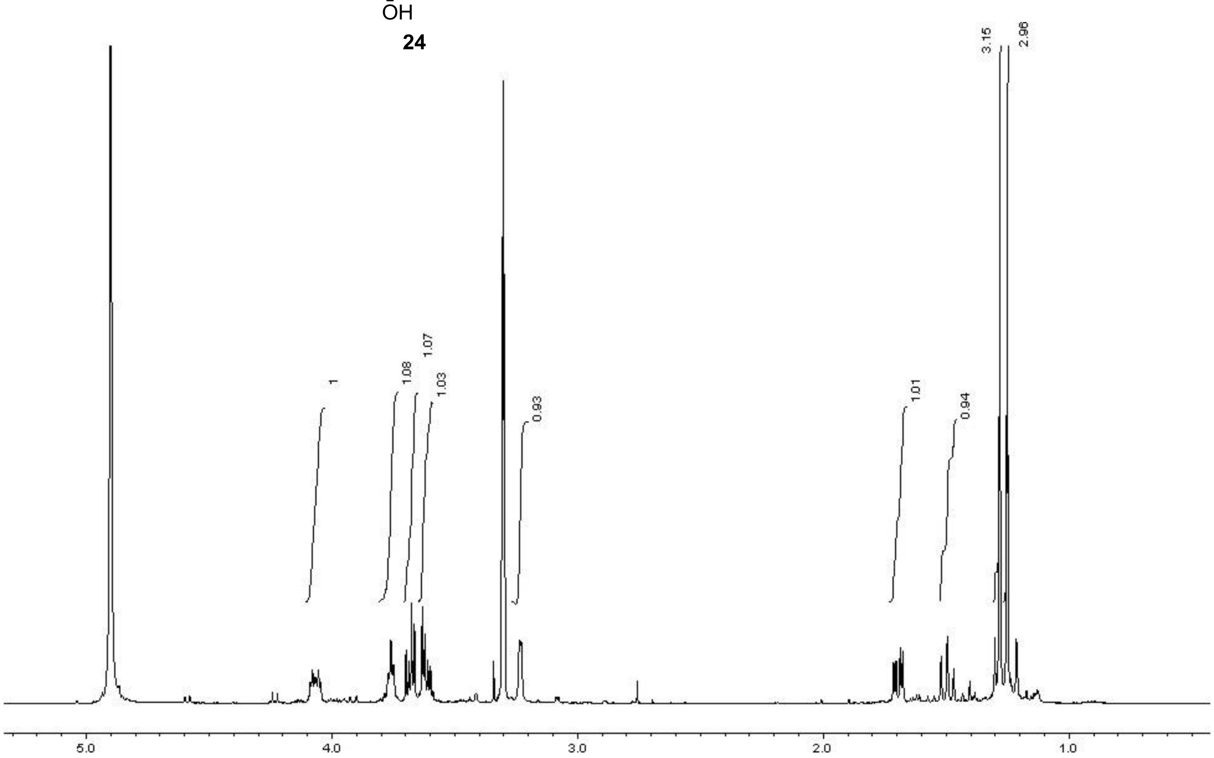

126 MHz, CD_3OD:

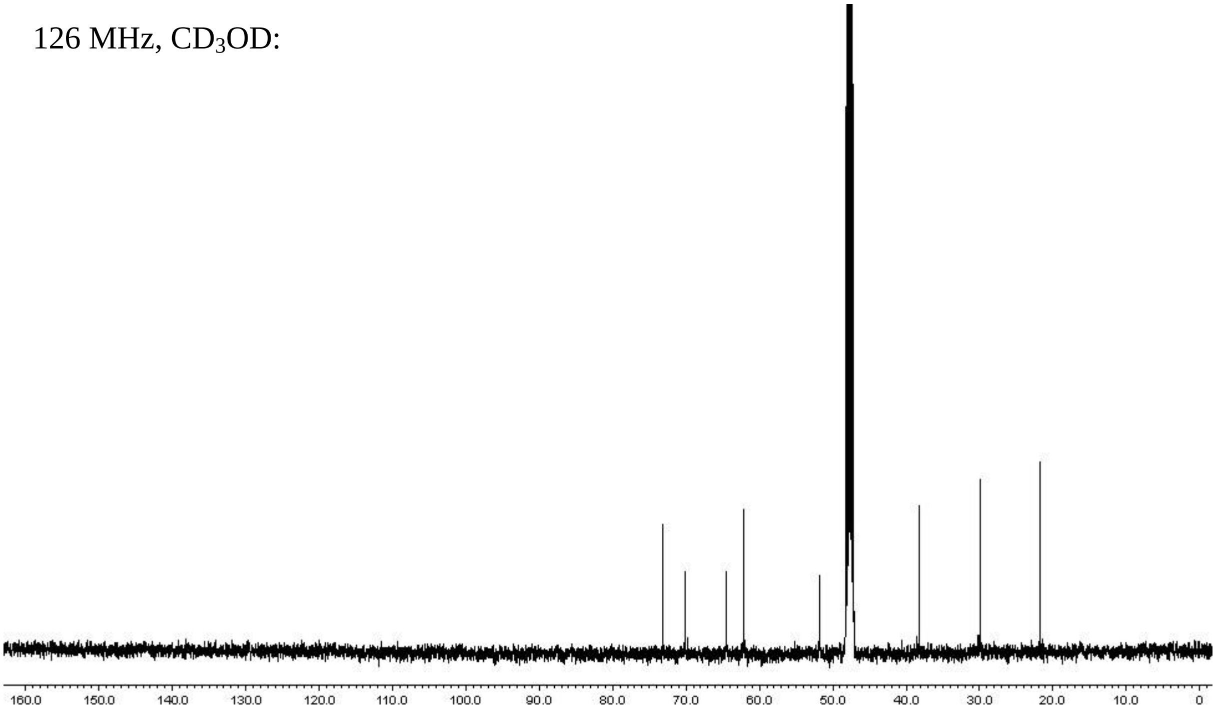

700 MHz, $CDCl_3$:

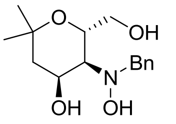

25

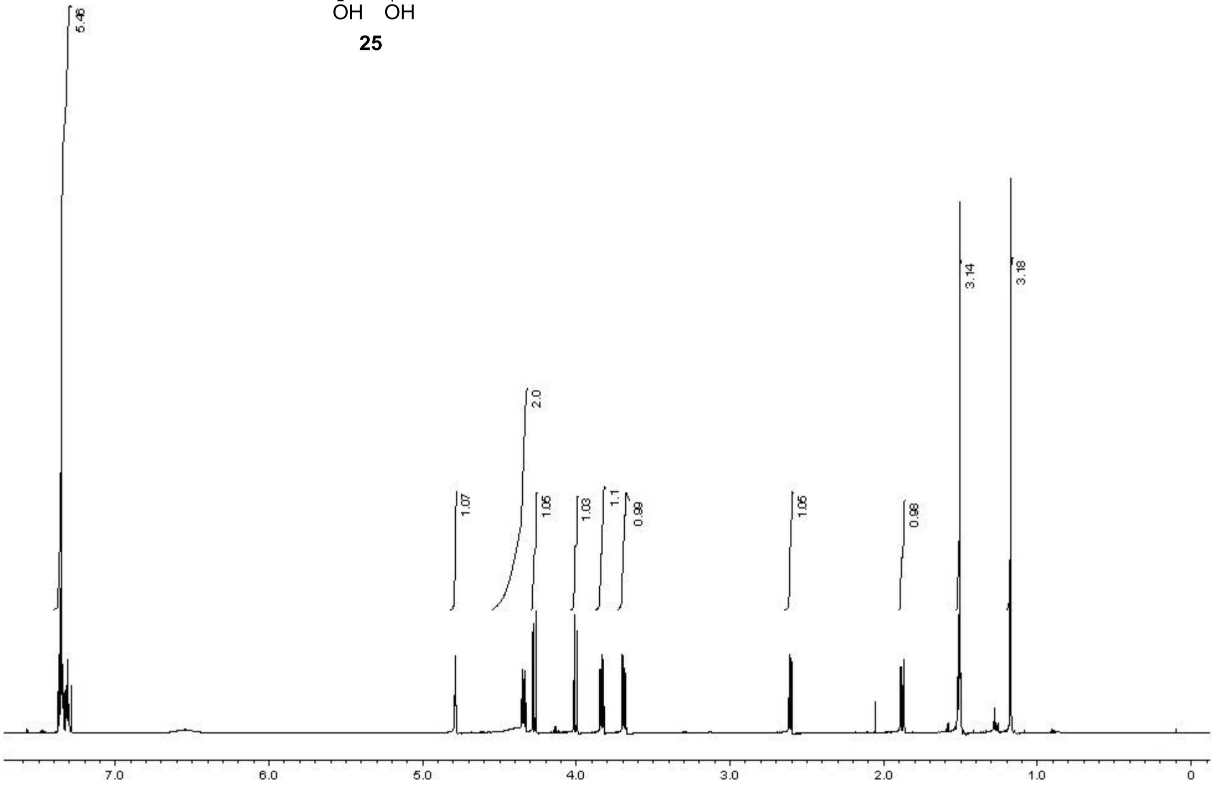

176 MHz, $CDCl_3$:

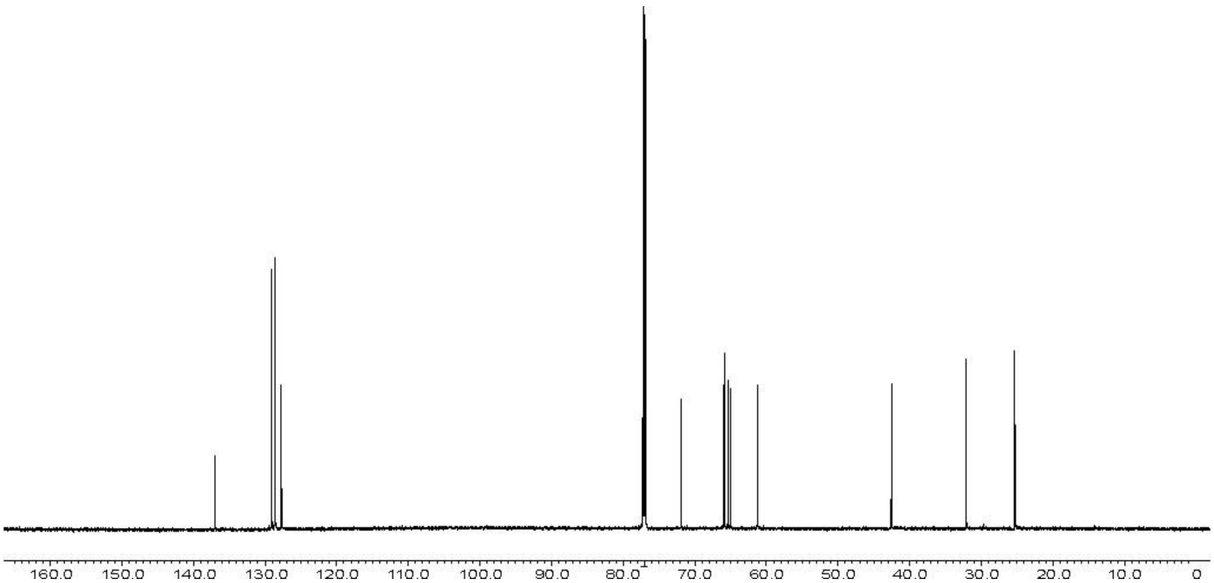

700 MHz, CD_3OD:

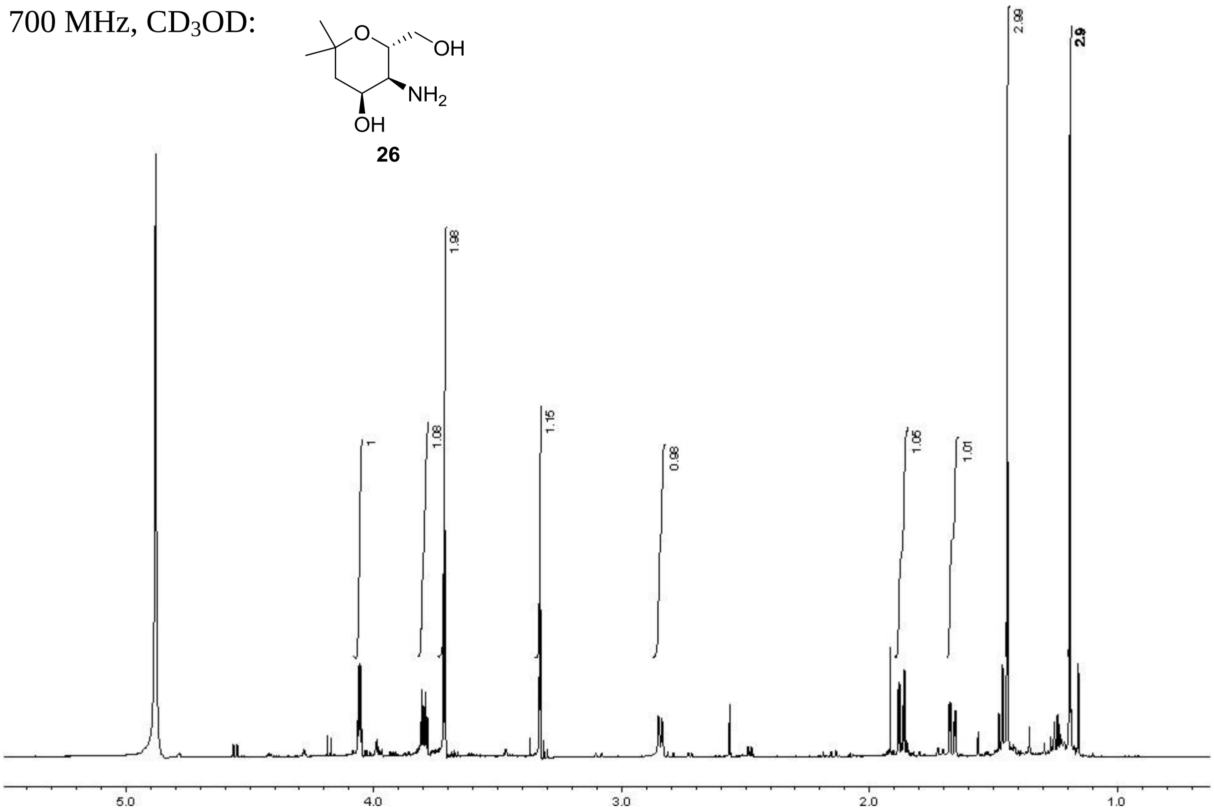

176 MHz, CD_3OD:

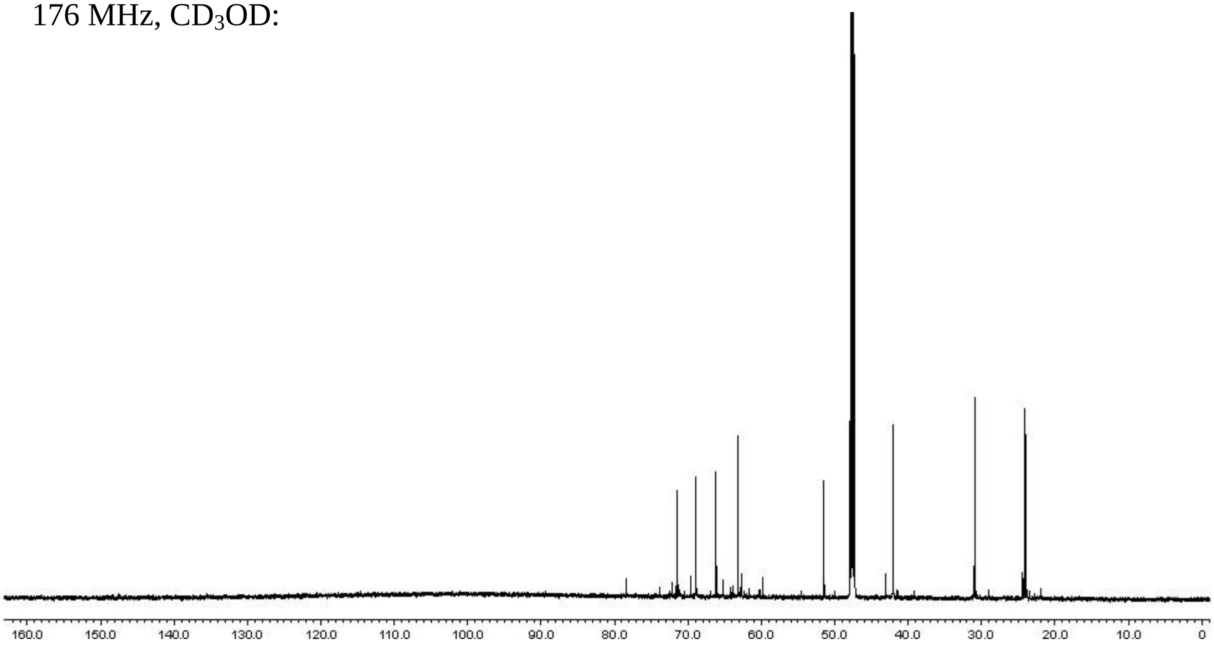

500 MHz, $CDCl_3$:

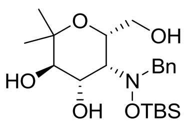

27

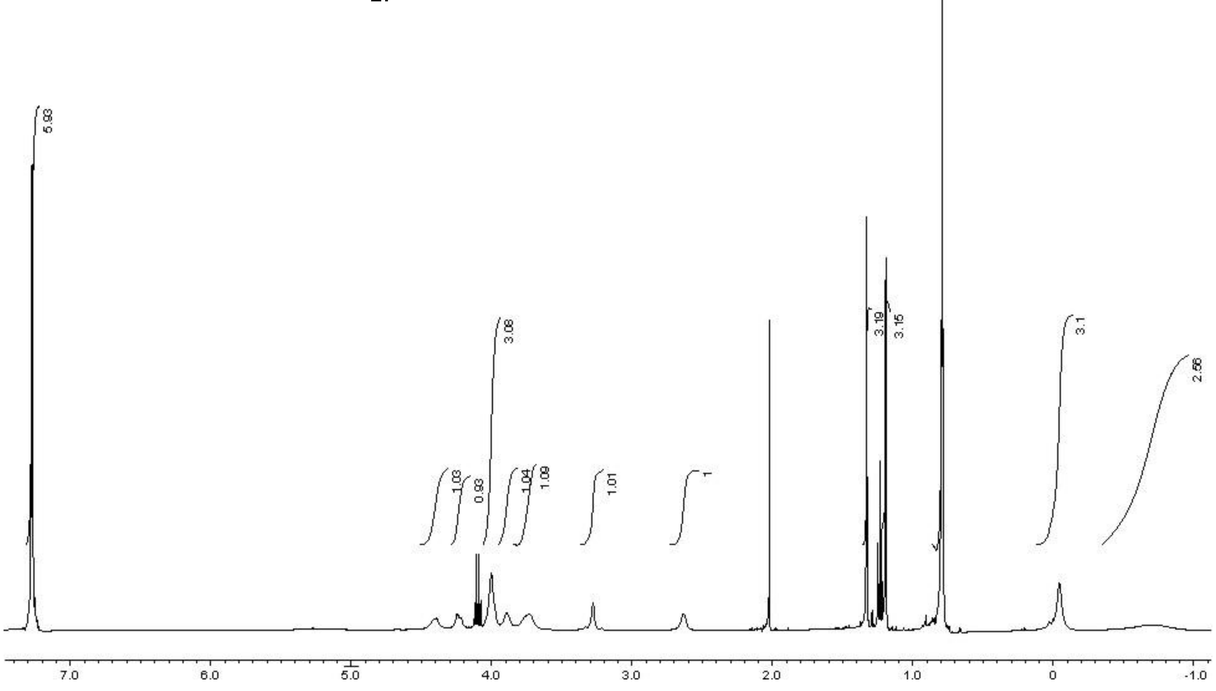

126 MHz, $CDCl_3$:

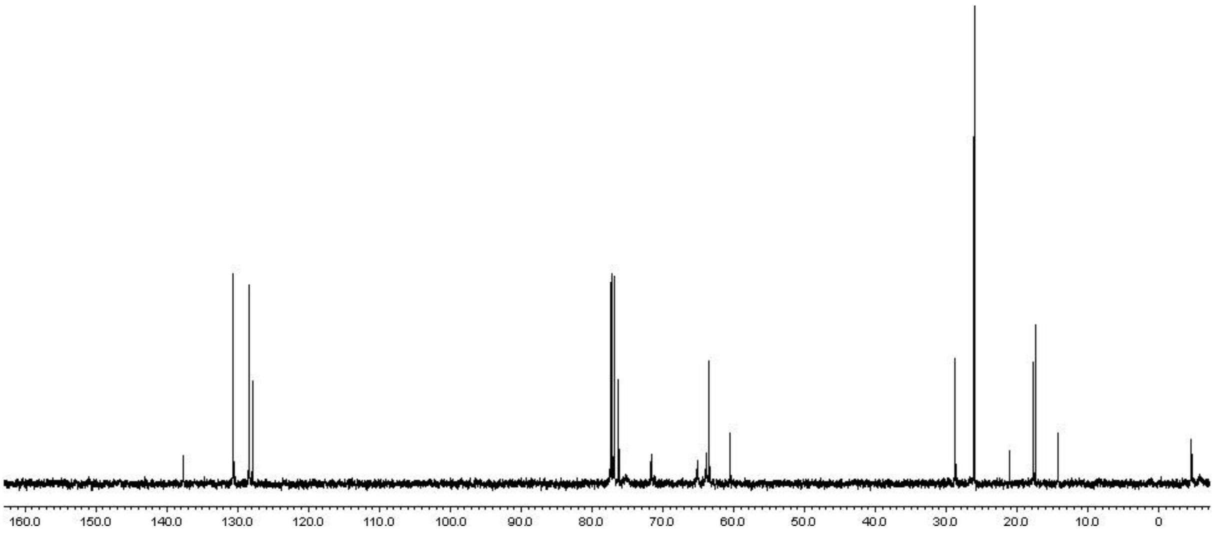

500 MHz, D_2O:

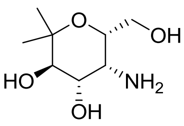

28

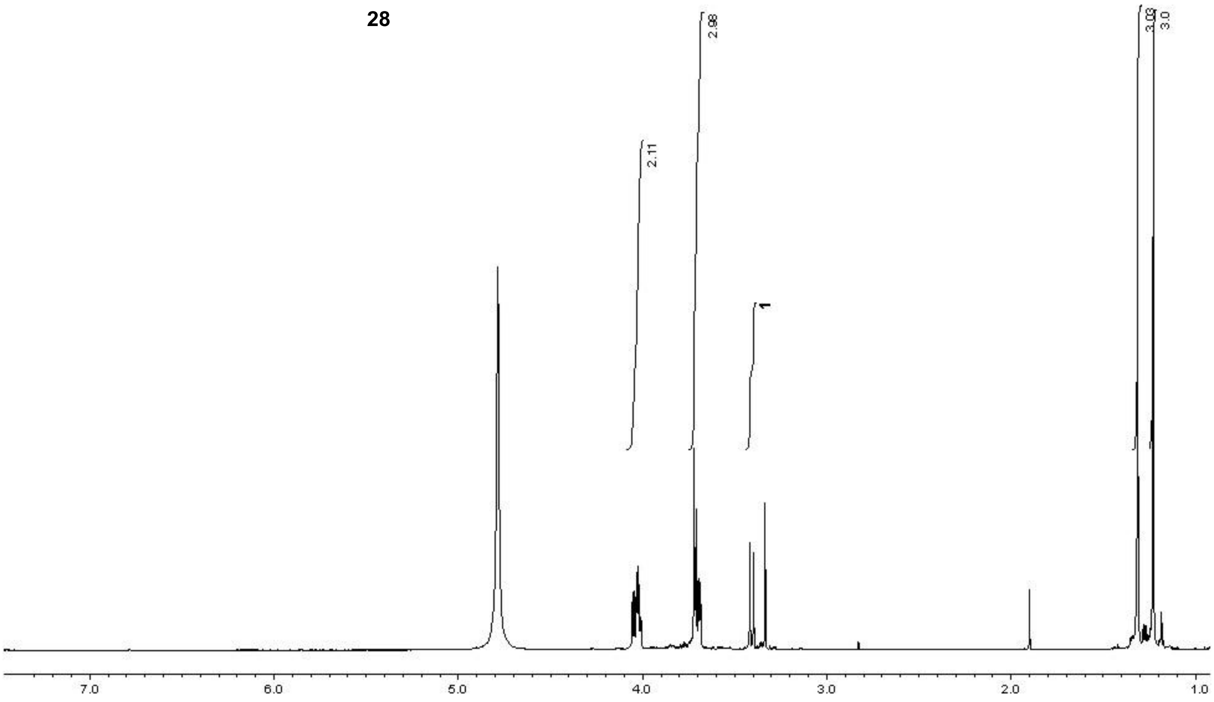

126 MHz, D_2O:

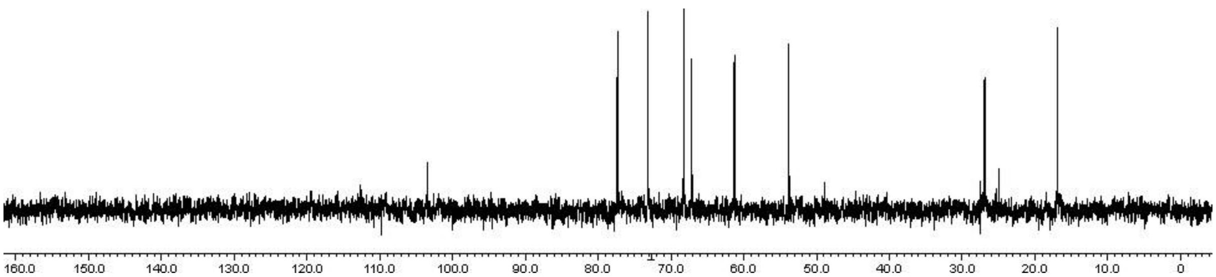

500 MHz, $CDCl_3$:

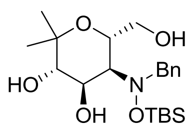

29

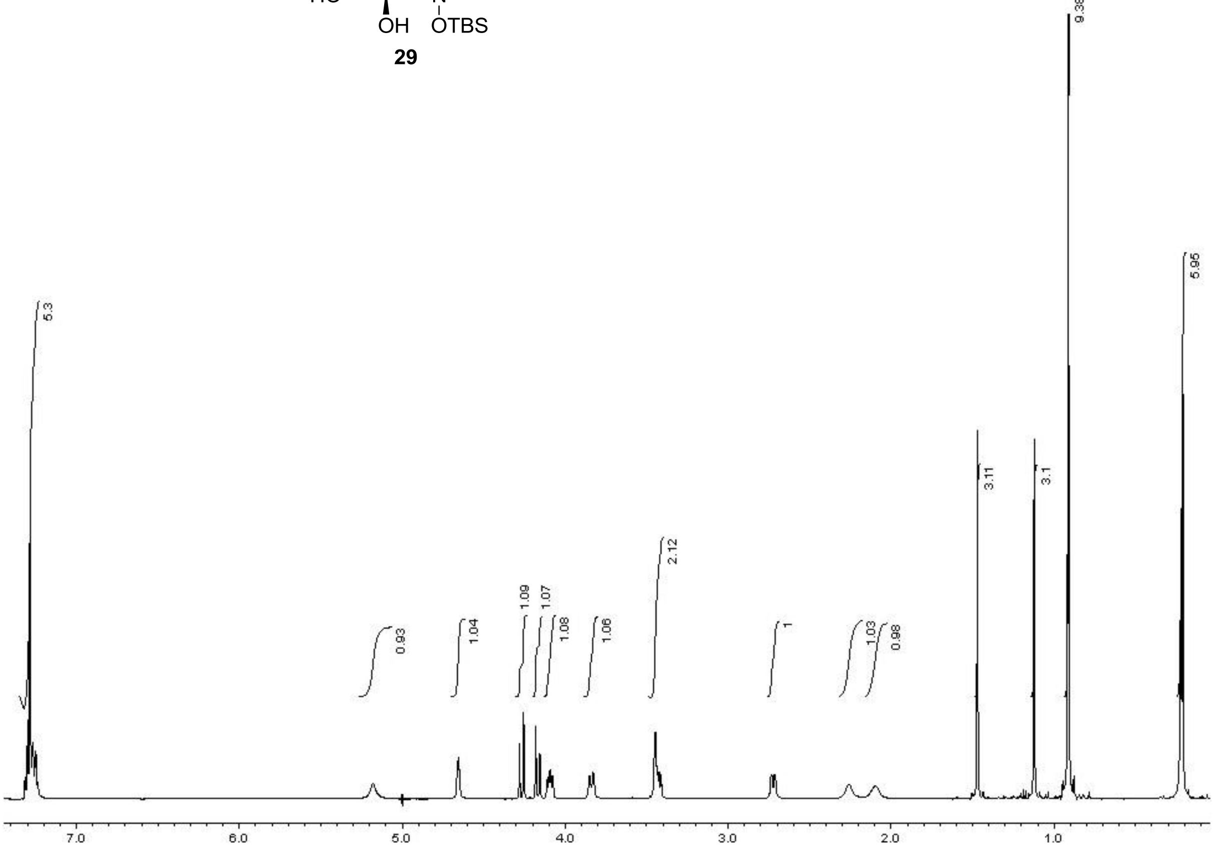

126 MHz, $CDCl_3$:

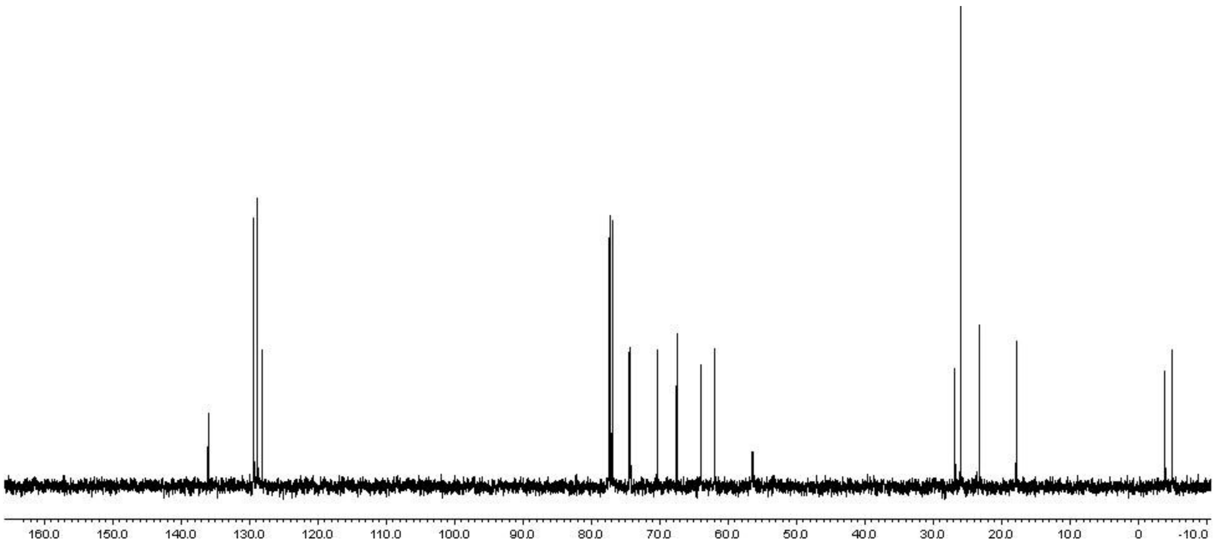

400 MHz, D_2O:

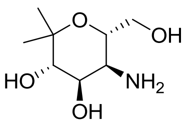

30

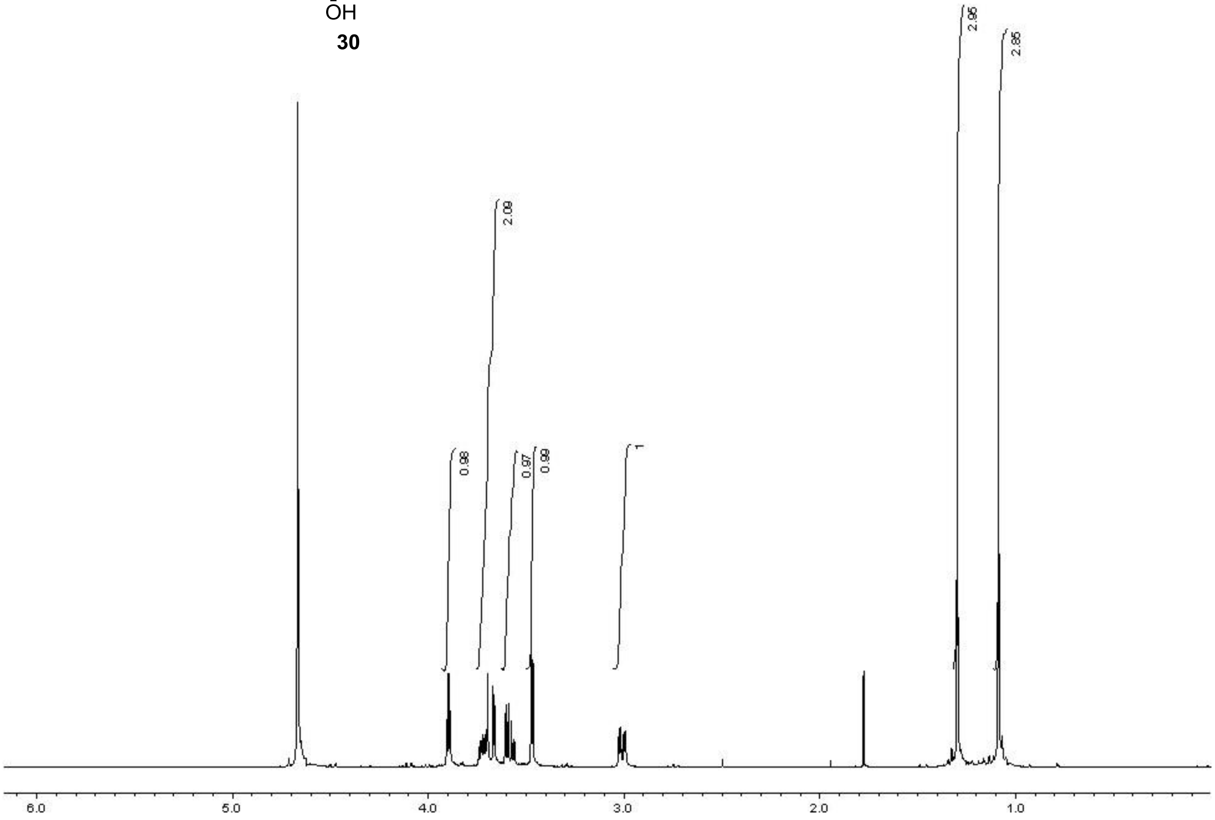

101 MHz, D_2O:

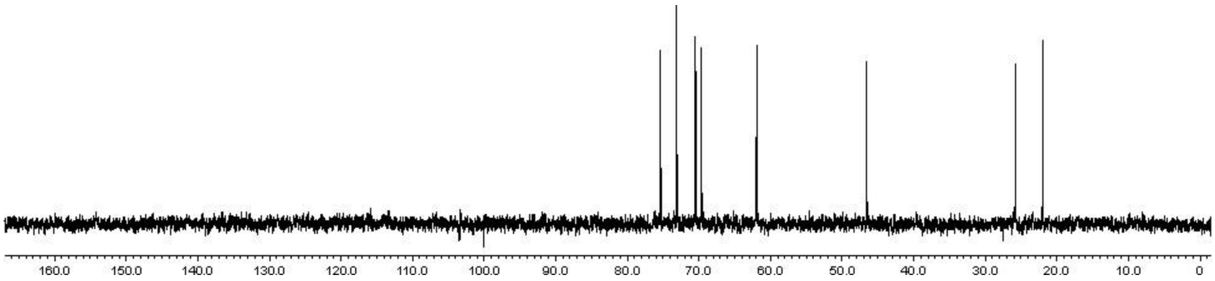

700 MHz, DMF-d_7:

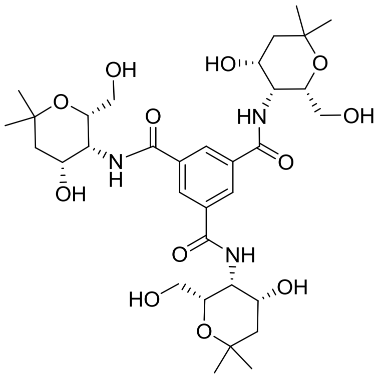

31

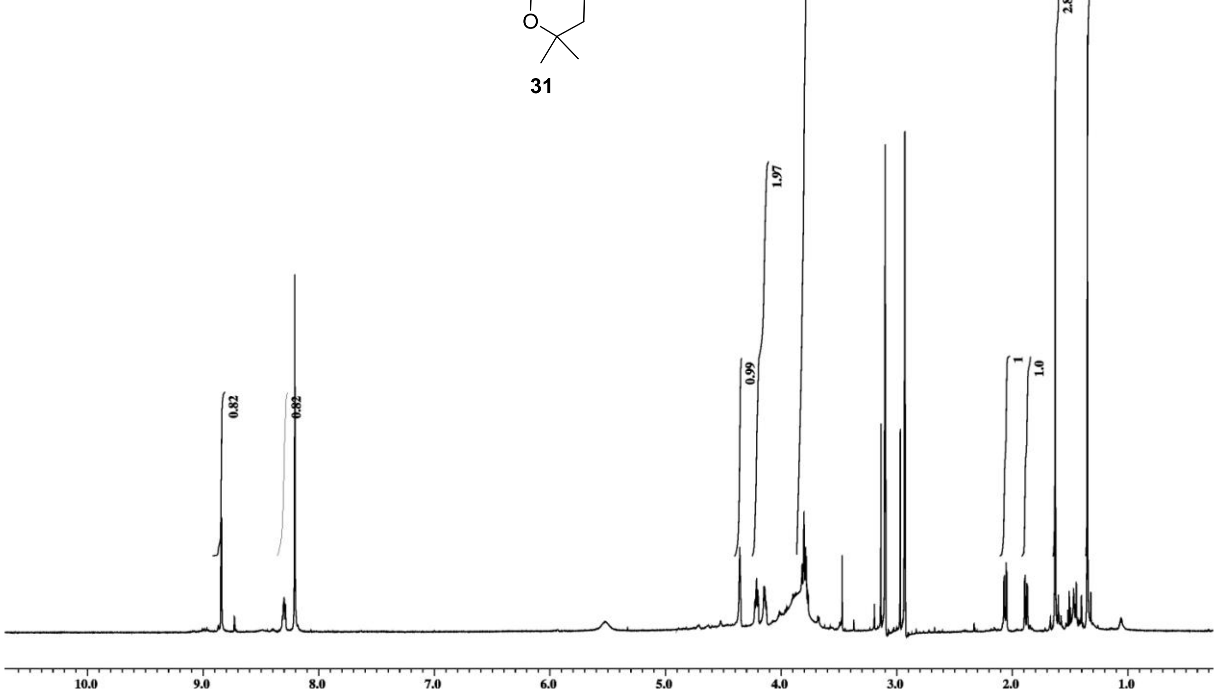

176 MHz, DMF-d_7:

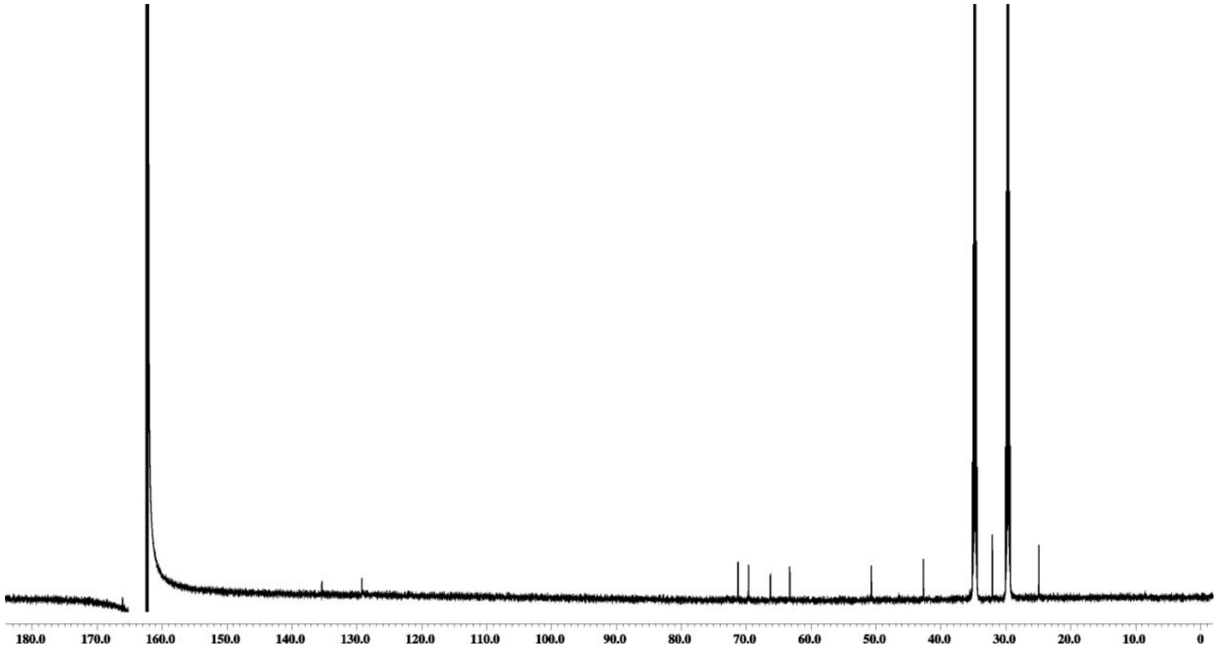

700 MHz, D_2O:

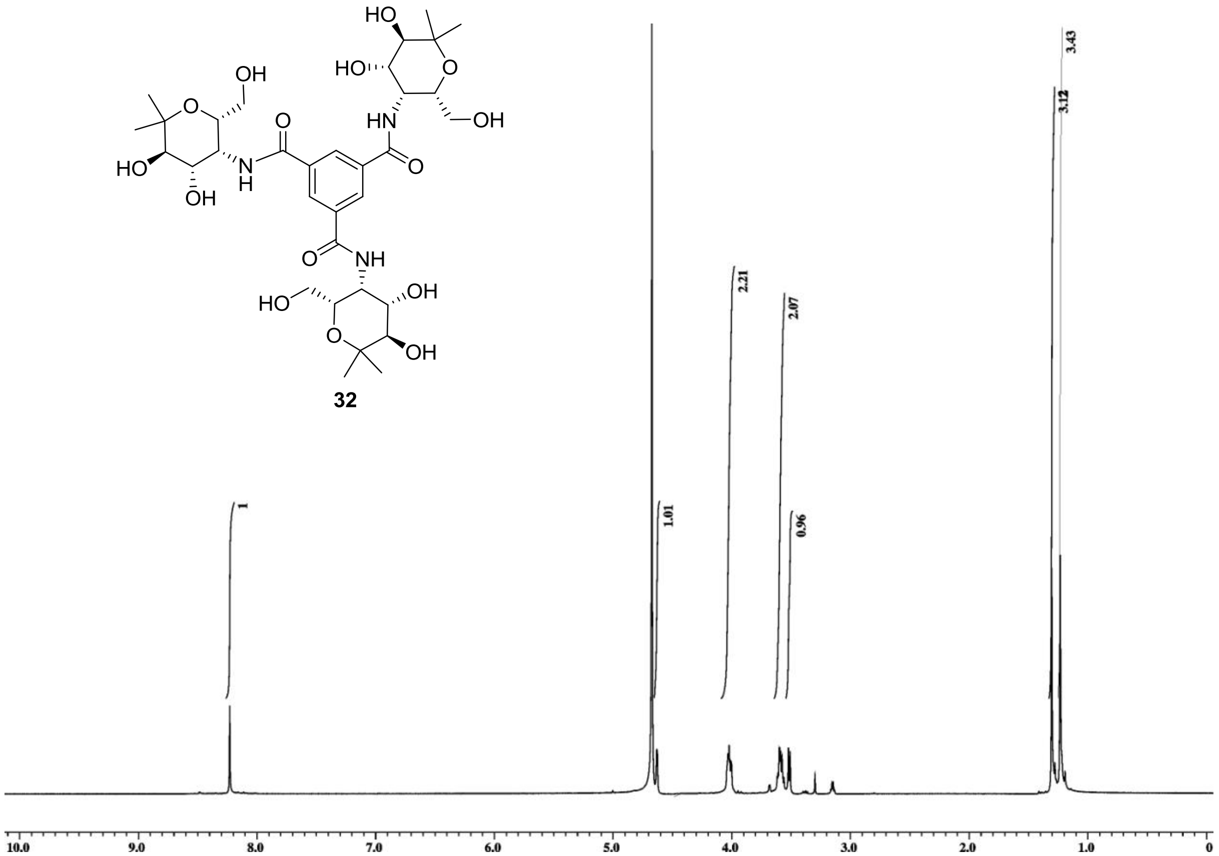

32

176 MHz, D_2O:

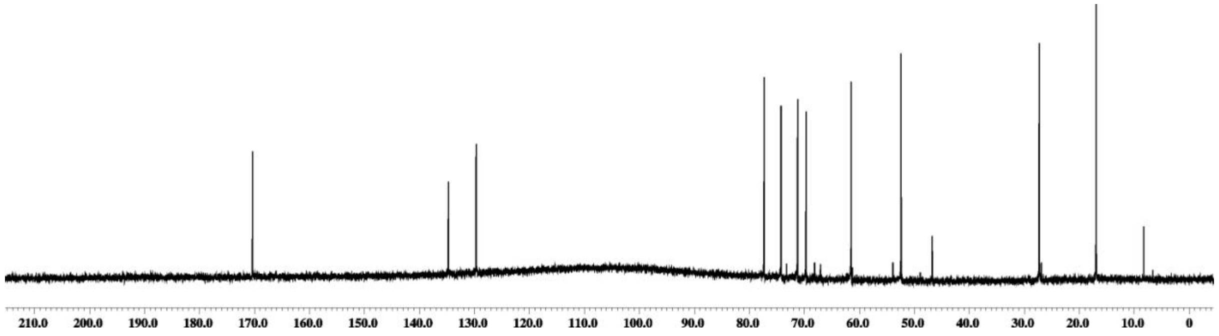

Supplement: File 1 — Experimental procedures, characterization data, 1H NMR and 13C NMR spectra of synthesized compounds. [file Beilstein_J_Org_Chem-06-75-s001.pdf]
